# Dosage Compensation throughout the *Schistosoma mansoni* Lifecycle: Specific Chromatin Landscape of the Z Chromosome

**DOI:** 10.1093/gbe/evz133

**Published:** 2019-07-03

**Authors:** Marion A L Picard, Beatriz Vicoso, David Roquis, Ingo Bulla, Ronaldo C Augusto, Nathalie Arancibia, Christoph Grunau, Jérôme Boissier, Céline Cosseau

**Affiliations:** 1 Université de Perpignan Via Domitia, IHPE UMR 5244, CNRS, IFREMER, Université de Montpellier, Perpignan, France; 2 Institute of Science and Technology Austria, Klosterneuburg, Austria

**Keywords:** dosage compensation, chromatin landscape, histone modifications, female heterogamety, *Schistosoma mansoni*

## Abstract

Differentiated sex chromosomes are accompanied by a difference in gene dose between X/Z-specific and autosomal genes. At the transcriptomic level, these sex-linked genes can lead to expression imbalance, or gene dosage can be compensated by epigenetic mechanisms and results into expression level equalization. *Schistosoma mansoni* has been previously described as a ZW species (i.e., female heterogamety, in opposition to XY male heterogametic species) with a partial dosage compensation, but underlying mechanisms are still unexplored. Here, we combine transcriptomic (RNA-Seq) and epigenetic data (ChIP-Seq against H3K4me3, H3K27me3, and H4K20me1 histone marks) in free larval cercariae and intravertebrate parasitic stages. For the first time, we describe differences in dosage compensation status in ZW females, depending on the parasitic status: free cercariae display global dosage compensation, whereas intravertebrate stages show a partial dosage compensation. We also highlight regional differences of gene expression along the Z chromosome in cercariae, but not in the intravertebrate stages. Finally, we feature a consistent permissive chromatin landscape of the Z chromosome in both sexes and stages. We argue that dosage compensation in schistosomes is characterized by chromatin remodeling mechanisms in the Z-specific region.

## Introduction

Sex determination systems are very diverse and can involve genetic and/or epigenetic based mechanisms (Bachtrog et al. 2014). Genetic sex determination has been widely studied and often involves well-differentiated pairs of sex chromosomes: X and Y in male heterogametic systems, or Z and W in female-heterogametic systems. Morphological and gene content differences arise between the sex-specific Y/W and the shared X/Z chromosomes after their evolution from an ancestral pair of autosomes (Charlesworth 1991; Charlesworth et al. 2005). Successive events of recombination suppression (Rice 1987; Bergero and Charlesworth 2009) result in the accumulation of deleterious mutations and lead to the degeneration of the Y/W heterochromosome (Engelstadter 2008). Consequently, the sex carrying the degenerated Y/W harbors a certain number of genes in a single X/Z-linked copy. In the absence of mechanisms to buffer expression levels, such monosomy in a diploid genome is expected to induce detrimental effects on finely tuned gene networks (Veitia 2005). Mechanisms of gene expression regulation have evolved independently across eukaryotes to compensate for this gene dose imbalance and are grouped together under the name “dosage compensation.” In the case of global dosage compensation, the overall expression level of monosomic genes on the heterochromosome (i.e., X/Z-specific genes) is equal to the overall diploid expression level of autosomal genes (i.e., X/AA or Z/AA ratio of 1, “A” standing for the autosomal expression) (Vicoso and Bachtrog 2009). Conversely, the gene dose can be only partially compensated and the average expression of monosomic X/Z-specific genes will then be below the average expression of the diploid autosomes. This X/Z-specific gene expression value is typically not equal to half the dose of autosomal genes, as would be expected in the total absence of compensation, but instead reaches 60–80% of autosomal expression (i.e., X/AA or Z/AA ratio of 0.6–0.8). This is thought to reflect a combination of general buffering mechanisms (Stenberg et al. 2009) and/or the individual upregulation of dosage-sensitive sex-linked genes, that is, a “gene-by-gene” mechanism of compensation (Mank 2009). Global dosage compensation was initially thought to be not only 1) the rule but also 2) the preserve of XY systems. Both considerations are actually controversial, and many studies have challenged this canonical view. First, global compensation is not an absolute rule among XY species: initially, “chromosome-wide” compensation was described in both vertebrate and invertebrate XY model species, as well as a variety of non-model organisms (e.g., *Caenorhabditis elegans*: Lau and Csankovszki 2015; *Drosophila*: Georgiev et al. 2011; Pea aphid: Jaquiéry et al. 2013; Hemipteran: Pal and Vicoso 2015; Mammals: Nguyen and Disteche 2006; Livebearer fishes: Darolti et al. 2019; and other vertebrates, reviewed in Graves 2016). Thereafter, the multiplicity of studies carried in a broad range of species actually highlighted a lack of global dosage compensation in vertebrates as well as in invertebrate (Xiong et al. 2010; Mank 2013; Hurst et al. 2015; Gu and Walters 2017 for review). Instead other strategies were observed, such as a gene-by-gene mechanism with an increase of only some X-linked genes (Lin et al. 2012; Pessia et al. 2012; Albritton et al. 2014), the decreased expression of autosomal genes (Julien et al. 2012), or duplication-translocation of X-linked genes to autosomes (Hurst et al. 2015). Second, global compensation is not the preserve of XY systems: recent studies have shown that non-model ZW species also display total equalization of expression between Z-specific genes and autosomes (Lepidoptera: Walters and Hardcastle 2011; Kiuchi et al. 2014; Gu and Walters 2017; Huylmans et al. 2017; *Artemia franciscana* crustacean species: Huylmans et al. 2019). This is in contradiction to earlier studies in which only partial compensation was documented in many ZW female heterochromatic clades: birds (Itoh et al. 2007; Naurin et al. 2011; Wolf and Bryk 2011; Uebbing et al. 2015), arthropods (Harrison et al. 2012; Mahajan and Bachtrog 2015), snakes (Vicoso et al. 2013), flatfish *Cynoglossus semilaevis* (Chen et al. 2014), and metazoan parasites of the genus *Schistosoma* (Vicoso and Bachtrog 2011; Picard et al. 2018).

Schistosomes are blood flukes responsible for schistosomiasis, an infectious disease affecting more than 230 million people worldwide (Colley et al. 2014, for review). The model species *Schistosoma mansoni* has a complex life cycle, characterized by 1) clonal multiplication in a freshwater snail of the *Biomphalaria* genus, the intermediate host and 2) sexual reproduction in a definitive vertebrate host (i.e., a primate or rodent species). The parasite’s eggs are released in freshwater via the feces. Free-living larvae (*miracidia*) hatch out and infect the mollusk intermediate host, transforming into sporocysts. Sporocyst clonal multiplication inside the mollusk ultimately leads to thousands of infective cercariae, which are in turn released into fresh water. Definitive host penetration occurs through the epidermis and is followed by drastic morphological and physiological transformations: 1) within 2 hours, the free-living larvae become obligatory endoparasitic schistosomula; 2) after 2–5 weeks within the definitive host, these schistosomula develop from 150-μm juvenile sexually undifferentiated individuals into 1-cm adults. *Schistosoma* is the only genus displaying separate sexes among flatworms (Basch 1990; Combes 1991). Sex is genetically determined, with ZZ males and ZW females (Grossman et al. 1981), but no apparent phenotypic sexual dimorphism exists from the egg to the early stages of schistosomula. Phenotypic differences between males and females (i.e., sexual dimorphism) appear only in late intravertebrate stages (Loker and Brant 2006). In addition, male and female worms remain immature (i.e., sexually nonfunctional) until pairing. In particular, mated females undergo extensive morphological changes and develop their reproductive organs (Erasmus 1973; Neves et al. 2005).

Schistosomes, and particularly *S. mansoni*, have well-differentiated sex chromosomes: the W chromosome is mostly heterochromatic and carries many repetitive sequences (Portela et al. 2010; Lepesant et al. 2012; Protasio et al. 2012); 1,067 Z-specific genes have been identified thus far (Protasio et al. 2012; Picard et al. 2018). These Z-specific genes are consistently male-biased in expression, which was initially interpreted as absence of a global mechanism of dosage compensation (Vicoso and Bachtrog 2011). It was recently suggested that instead schistosomes have partially upregulated their Z chromosome in response to gene loss on the W (Picard et al. 2018), but that this upregulation occurred in both sexes (thereby partially balancing dosage in females, but maintaining the male-bias in expression). The conclusions of these studies were limited by several drawbacks. First, only older, vertebrate-infecting, stages were considered. Gene balance is thought to be most crucial in the earlier stages of development because of a higher number of functional interactions (Cutter and Ward 2005; Davis et al. 2005; Artieri et al. 2009; Mank and Ellegren 2009), and missing these early stages could lead to an underestimation of the extent to which compensation occurs. Similarly, male-biased expression can be due to genes with male-specific functions, which should largely be absent before the onset of sexual differentiation. Characterizing the sex-bias of Z-specific genes throughout development is therefore crucial to fully understand the dynamics of dosage compensation in this group. Second, fully distinguishing between local and global mechanisms of compensation requires an understanding of the molecular processes at work. In particular, well-studied chromosome-wide mechanisms of dosage compensation are characterized by global and extensive changes in the epigenetic landscape of the affected sex chromosome (Lucchesi et al. 2005; Lucchesi 2018). For instance, active chromatin marks are strongly enriched on the *Drosophila* male X, whereas the inactivated mammalian X chromosome of females is characterized by DNA methylation and widespread repressive chromatin marks (Lucchesi et al. 2005; Brockdorff and Turner 2015). On the chicken Z chromosome, a local and female-specific hyperacetylation of the fourth histone (H4K16Ac) has been described (Bisoni et al. 2005). Apart from this study, the detailed characterization of sex-linked histone chromatin marks is entirely lacking in ZW species, severely limiting our mechanistic understanding of dosage equalization in these groups.

Here, we systematically assess gene expression regulation along the Z chromosome of *S. mansoni* by combining transcriptomic (RNA-Seq) and epigenomic (ChIP-Seq) data. We focus on three different developmental stages: 1) cercariae (RNA-Seq and ChIP-Seq): these free larvae are the last fully sexually undifferentiated stage before host penetration; 2) schistosomula (RNA-Seq): the schistosomula were at an advanced stage of development but lacked phenotypic dimorphism (Picard et al. 2016); and 3) immature worms (RNA-Seq and ChIP-Seq): male and female parasite displaying sexual dimorphism, but sexually immature as they were not mated.

## Materials and Methods

### Publicly Available RNA-Seq and ChIP-Seq Reads

This study is mainly based on publicly available data that were previously generated in our laboratory. Transcriptomic data (i.e., total RNA isolation followed by single-read 50 nucleotides Illumina sequencing) for both sexes in cercariae, schistosomula (stage S#2), and immature worms were described in Picard et al. 2016 (raw reads are available under accession number SRP071285 on NCBI-SRA database). As for epigenetic data (i.e., chromatin immunoprecipitation, or ChIP, followed by single-read 50 nucleotides Illumina sequencing): H3K27me3 ChIP-Seq was analyzed in Picard et al. 2016 (male data available under accession number SRP071285); H3K27me3, H3K4me3, and H3K20me1 ChIP-Seq in females were reported in Roquis et al. 2016 and Roquis et al. 2018 (all data available on SRP035609). Accession numbers for each studied library are provided in table 1.

**Table 1:** Publicly Available RNA-Seq and ChIP-Seq Data Used in This Study

Males	ChIP-Seq, SRP071285				RNA-Seq, SRP071285
	H3K4me3	H3K27me3	H4K20me1	Unbound	
Cercariae	SRX1631014	SRX1630978	—	SRX1631082	SRX1623575
	SRX1630986	SRX1630768	—	SRX1631034	SRX1619495
Schistosomula	—	—	—		SRX1630045
	—	—	—		SRX1630044
Immature worms	SRX1631176	SRX1631108	—	SRX1631199	SRX1630049
	SRX1631160	SRX1631129	—	SRX1631198	SRX1630048

**Females**	**ChIP-Seq, SRP035609**				**RNA-Seq, SRP071285**
	**H3K4me3**	**H3K27me3**	**H4K20me1**	**Unbound**	

Cercariae	SRX1592114	SRX1592113	SRX1592115	SRX1592116	SRX1630050
	SRX1592110	SRX1592109	SRX1592111	SRX1592107	SRX1630051
	SRX1592105	SRX1592103	SRX1592106	SRX1592102	—
Schistosomula	—	—	—	—	SRX1630059
	—	—	—	—	SRX1630060
Immature worms	SRX1592144	SRX1592143	SRX1592145	SRX1592146	SRX1630064
	SRX1592139	SRX1592138	SRX1592140	SRX1592141	SRX1630063
	SRX1592134	SRX1592133	SRX1592135	SRX1592136	—

Note.—RNA-Seq and ChIP-Seq data were previously generated in our laboratory and were published in two different studies: SRP071285 accession number (SRA-NCBI database) for RNA-Seq and male H3K27me3 ChIP-Seq duplicates (Picard et al. 2016); and SRP035609 accession number (SRA-NCBI database) for female ChIP-Seq triplicates (Roquis et al. 2016). H3K4me3 ChIP-Seq analysis on males was never presented before and was deposited under SRP071285 accession number (SRA-NCBI database).

### Newly Generated H3K4me3 ChIP-Seq Data in Males

Two biological replicates of male cercaria (2× 10,000 individuals) and immature worms (2× 20 individuals) were respectively obtained by monoclonal infection of *Biomphalaria glabrata* followed by unisex infection of Swiss OF1 mice (Picard et al. 2016 for details). Native chromatin immunoprecipitation assay was done according to Cosseau et al. (2009) using 4 µl of H3K4me3 antibody (Millipore, cat. number 04-745, lot number NG1680351). Further details are available at http://methdb.univ-perp.fr/epievo/; Last accessed June 27, 2019. ChIP library construction and sequencing were performed at the sequencing facilities of Montpellier GenomiX (MGX, France). Briefly, TruSeq ChIP sample preparation kit (Illumina Inc., USA) was used according to the manufacturer’s recommendations on 30 ng of DNA per condition. DNAs were blunt ended and adenylated on 3′ ends. Illumina’s indexed adapters were ligated to both ends, and resulting ligated DNA were enriched by polymerase chain reaction (PCR). PCR products were separated by size using electrophoresis and 400 base pairs (bp) fragments were selected. The quantitative analysis of the DNA library was carried on Agilent High Sensitivity chip and qPCR (Applied Biosystems 7500, SYBR Green). Finally, the sequencing was performed on a HiSeq2500 in single-read 50-nt mode.

### RNA-Seq Raw Read Processing

Raw RNA-Seq reads were cleaned using trimmomatic v0.3 (Bolger et al. 2014), with the following options: SE -phred33 ILLUMINACLIP:∼/Trimmomatic-0.36/adapters/TruSeq3-SE.fa:2:30:10 HEADCROP:12 LEADING:3 TRAILING:3 SLIDINGWINDOW:4:15 MINLEN:36. Resulting high quality reads were mapped to the *S. mansoni* reference genome v5.2 obtained on the WormBase Parasite database (schistosoma_mansoni.PRJEA36577.WBPS9.genomic.fa, at https://parasite.wormbase.org/index.html; Last accessed June 27, 2019) using Tophat2 (Trapnell et al. 2009), with the following options: –library-type fr-firststrand –microexon-search -i 10 -I 40000 –min-segment-intron 10 –max-segment-intron 40000. Mapped reads were counted using Htseq (Anders et al. 2015), with the following options: -f bam -s reverse -m union –idattr gene_id (see read counts in supplementary Data 1, Supplementary Material online). Normalized expression values (in Reads Per Kilobase of transcript per Million mapped reads, RPKM) were calculated for all genes and samples (supplementary Data 2, Supplementary Material online).

### Gene Expression Analysis According to Genomic Location

Gene location was based on GTF annotations obtained at WormbaseParasite (schistosoma_mansoni.PRJEA36577.WBPS9.canonical_geneset.gtf, https://parasite.wormbase.org/index.html; Last accessed June 27, 2019). More precisely, genes located on the ZW sex chromosome linkage map were then attributed either to the Z-specific region or to pseudoautosomal regions (PARs) depending on their coordinates (Z-specific windows: Z1: 3,550,000–13,340,000; Z2: 13,860,000–19,650,000; Z3: 23,230,000–30,820,000, supplementary Data 3, Supplementary Material online) (Protasio et al. 2012). Normalized expression data combined to gene locations were then implemented into an R script for drawing gene expression patterns (supplementary Data 4, Supplementary Material online). This R script is available as supplementary Methods 1, Supplementary Material online. Briefly, a Loess Normalization (R library Affy) was performed, taking into account the 12 libraries (cercariae, schistosomula, immature worms, for both sexes, and in duplicate). For each sex and stage, the averaged expression was then considered. Different thresholds for minimum of expression level were applied (RPKM > 0, RPKM > 1, and RPKM > 5), and strong sex-bias were considered or not. These strong sex-bias were defined by a fold change higher than 2, considering either the female-to-male ratio (female sex-bias), or the male-to-female ratio (male sex-bias). When comparing two conditions, the ratio between the medians of expression was calculated, and the level of significance was tested with Wilcoxon rank sum tests with continuity correction. In the figures, significance is showed by stars: *P value < 0.05, **P value < 0.001, and ***P value < 0.0001.

### ChIP-Seq Raw Read Processing

ChIP-Seq data treatment was carried out under a local galaxy instance (Goecks et al. 2010). After quality check (Andrews 2010), neither quality filtering nor trimming was applied and all the reads were mapped to the *S*. *mansoni* reference genome (assembly version 5.2) (Protasio et al. 2012), using Bowtie2 (Langmead and Salzberg 2012). Mapping quality in Bowtie 2 is related to “uniqueness” of the read. SAM alignment files were converted into the bed format with pyicos (Althammer et al. 2011) and sorted with sortBed -i of the bedtools suite (Quinlan et al. 2011).

### Comparative EpiChIP Analysis

Average histone modification profiles around transcriptional start site (TSS) of the genes were generated by doing a window analysis from −1,000 to +5,000 base pairs relative to this TSS, using EpiChIP v0.9.7-e (Hebenstreit et al. 2011). As input, we used the 23 million, 6 million, and 19 million randomly sampled mapped reads that were generated after the alignment step for H3K27me3, H3K4me3, and H4K20me1, respectively. The average histone profiles were generated on the chromosome 1 and independently on the Z-specific region and the PAR of the ZW sex chromosomes. For this purpose, we used the GTF annotation file generated previously (Picard et al. 2016) (available under id “*Schistosoma mansoni* sex-specific transcriptome” at http://ihpe.univ-perp.fr/acces-aux-donnees/; Last accessed June 27, 2019) and selected 6,225 transcripts on chromosome 1, 2,421 transcripts (= 2421) on the Z-specific region, and 3,376 on the PAR. The average H3K4me3, H3K27me3, and input profiles (i.e., control without antibody) were generated for the two male biological replicates and the three female biological replicates (Supplementary Data 6 and 7, Supplementary Material online). The average H4K20me1, and input profiles were generated for the three female biological replicates (Supplementary Data 8, Supplementary Material online). Each average profile was normalized with its respective input average profile in order to be able to compare the different regions, whatever the number of genes in the considered region. The distribution of histone enrichment around the transcription start site was compared according to the stage and the genomic location using Kolmogorov–Smirnov two sample tests.

### Code Availability

R script for expression analysis is provided in supplementary Methods 1, Supplementary Material online.

### Results

#### Different Levels of Gene Expression Equalization throughout Development

**Fig. 1.**
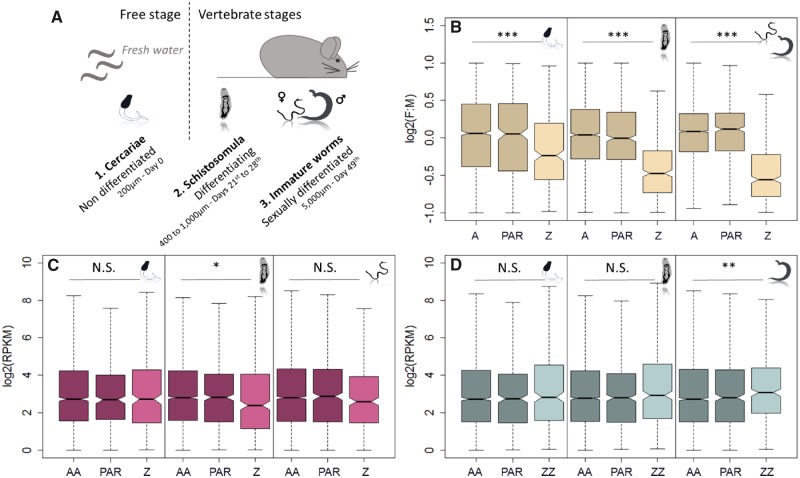
—Gene expression level according to sex, developmental stage, and genomic location. Three developmental stages of the parasite are shown (*A*): cercariae are free larvae without any sexual dimorphism (1); schistosomula represent an intravertebrate stage, with ongoing sexual differentiation but no phenotypic sexual dimorphism (2); and immature worms are sexually differentiated but sexually non-functional as they did not mate (3). Expression of autosomal genes (“A,” dark shade), pseudoautosomal genes (“PAR,” dark shade), and Z-specific genes (“Z,” light shade) is represented considering female-to-male ratio (“F:M”) (*B*), or independently within females (*C*) and males (*D*). Only genes with expression RPKM > 1, and a female-to-male fold change lower than 2 (sex-bias filtering) are taken into account (*n* = 3,741 in cercariae; *n* = 5,636 in schistosomula; *n* = 5,657 in immature worms). The Z-to-autosome expression ratio for each sex (Z:AA for female, and ZZ:AA for male), and the corresponding female-to-male ratio (Z[F:M]/A[F:M]) are detailed in table 2 (“RPKM > 1 - sex-bias filtered”). Asterisks show the level of significance for each of these comparisons (Wilcoxon test): *P value < 0.05, **P value < 0.001, and ***P value < 0.0001.

Gene expression was analyzed by using published RNA-Seq data from male and female *S. mansoni* of three developmental stages, characterized by different parasitic status and levels of sexual differentiation (Picard et al. 2016): cercariae, schistosomula, and immature worms (Fig. 1*A* ). Other stages such as *miracidia* or eggs were excluded because it is not possible to distinguish males and females at this phase. To assess dosage compensation, we compared both the Z-to-autosome ratio within each sex (Z:AA in females or ZZ:AA in males), and the female-to-male ratio between sexes (Z[F:M]/A[F:M]) (table 2). Such comparisons support global dosage compensation if the female Z:AA ratio and the Z(F:M)/A(F:M) ratio are equal to one; lower ratios support a lack of global dosage compensation. Different minimum expression thresholds were applied, and strongly sex-biased genes (>2-fold difference between the sexes) were excluded, as the presence of genes with sex-specific functions can also lead to male-biased expression of sex chromosomes, even in the presence of global dosage compensation (Huylmans et al. 2017). Gene expression ratios and their level of significance are detailed in table 2 for the three stages and six different methodological conditions. In order to minimize noise but keep a reasonable sample size, we focused on results obtained with a minimum expression threshold of RPKM > 1 and excluding genes with a sex-bias greater than 2-fold (Sample sizes are reported in Supplementary Table 1, Supplementary Material online), but qualitative patterns generally hold for other filtering procedures.

**Table 2:** Ratio of Gene Expression between Sexes, and between Genomic Locations within Each Sex; According to Developmental Stages, and Methodological Filters

		(*A*) ♀ Z:AA			(*B*) ♂ ZZ:AA			(*C*) Z(F:M)/A(F:M)		
		Cerc.	Som.	Ad.	Cerc.	Som.	Ad.	Cerc.	Som.	Ad.
**RPKM > 0**	Ratio	1.02	0.94	1.04	1.74	1.66	1.75	0.60	0.66	0.66
	*P* value	N.S.	N.S.	N.S.	***	***	***	***	***	***
**RPKM > 0, sex-bias filtered**	Ratio	1.05	0.75	1.00	1.48	1.10	1.34	0.82	0.76	0.66
	*P* value	N.S.	N.S.	N.S.	**	N.S.	*	***	***	***
**RPKM > 1**	Ratio	0.83	0.78	0.83	1.24	1.28	1.29	0.68	0.61	0.59
	*P* value	*	**	*	*	***	***	***	***	***
**RPKM > 1, sex-bias filtered**	Ratio	1.00	0.76	0.87	1.07	1.12	1.27	0.81	0.70	0.64
	*P* value	N.S.	*	N.S.	N.S.	N.S.	**	***	***	***
**RPKM > 5**	Ratio	0.92	0.76	0.82	1.23	1.42	1.24	0.74	0.62	0.60
	*P* value	N.S.	*	*	*	***	***	***	***	***
**RPKM > 5, sex-bias filtered**	Ratio	1.03	0.90	0.80	1.22	1.41	1.17	0.90	0.72	0.64
	*P* value	N.S.	N.S.	*	N.S.	*	*	**	***	***

Note.—The Z-to-autosome ratio are shown for females “Z:AA” (*A*) and males “ZZ:AA” (*B*) in cercariae (Cerc.), schistosomula (Som.), and immature worms (Ad.). Female-to-male ratio of the Z-specific genes and of the autosomes are then compared for the three same stages “Z(F:M)/A(F:M)” (*C*). The filter on sex-bias excludes genes with a fold change of expression above 2 in both directions (male:female or female:male). The level of significance for each comparison is indicated by the asterisks (Wilcoxon test):

* *P* value < 0.05, **P value < 0.001, and ***P value < 0.0001.

As observed before (Vicoso and Bachtrog 2011; Picard et al. 2018), the female-to-male ratio of expression was always significantly lower on the Z than on the autosomes, and this seemed to be due to both reduced Z:AA expression in females (Z:AA from 0.75 to 1.05) and an excess of ZZ:AA expression in males (ZZ:AA from 1.07 to 1.75) (Picard et al. 2018). However, there were important differences between the developmental stages. First, the Z(F:M)/A(F:M) ratio ranged from 0.60 to 0.90 (table 2C). Although part of this variation was driven by the different filtering procedures, in every case the value was closer to 1 for cercariae, consistent with more extensive dosage compensation in early development. For instance, for Z-specific genes with RPKM > 1 and no strong sex-bias, Z(F:M)/A(F:M) was equal to 0.81, 0.70, and 0.64 in cercariae, schistosomula, and immature worms, respectively, with significant differences between the free larval stage cercariae and the intravertebrate stages (*P* value < 0.0001, fig. 1*B*).

This difference in the extent of dosage equalization was also apparent within one sex. In the females, discrepancies were observed depending on the parasitic status and the filtering method: for RPKM > 0, after sex-bias filtering, the Z-to-autosome ratio (Z:AA) ranged from 0.75 in female schistosomula (lower than 1, consistent with partial dosage compensation) to 1.05 in female cercariae (equalized ratio, typical of dosage global compensation) and was not always significant from 1 (table 2A). This seemed to be largely driven by the dichotomy between cercariae and intravertebrate stages (fig. 1*C*): when considering genes with RPKM values above 1, and after filtering for sex-biased genes, Z-specific genes and autosomes displayed an equalized expression in female cercariae (Z:AA = 1.00 for RPKM > 1, Z:AA = 1.03 for RPKM > 5), but not in female schistosomula or immature worms (Z:AA = 0.76 and 0.87 for RPKM > 1, Z:AA = 0.90 and 0.80 for RPKM > 5). Although the specific numbers varied, cercariae had the highest female Z:AA expression ratio for all but one filtering procedures (RPKM > 0 and no removal of strongly sex-biased genes), generally supporting the more extensive upregulation of expression of Z-linked genes in females at this stage. Within males, the Z-to-autosome ratio (ZZ:AA) was higher in all studied conditions (ranging from 1.07 to 1.75) although the level of significance varied depending on the filtering procedure (table 2B). For instance, considering genes with RPKM > 1 and no strong sex-bias, the ZZ:AA ratios are 1.07, 1.12, and 1.27 for cercariae, schistosomula, and immature worms, respectively (fig. 1*D*).

In summary, this first transcriptomic analysis of Z-specific and autosomal genes throughout schistosome development showed that 1) the overexpression of the male Z-specific genes was found consistently in the three stages; 2) the female Z-to-autosome ratio was around 1 in cercariae, as expected for global dosage compensation; and 3) the female Z-to-autosome ratio was around 0.8 in the intravertebrate stages, as expected when partial dosage compensation occurs.

#### Regional Variation of Dosage Compensation along the Z-Specific Region

**Fig. 2.**
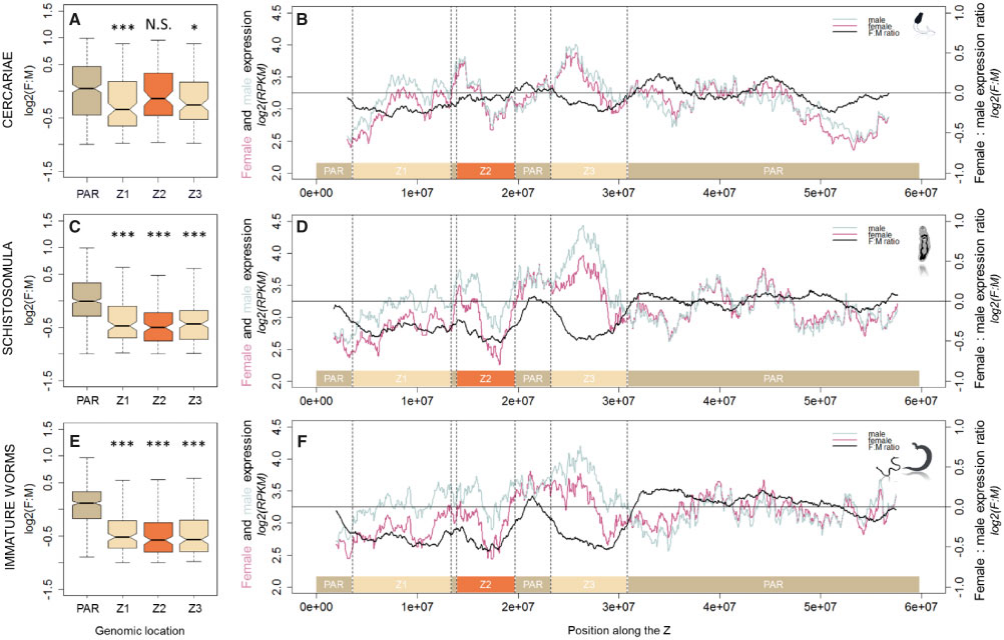
—Gene expression pattern according to the location along the Z chromosome, and the developmental stages. The female-to-male expression ratio (F:M) is represented for the three Z-specific regions defined in the version 5.2 of the genome (Z1 in light beige, Z2 in orange, and Z3 in light beige) and the PAR (in dark beige) for cercariae (*A*), schistosomula (*C*), and immature worms (*E*). For each stage, gene expression pattern for female (in pink) and male (in blue) is shown along the Z by sliding window of 50 genes (*B*, *D*, *F*). The thick black line represents the female-to-male expression ratio by sliding window of 50 genes. Only genes with expression RPKM > 1 and a sex-bias fold change <2 are shown. Asterisks show the level of significance of Z-to-PAR comparisons (Wilcoxon rank sum test with continuity correction): ***P value* *<* *0.0001, N.S. = nonsignificant differences. Z1-to-PAR ratio values are 0.76, 0.72, and 0.64; Z2-to-PAR ratio values are 0.88, 0.71, and 0.62; Z3-to-PAR ratio values are 0.81, 0.74, and 0.62, for cercariae, schistosomula, and immature worms, respectively. Other ratios are shown in Supplementary Table 2, Supplementary Material online.

In some species which display partial dosage compensation, the female-to-male ratio has been shown to vary along the Z chromosome, with some regions fully compensated, whereas others are not compensated at all (e.g., *Gallus gallus* [Melamed and Arnold 2007] and *Cynoglossus semilaevis* [Shao et al. 2014]). To investigate potential regional variation in *S.mansoni*, we investigated local patterns of gene expression by using sliding windows of 50 genes along the Z chromosome (fig. 2). The Z-specific part, which was previously described as discontinuous in the version 5.2 of the genome (Protasio et al. 2012), was considered here as three Z-specific regions named Z1, Z2, and Z3. They were respectively defined by their coordinates, as follow: Z1 from 3,550 to 13,340 kb; Z2 from 13,860 to 19,650 kb; and Z3 from 23,230 to 30,820 kb (Protasio et al. 2012).

As expected, the sliding window analysis revealed a female-to-male ratio oscillating around 1 in the PAR for all stages (log 2[F:M] close to 0; fig. 2, right panel). In the three Z-specific regions, the female-to-male expression ratio oscillated around 0.7 in schistosomula and 0.6 immature worms, consistent with homogeneous but partial compensation (fig. 2*D* and *F*;supplementary figs. 1 and 2, Supplementary Material online). However, in cercariae, the female-to-male expression ratio was closer to 1. When looking at each Z-specific region individually, the female-to-male expression ratio was significantly lower in the Z1 and Z3 regions compared with the PAR in all three developmental stages (fig. 2 left panel; values and significance in Supplementary Table 2, Supplementary Material online). On the other hand, the Z2 region displayed a higher female-to-male ratio in cercariae, and this was not significantly different from the PAR (Z2[F:M]/PAR[F:M] = 0.88; fig. 2*A*). In schistosomula and immature worms, the same Z2 was significantly more male biased in expression than the PAR (Z2[F:M]/PAR[F:M] = 0.71 and 0.62, respectively; fig. 2*C* and *E*).

**Fig. 3.**
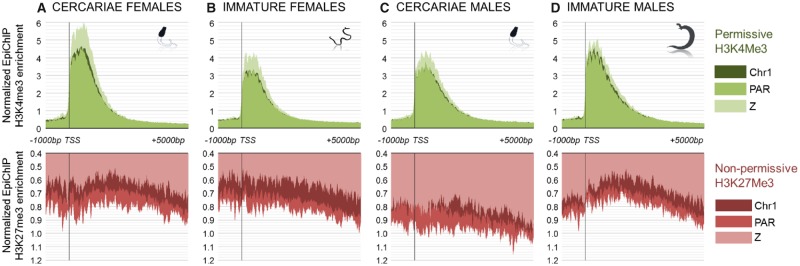
—Average H3K4me3 and H3K27me3 enrichment profile according to sex, developmental stage, and genomic location. *x* axis represents the position in base pairs (bp) relative to the TSS of the genes (position 0). *y* axis represents the normalized average enrichment of reads obtained after a Chromatin Immunoprecipitation targeting the “permissive” mark H3K4me3 (in green) and the “nonpermissive” H3K27me3 (in red), in female cercariae (*A*) and immature worms (*B*), or male cercariae (*C*) and immature worms (*D*). The EpiChIP enrichment has been calculated around the TSS for chromosome 1 as proxy for autosomes (Chr1, dark shade), for the PAR of sex chromosomes (PAR, medium shade), and for chromosome Z-specific region of sex chromosomes (Z, light shade). For each of these genomic locations, we show the average result of the profiles obtained for each coding sequence. Each profile has been normalized with the same average enrichment of reads obtained after a Chromatin Immunoprecipitation without antibody. The experiment was performed in duplicates in males and triplicate in females. EpiChIP profiles showing standard error at each position are shown in Supplementary Figures 6 and 7, Supplementary Material online. The percentage of maximum difference between genomic regions is shown in Supplementary Tables 3 and 4, Supplementary Material online: all differences are statistically significant (*P* value < 0.001, Kolmogorov–Smirnov two sample tests).

Consistent with this, male and female expression levels overlapped in the Z2 region of cercariae; whereas in the Z1, female expression was lower than male expression, consistent with less complete compensation (fig. 2*B*). In the Z3 region, and still in cercariae, we found a narrow window of overlapping male and female expression at the beginning of the region, whereas the remaining part displayed lower expression in females. This could be meaningful because Z3 shows an intermediate Z(F:M)/PAR(F:M) ratio (0.76*** < 0.81* < 0.88^N.S.^ for Z1 < Z3 < Z2), which could be driven by these smaller-scale regional differences (fig. 2*B*). In the intravertebrate stages, the three Z-specific regions differed clearly from the pseudoautosomal ones by presenting consistently reduced expression in females (fig. 3*D* and *F*). The female and male gene expression ratios for each Z-specific region are shown in Supplementary Table 2, Supplementary Material online, and the patterns of expression within each sex are illustrated in Supplementary Figure 3, Supplementary Material online. Considering gene-by-gene comparison between stages, intravertebrate stages displayed more similar expression patterns than cercariae (Supplementary Fig. 4, Supplementary Material online).

In summary, this transcriptomic analysis along the Z chromosome highlighted 1) local variation of the dosage compensation across Z-specific regions in cercariae and 2) partial and consistent dosage compensation all along the three Z-specific regions in schistosomula and immature worms.

#### Z-Specific Chromatin Landscape in Cercariae and Immature Worms to Elucidate Shared or Sex-Specific Mechanisms

Previous studies on dosage compensation in model organisms indicate that chromatin structure plays a key role in the regulation of this evolutionary mechanism (Lucchesi et al. 2005; Lucchesi 2018). As the main transcriptomic differences were observed between the free stage cercariae and the intravertebrate stages, we focused our epigenetic study on cercariae and immature worms. We analyzed immunoprecipitation assays with antibodies targeting three histone marks: 1) H3K4me3, associated with active promoter and transcription start site, and depleted in the heterochromatic inactivated X chromosome of mammals (O’Neill et al. 2008; Marks et al. 2009); 2) H3K27me3, a repressive mark associated with polycomb, poised transcription, and enriched in the inactivated X chromosome of mammals (Lucchesi et al. 2005); and 3) H4K20me1, associated with nonpermissive chromatin in *Caenorhabditis* dosage compensation (Vielle et al. 2012; Kramer 2015, 2016). We compared the enrichment of these histone marks in a sex-specific manner, on Z-specific genes, pseudoautosomes, and autosomes (using chromosome 1 as a proxy for the autosomal chromatin landscape, as its transcriptomic pattern appeared to be representative of autosomes, see Supplementary Figure 5, Supplementary Material online). For each of these genomic locations, the average enrichment profile of all annotated genes was performed around the TSS: from 1,000 base pairs (bp) upstream to 5,000-bp downstream.

We observed that the average profile of the three studied marks was specific for each genomic location in both cercariae and immature worms (Figure 3 and supplementary figs. 6–8, Supplementary Material online). Z-specific genes were enriched for H3K4me3 in both stages and sexes, especially between the TSS and position +2,000 bp (fig. 3, upper panel). H3K27me3 was depleted from Z-specific genes upstream of the TSS and along the transcription unit in both stages and sexes (fig. 3, lower panel). Finally, H4K20me1, for which only female data was available, displayed a stronger enrichment on chromosome 1 and on the PAR than on Z-specific genes in female cercariae (percentage of maximum difference: 63.3%, supplementary fig. 8 and supplementary table 5, Supplementary Material online). However, this difference diminished between stages, with an increased enrichment on the Z-specific region in immature females bringing it closer to the autosomal level (percentage of maximum difference: 30%, supplementary fig. 8 and supplementary table 5, Supplementary Material online). Differences between chromosomal regions within a developmental stage and sex were tested for the three analyzed histone marks: the most significant difference was consistently observed either between Z-specific genes and chromosome 1 or between Z-specific and pseudoautosomal genes (Kolmogorov–Smirnov two sample tests, supplementary tables 3–5, Supplementary Material online). This supports the idea that the chromatin landscape is more similar between the autosomes and the PAR of *S. mansoni*, whereas Z-specific genes display a singular epigenetic landscape. The depletion of the two “repressive” marks (H3K27me3 and H4K20me1) and the enrichment of the “permissive” H3K4me3 mark suggest a chromatin structure prone to enhanced gene expression specifically in this Z-specific region.

In summary, our epigenetic analysis highlighted the specialized chromatin landscape of the Z-specific region, which appeared to be more permissive than that of the autosomal regions in both sexes and stages. This modified chromatin state of the Z-specific region likely promotes enhanced expression compared with the autosomes, independent of the stage and sex, consistent with the first part of our study. The analysis of H4K20me1 in females further showed a differential enrichment of this histone mark on Z-specific genes between cercariae and adults, which may be related to the differences that we observe in the extent of female dosage compensation between the two stages.

## Discussion

We provide here the first combined transcriptomic and epigenetic analysis of Z chromosome dosage compensation through three developmental stages of *S. mansoni* parasite. This work highlights three important aspects of gene expression regulation on sex chromosomes: 1) A strong upregulation of female expression of Z-linked genes in the free larval stage cercariae, consistent with complete dosage compensation, but not in intravertebrate stages schistosomula and immature females that display partial dosage compensation only. 2) Local variations of the female-to-male expression ratio along the Z chromosome. 3) Differences in chromatin structures between Z-specific regions and autosomes which may support the enhanced expression of both male and female Z chromosomes.

### Change in Dosage Compensation Status following Host Penetration

Our RNA-Seq analysis detects a global hypertranscription of Z-specific genes in both sexes of *S. mansoni*, consistent with the intermediate stage of compensation previously reported in schistosomula and adult worms (Vicoso and Bachtrog 2011; Picard et al. 2018). In both studies, the female upregulation of expression only partially resolved the imbalance between the Z chromosome and the autosomes. However, we show here that this partial compensation does not apply to earlier developmental stages of *S. mansoni:* in females, enhanced transcriptional levels of the Z-specific genes results in a Z-to-A ratio of around 1 in cercariae (compared with ∼0.8 in the intravertebrate stages).

The dosage compensation status has been shown to be tissue- and development-specific within a same species (Mank and Ellegren 2009; Lott et al. 2011; Deng et al. 2014; Huylmans et al. 2017). In particular, germ/stem cells might need to overcome a special challenge regarding dosage compensation, as 1) genome wide reprograming is expected to occur and erase epigenetic marks responsible for maintaining the chromatin state necessary for compensation (Sangrithi and Turner 2018) and/or 2) dosage compensation mechanisms may act as developmental regulators by targeting autosomal genes involved in patterning and morphogenesis (Valsecchi et al. 2018). In schistosomes, larval stages display particular features regarding their content in such cells: in the intermediate host, sporocysts consist of totipotent stem cells undergoing intense clonal multiplication (Cort et al. 1954); in cercariae, embryonic stem cell specific combination of histone marks have been described (Wang et al. 2013; Roquis et al. 2015). This chromatin signature disappears after host penetration, signifying important changes in gene expression regulation (Roquis et al. 2015). Therefore, a specific cell type content could explain the shift in the dosage compensation status observed between the larval stage and the intravertebrate phase. Further studies are needed to disentangle the role of those cells in the observed pattern, and the examination of dosage compensation status during earlier larval stages certainly deserves further attention. Technological advances such a single cell/single individual RNA sequencing should in the future allow for such analyses.

### Local Variation of Male-Bias in Cercariae: Different Evolutionary Stages of the Dosage Compensation Mechanisms?

Overexpression of Z-specific genes compared with the level of autosomal gene expression has been recently described in *S. mansoni* intravertebrate stages (Picard et al. 2018). Here, we further show that this overexpression also occurs in the undifferentiated and free-living stage cercariae and is therefore a consistent feature maintained throughout the parasite life cycle. This uncommon feature has been described in adult floor beetles (whole body sample) which is a XY male heterogametic system (Prince et al. 2010). In this species, the male hemizygous X chromosome is fully compensated, and females display XX/AA ratio superior to 1. It has been also recently reported in the young XY plant system *Silene* latifolia (Muyle et al. 2018). Such pattern was theorized by Gu and Walters as the “type IV” of sex chromosome dosage compensation, with complete equalization of the X/Z-to-autosome ratio in the heterogametic sex, but no dosage balance between sexes as the homogametic sex exhibit Z/X hypertranscription relative to autosomes (Gu and Walters 2017). These observations can be interpreted as an example of the first step of the Ohno’s hypothesis, suggesting that the establishment of dosage compensation process evolved in a two-step process (Ohno 1967; Charlesworth 1996; Vicoso and Bachtrog 2009; Bachtrog et al. 2011; Mank 2013): 1) First, Z- or X-linked genes became upregulated in both sexes. This would restore expression of the single X or Z chromosome in the heterogametic sex to the diploid level that existed before degradation of the Y or W, but would result in too much gene expression in the homogametic sex. 2) Therefore, secondarily, a downregulation of Z or X-linked gene expression would evolve in the homogametic sex to restore the correct Z- or X-to-autosome ratio. But many studies recently challenge this theory. For instance, there is no convincing evidence that the X chromosome is globally upregulated in both mammals (Julien et al. 2012), and *C. elegans* (Albritton et al. 2014). In *C. elegans*, the X chromosomes in the XX homogametic individuals are downregulated, leading to dosage balance between the sexes but not dosage compensation in X0 males. In placental mammals, no global X upregulation was observed, unlike in marsupials (Julien et al. 2012; Whitworth and Pask 2016). Thus, even though complete X chromosome inactivation has traditionally been interpreted as the second step of the process (Brockdorff and Turner 2015), the relevance of the Ohno’s hypothesis to the evolution of placental mammal dosage compensation is in fact controversial (e.g., Nguyen and Disteche 2006; Vicoso and Bachtrog 2009; Lin et al. 2012; Mank 2013; Pessia et al. 2014; Gu and Walters 2017).

Our results suggest that *S. mansoni* may have followed an “Ohno-like” evolutionary trajectory for dosage compensation, which allowed the Z-specific region to enhance its expression to reach that of autosomal genes in females, but under which males somehow avoid the necessity of complete countercompensation of this Z chromosome hypertranscription. This raises the question of what the current status for the evolution of dosage compensation is in *S. mansoni*: an intermediate or a stable state?

Regional variation of the Z dosage compensation has been described in birds, where some dosage-compensated genes are concentrated in a region of the short arm of the Z chromosome, near the male hypermethylated (MHM) locus (Melamed and Arnold 2007; Wright et al. 2015). Full dosage compensation has also been described in a restricted chromosomal region in pseudomale testes of *Cy.semilaevis*, whereas the rest of the Z chromosome is partially compensated (Shao et al. 2014). It has been proposed that such local variations in gene regulation along the sex chromosome could be based on regional age following sex-linkage, including in XY species such as stickleback (Schultheiß et al. 2015). In *S. mansoni*, two evolutionary strata of different ages were recently described (supplementary fig. 9, Supplementary Material online) (Picard et al. 2018). If partial upregulation of Z-specific genes in both sexes represents an intermediate step in the evolution of dosage compensation, we may expect that the older stratum will be closer to balanced dosage between males and females than the younger one. In the previous study and in ours, no difference in gene expression could be detected between these strata in intravertebrate stages. In cercariae, however, the section of the Z that is closest to complete compensation (region Z2) is indeed part of the older Z-specific evolutionary stratum that is shared between African and Asian schistosomes (supplementary fig. 9, Supplementary Material online, Picard et al. 2018). It is therefore likely that dosage compensation is still evolving in *S. mansoni*, and that the Z-specific region which displays equalized gene expression between the sexes actually represents the final stage of dosage compensation evolution in this species.

### The Specific Chromatin Features of the Z-Specific Regions May Account for the Hypertranscription of Z-Specific Genes in Both Sexes

Various mechanisms have evolved to regulate the gene dosage at the functional level. In fully compensated organisms, these mechanisms are all based on the modulation of chromatin accessibility of the sex-specific regions (Lucchesi et al. 2005; Ercan 2015; Lucchesi 2018). In mammals, transcriptional regulation is achieved by the progressive depletion of histone active marks concomitant to the enrichment of histone repressive mark H3K27me3 through the action of the X-inactive specific transcript (Xist) (Brockdorff and Turner 2015). In *C.elegans* (XX/XO system), the hermaphrodite two X chromosomes display halved transcription level to match X expression to that of males (XO). Reduced transcription is allowed by a complex of proteins called the dosage compensation complex resulting in a depletion of histone active marks and enrichment of histone repressive marks (Lau and Csankovszki 2015). In *Drosophila melanogaster* (XX/XY system), the transcription of the single male X chromosome is doubled by an overall increase of the chromatin accessibility by the Male-Specific Lethal complex (Lucchesi et al. 2005). More recent work performed on the pea aphid has also shown an enhanced chromatin accessibility of the X chromosome of males which may account for dose correction of those X-linked genes in this species (Richard et al. 2017).

We present here an overview of the chromatin structure by ChIP-Seq analysis targeting three modified histones in different developmental stages of *S. mansoni* and highlight different chromatin patterns between autosomal and Z-linked genes. We found that H3K27me3 and H4K20me1 were depleted in the Z-specific region relative to the PAR of Z chromosome and to autosomal regions. Reversely, we found that H3K4me3 was enriched in the Z-specific region of the Z chromosome relative to the PAR and to autosomal regions. The role of H4K20me1 in gene regulation has remained a mystery because of its contribution to both gene activation and gene repression in different contexts (Beck et al. 2012). However, in the context of chromosome-wide regulatory mechanism for dosage compensation, this mark has been shown to be enriched in the inactive chromosome of female mammals (Kelsey et al. 2015) and enriched in both hermaphrodite X chromosomes in *Caenorhabditis*, resulting in reduction of transcription level (Bian et al. 2017; Kramer 2015, 2016). Here, changes in H4K20me1 between female cercariae and adults are specifically observed for the Z-specific region, arguing in favor of a role of this mark in dosage compensation status switch between the stages. H3K27me3 is a clear repressive mark enriched in heterochromatic X-inactivated chromosome of mammals (Kelsey et al. 2015) and H3K4me3 was reported to be depleted during X-inactivation in female embryonic stem cells (O’Neill et al. 2008). Given the depletion for both repressive marks concomitant to enrichment of the active H3K4me3 mark in the Z-specific region, we suggest that a global regulation of the chromatin accessibility of the Z-linked regions occurs in order to overexpress the Z-linked genes and compensate for gene dose defect in the single Z-specific region of females. This could be further addressed using chromatin accessibility assay which could evidence an open chromatin state such as those performed in the pea aphids which support their enhanced X overexpression (Richard et al. 2017).

Local variation in dosage compensation is also supported by chromatin based event such as those described in *C. semilaevis* where an increase in cytosine methylation density occurs in the compensated region (Shao et al. 2014). In *Gallus gallus*, the implication of the MHM noncoding RNA and subsequent enrichment of H4K16ac around the MHM locus in females allow a full compensation of this region (Melamed and Arnold 2007). Female-specific non-coding RNAs have been shown to be expressed in schistosome larval stages (Lepesant et al. 2012) and their implication in changes in chromatin states, regulation of gene expression and, more specifically, in dosage compensation mechanisms, certainly deserve further attention.

## Conclusion

Until recently, dosage compensation in ZW female-heterogametic species was thought to be partial and to occur at a gene-specific level. Recent studies in non-model organisms have challenged this canonical view. In line with them, our study brings a new insight by showing developmental changes in dosage compensation status in female schistosomes. From global compensation in undifferentiated free larvae, to partial compensation after host penetration and the onset of sexual differentiation. Despite this developmental variation, our study highlights a robust overexpression of the Z chromosome throughout *S. mansoni* life cycle, independently of the sex. We show how this might be mediated by an enhanced chromatin accessibility of the Z-specific regions, giving a first insight into *S. mansoni* chromatin pattern in relation to dosage compensation. Our epigenetic study paves the way toward the construction of an evolutionary chromatin landscape of the parasite’s dosage compensation. Investigating more combined histone modifications and non-coding RNAs appears to be the next step to understand both developmental changes and finer variations in gene expression along the Z chromosome.

## Supplementary Material

Supplementary data are available at *Genome Biology and Evolution* online.

## Ethic Statement

All experiments were carried out following the national ethical standards established in the writ of February 1, 2013 (NOR: AGRG1238753A), which set the conditions for approval, planning, and operation of establishments, breeders, and suppliers of animals used for scientific purposes and controls. The French Ministère de l’Agriculture et de la Pêche and the French Ministère de l’Education Nationale de la Recherche et de la Technologie provided permit A66040 to the laboratory for animal experiments and certificate to the experimenters (authorization 007083, decree 87–848).

## Supplementary Material

Supplementary_Material_evz133

## Notes

**Data deposition:** Male and female RNA-Seq reads H3K4me3 ChIPseq reads for males were deposited at the NCBI-SRA database under study accession number SRP071285.

## References

[evz133-B1] Albritton SE, et al 2014 Sex-biased gene expression and evolution of the X chromosome in nematodes. Genetics 197(3):865–883. 24793291 10.1534/genetics.114.163311 PMC4096367

[evz133-B2] Althammer S, González-Vallinas J, Ballaré C, Beato M, Eyras E. 2011 Pyicos: a versatile toolkit for the analysis of high-throughput sequencing data. Bioinformatics 27(24):3333–3340. 21994224 10.1093/bioinformatics/btr570 PMC3232367

[evz133-B3] Anders S, Pyl PT, Huber W. 2015 HTSeq—a Python framework to work with high-throughput sequencing data. Bioinformatics 31(2):166–169. 25260700 10.1093/bioinformatics/btu638 PMC4287950

[evz133-B4] Andrews S. 2010 FastQC: a quality control tool for high throughput sequence data. https://www.bioinformatics.babraham.ac.uk/projects/fastqc/, last accessed June 27, 2019.

[evz133-B5] Artieri CG, Haerty W, Singh RS. 2009 Ontogeny and phylogeny: molecular signatures of selection, constraint, and temporal pleiotropy in the development of *Drosophila*. BMC Biol. 7(1):42. 19622136 10.1186/1741-7007-7-42 PMC2722573

[evz133-B6] Bachtrog D, et al 2011 Are all sex chromosomes created equal? Trends Genet. 27(9):350–357. 21962970 10.1016/j.tig.2011.05.005

[evz133-B7] Bachtrog D, et al 2014 Sex determination: why so many ways of doing it? PLoS Biol. 12(7):e1001899. 24983465 10.1371/journal.pbio.1001899 PMC4077654

[evz133-B8] Basch PF. 1990 Why do schistosomes have separate sexes? Parasitol Today 6(5):160–163. 15463329 10.1016/0169-4758(90)90339-6

[evz133-B9] Beck DB, Oda H, Shen SS, Reinberg D. 2012 PR-Set7 and H4K20me1: at the crossroads of genome integrity, cell cycle, chromosome condensation, and transcription. Genes Dev. 26(4):325–337. 22345514 10.1101/gad.177444.111 PMC3289880

[evz133-B10] Bergero R, Charlesworth D. 2009 The evolution of restricted recombination in sex chromosomes. Trends Ecol Evol. 24(2):94–102. 19100654 10.1016/j.tree.2008.09.010

[evz133-B11] Bian Q, Anderson EC, Brejc K, Meyer BJ. 2017 Dynamic control of chromosome topology and gene expression by a chromatin modification. Cold Spring Harb Symp Quant Biol. 82:279–291. 29472317 10.1101/sqb.2017.82.034439 PMC6041165

[evz133-B12] Bisoni L, Batlle-Morera L, Bird AP, Suzuki M, McQueen HA. 2005 Female-specific hyperacetylation of histone H4 in the chicken Z chromosome. Chromosome Res. 13(2):205–214. 15861309 10.1007/s10577-005-1505-4

[evz133-B13] Bolger AM, Lohse M, Usadel B. 2014 Trimmomatic: a flexible trimmer for Illumina sequence data. Bioinformatics 30(15):2114–2120. 24695404 10.1093/bioinformatics/btu170 PMC4103590

[evz133-B14] Brockdorff N, Turner BM. 2015 Dosage compensation in mammals. Cold Spring Harb Perspect Biol. 7(3):a019406. 25731764 10.1101/cshperspect.a019406 PMC4355265

[evz133-B15] Charlesworth B. 1991 The evolution of sex chromosomes. Science 251(4997):1030–1033. 1998119 10.1126/science.1998119

[evz133-B16] Charlesworth B. 1996 The evolution of chromosomal sex determination and dosage compensation. Curr Biol. 6(2):149–162. 8673462 10.1016/s0960-9822(02)00448-7

[evz133-B17] Charlesworth D, Charlesworth B, Marais G. 2005 Steps in the evolution of heteromorphic sex chromosomes. Heredity (Edinb). 95(2):118–128. 15931241 10.1038/sj.hdy.6800697

[evz133-B18] Chen S, Zhang G, Shao C. 2014 Whole-genome sequence of a flatfish provides insights into ZW sex chromosome evolution and adaptation to a benthic lifestyle. Nat Genet. 46(3):253–260. 24487278 10.1038/ng.2890

[evz133-B19] Colley DG, Bustinduy AL, Secor WE, King CH. 2014 Human schistosomiasis. Lancet 383(9936):2253–2264. 24698483 10.1016/S0140-6736(13)61949-2 PMC4672382

[evz133-B20] Combes C. 1991 The schistosome scandal. Acta Oecol. 12:165–173.

[evz133-B21] Cort WW, Ameel DJ, Van der Woude A. 1954 Germinal development in the sporocysts and rediae of the digenetic trematodes. Exp Parasitol. 3(2):185–225. 13161963 10.1016/0014-4894(54)90008-9

[evz133-B22] Cosseau C, et al 2009 Native chromatin immunoprecipitation (N-ChIP) and ChIP-Seq of *Schistosoma mansoni*: critical experimental parameters. Mol Biochem Parasitol. 166(1):70–76. 19428675 10.1016/j.molbiopara.2009.02.015

[evz133-B23] Cutter AD, Ward S. 2005 Sexual and temporal dynamics of molecular evolution in *C. elegans* development. Mol Biol Evol. 22(1):178–188. 15371532 10.1093/molbev/msh267

[evz133-B24] Darolti I, et al 2019 Extreme heterogeneity in sex chromosome differentiation and dosage compensation in livebearers. bioRxiv. 1:589788. doi:10.1101/589788. 10.1073/pnas.1905298116 PMC6754558 31484763

[evz133-B25] Davis JC, Brandman O, Petrov DA. 2005 Protein evolution in the context of *Drosophila* Development. J Mol Evol. 60(6):774–785. 15909223 10.1007/s00239-004-0241-2

[evz133-B26] Deng X, Berletch JB, Nguyen DK, Disteche CM. 2014 X chromosome regulation: diverse patterns in development, tissues and disease. Nat Rev Genet. 15(6):367–378. 24733023 10.1038/nrg3687 PMC4117651

[evz133-B27] Engelstadter J. 2008 Muller’s ratchet and the degeneration of Y chromosomes: a simulation study. Genetics 180:957–967. 18780738 10.1534/genetics.108.092379 PMC2567394

[evz133-B28] Erasmus DA. 1973 A comparative study of the reproductive system of mature, immature and ‘unisexual’ female *Schistosoma mansoni*. Parasitology 67(2):165. 4795964 10.1017/s0031182000046394

[evz133-B29] Ercan S. 2015 Mechanisms of X chromosome dosage compensation. J Genomics. 3:1. 25628761 10.7150/jgen.10404 PMC4303597

[evz133-B30] Georgiev P, Chlamydas S, Akhtar A. 2011 *Drosophila* dosage compensation: males are from Mars, females are from Venus. Fly 5(2):147–154. 21339706 10.4161/fly.5.2.14934 PMC3127063

[evz133-B31] Goecks J, Nekrutenko A, Taylor J, The Galaxy Team. 2010 Galaxy: a comprehensive approach for supporting accessible, reproducible, and transparent computational research in the life sciences. Genome Biol. 11(8):R86. 20738864 10.1186/gb-2010-11-8-r86 PMC2945788

[evz133-B32] Graves J. 2016 Evolution of vertebrate sex chromosomes and dosage compensation. Nat Rev Genet. 17(1):33–46. 26616198 10.1038/nrg.2015.2

[evz133-B33] Grossman AI, Short RB, Cain GD. 1981 Karyotype evolution and sex chromosome differentiation in Schistosomes (Trematoda, Schistosomatidae). Chromosoma 84(3):413–430. 7327052 10.1007/BF00286030

[evz133-B34] Gu L, Walters JR. 2017 Evolution of sex chromosome dosage compensation in animals: a beautiful theory, undermined by facts and bedeviled by details. Genome Biol Evol. 9(9):2461–2476. 28961969 10.1093/gbe/evx154 PMC5737844

[evz133-B35] Harrison PW, Mank JE, Wedell N. 2012 Incomplete sex chromosome dosage compensation in the Indian meal moth, *Plodia interpunctella*, based on de novo transcriptome assembly. Genome Biol Evol. 4(11):1118–1126. 23034217 10.1093/gbe/evs086 PMC3514961

[evz133-B36] Hebenstreit D, et al 2011 RNA sequencing reveals two major classes of gene expression levels in metazoan cells. Mol Syst Biol. 7(1):497. 21654674 10.1038/msb.2011.28 PMC3159973

[evz133-B37] Hurst LD, Ghanbarian AT, Forrest AR FANTOM Consortium Huminiecki L. 2015 The constrained maximal expression level owing to haploidy shapes gene content on the mammalian X chromosome. PLoS Biol. 13(12):e1002315. 26685068 10.1371/journal.pbio.1002315 PMC4686125

[evz133-B38] Huylmans AK, Macon A, Vicoso B. 2017 Global dosage compensation is ubiquitous in Lepidoptera, but counteracted by the masculinization of the Z chromosome. Mol Biol Evol. 34(10):2637–2649. 28957502 10.1093/molbev/msx190 PMC5850747

[evz133-B39] Huylmans AK, Toups MA, Macon A, Gammerdinger WJ, Vicoso B. 2019 Sex-biased gene expression and dosage compensation on the *Artemia franciscana* Z-chromosome. Genome Biol Evol. 11(4):1033. 30865260 10.1093/gbe/evz053 PMC6456005

[evz133-B40] Itoh Y, et al 2007 Dosage compensation is less effective in birds than in mammals. J Biol. 6(1):2. 17352797 10.1186/jbiol53 PMC2373894

[evz133-B41] Jaquiéry J, et al 2013 Masculinization of the X chromosome in the pea aphid. PLoS Genet. 9(8):e1003690. 23950732 10.1371/journal.pgen.1003690 PMC3738461

[evz133-B42] Julien P, et al 2012 Mechanisms and evolutionary patterns of mammalian and avian dosage compensation. PLoS Biol. 10(5):e1001328. 22615540 10.1371/journal.pbio.1001328 PMC3352821

[evz133-B43] Kelsey AD, et al 2015 Impact of flanking chromosomal sequences on localization and silencing by the human non-coding RNA XIST. Genome Biol. 16(1):208. 26429547 10.1186/s13059-015-0774-2 PMC4591629

[evz133-B44] Kiuchi T, et al 2014 A single female-specific piRNA is the primary determiner of sex in the silkworm. Nature 509(7502):633. 24828047 10.1038/nature13315

[evz133-B45] Kramer M, et al 2015 Developmental dynamics of X-chromosome dosage compensation by the DCC and H4K20me1 in *C. elegans*. PLoS Genet. 11(12):e1005698. 26641248 10.1371/journal.pgen.1005698 PMC4671695

[evz133-B46] Kramer M, et al 2016 Correction: developmental dynamics of X-chromosome dosage compensation by the DCC and H4K20me1 in *C. elegans*. PLoS Genet. 12(2):e1005899. 26901497 10.1371/journal.pgen.1005899 PMC4764203

[evz133-B47] Langmead B, Salzberg SL. 2012 Fast gapped-read alignment with Bowtie 2. Nat Methods. 9(4):357–359. 22388286 10.1038/nmeth.1923 PMC3322381

[evz133-B48] Lau AC, Csankovszki G. 2015 Balancing up and downregulation of the *C. elegans* X chromosomes. Curr Opin Genet Dev. 31:50–56. 25966908 10.1016/j.gde.2015.04.001 PMC4470837

[evz133-B49] Lepesant JMJ, et al 2012 Chromatin structure changes around satellite repeats on the *Schistosoma mansoni* female sex chromosome suggest a possible mechanism for sex chromosome emergence. Genome Biol. 13(2):R14. 22377319 10.1186/gb-2012-13-2-r14 PMC3701142

[evz133-B50] Lin F, Xing K, Zhang J, He X. 2012 Expression reduction in mammalian X chromosome evolution refutes Ohno’s hypothesis of dosage compensation. Proc Natl Acad Sci U S A. 109(29):11752–11757. 22753487 10.1073/pnas.1201816109 PMC3406839

[evz133-B51] Loker ES, Brant SV. 2006 Diversification, dioecy and dimorphism in schistosomes. Trends Parasitol. 22(11):521–528. 16979940 10.1016/j.pt.2006.09.001

[evz133-B52] Lott SE, et al 2011 Noncanonical compensation of zygotic X transcription in early *Drosophila melanogaster* development revealed through single-embryo RNA-Seq. PLoS Biol. 9(2):e1000590. 21346796 10.1371/journal.pbio.1000590 PMC3035605

[evz133-B53] Lucchesi JC. 2018 Transcriptional modulation of entire chromosomes: dosage compensation. J Genet. 97(2):357–364. 29932054

[evz133-B54] Lucchesi JC, Kelly WG, Panning B. 2005 Chromatin remodeling in dosage compensation. Annu Rev Genet. 39(1):615–651. 16285873 10.1146/annurev.genet.39.073003.094210

[evz133-B55] Mahajan S, Bachtrog D. 2015 Sex-specific adaptation drives early sex chromosome evolution in *Drosophila*. Genome Biol Evol. 337:341–345. 10.1126/science.1225385 PMC4107656 22822149

[evz133-B56] Mank JE. 2009 The W, X, Y and Z of sex-chromosome dosage compensation. Trends Genet. 25(5):226–233. 19359064 10.1016/j.tig.2009.03.005 PMC2923031

[evz133-B57] Mank JE. 2013 Sex chromosome dosage compensation: definitely not for everyone. Trends Genet. 29(12):677–683. 23953923 10.1016/j.tig.2013.07.005

[evz133-B58] Mank JE, Ellegren H. 2009 All dosage compensation is local: gene-by-gene regulation of sex-biased expression on the chicken Z chromosome. Heredity 102(3):312–320. 18985062 10.1038/hdy.2008.116

[evz133-B59] Marks H, et al 2009 High-resolution analysis of epigenetic changes associated with X inactivation. Genome Res. 19(8):1361–1373. 19581487 10.1101/gr.092643.109 PMC2720191

[evz133-B60] Melamed E, Arnold AP. 2007 Regional differences in dosage compensation on the chicken Z chromosome. Genome Biol. 8(9):R202. 17900367 10.1186/gb-2007-8-9-r202 PMC2375040

[evz133-B61] Muyle A, et al 2018 Genomic imprinting mediates dosage compensation in a young plant XY system. Nat Plants 4(9):677. 30104649 10.1038/s41477-018-0221-y

[evz133-B62] Naurin S, Hansson B, Hasselquist D, Kim Y-H, Bensch S. 2011 The sex-biased brain: sexual dimorphism in gene expression in two species of songbirds. BMC Genomics. 12(1):37. 21235773 10.1186/1471-2164-12-37 PMC3036617

[evz133-B63] Neves RH, et al 2005 A new description of the reproductive system of *Schistosoma mansoni* (Trematoda: Schistosomatidae) analyzed by confocal laser scanning microscopy. Parasitol Res. 95(1):43–49. 15565465 10.1007/s00436-004-1241-2

[evz133-B64] Nguyen DK, Disteche CM. 2006 Dosage compensation of the active X chromosome in mammals. Nat Genet. 38(1):47. 16341221 10.1038/ng1705

[evz133-B65] Ohno S. 1967 Sex chromosomes and sex-linked genes. Springer-Verlag.

[evz133-B66] O’Neill LP, Spotswood HT, Fernando M, Turner BM. 2008 Differential loss of histone H3 isoforms mono-, di- and tri-methylated at lysine 4 during X-inactivation in female embryonic stem cells. Biol Chem. 389(4):365–370. 18225985 10.1515/BC.2008.046

[evz133-B67] Pal A, Vicoso B. 2015 The X chromosome of hemipteran insects: conservation, dosage compensation and sex-biased expression. Genome Biol Evol. 7(12):3259–3268. 26556591 10.1093/gbe/evv215 PMC4700948

[evz133-B68] Pessia E, Engelstädter J, Marais GA. 2014 The evolution of X chromosome inactivation in mammals: the demise of Ohno’s hypothesis? Cell Mol Life Sci. 71(8):1383–1394. 24173285 10.1007/s00018-013-1499-6 PMC11113734

[evz133-B69] Pessia E, Makino T, Bailly-Bechet M, McLysaght A, Marais GA. 2012 Mammalian X chromosome inactivation evolved as a dosage-compensation mechanism for dosage-sensitive genes on the X chromosome. Proc Natl Acad Sci U S A. 109(14):5346–5351. 22392987 10.1073/pnas.1116763109 PMC3325647

[evz133-B70] Picard MAL, et al 2016 Sex-biased transcriptome of *Schistosoma mansoni*: host–parasite interaction, genetic determinants and epigenetic regulators are associated with sexual differentiation. PLoS Negl Trop Dis. 10(9):e0004930. 27677173 10.1371/journal.pntd.0004930 PMC5038963

[evz133-B71] Picard MAL, et al 2018 Evolution of gene dosage on the Z-chromosome of schistosome parasites. Elife 7: pii: e35684. 10.7554/eLife.35684 PMC6089595 30044216

[evz133-B72] Portela J, et al 2010 Whole-genome in-silico subtractive hybridization (WISH)—using massive sequencing for the identification of unique and repetitive sex-specific sequences: the example of *Schistosoma mansoni*. BMC Genomics. 11(1):387. 20565937 10.1186/1471-2164-11-387 PMC3091631

[evz133-B73] Prince EG, Kirkland D, Demuth JP. 2010 Hyperexpression of the X chromosome in both sexes results in extensive female bias of X-linked genes in the flour beetle. Genome Biol. Evol. 2:336–346. 20624738 10.1093/gbe/evq024 PMC2942036

[evz133-B74] Protasio AV, et al 2012 A systematically improved high quality genome and transcriptome of the human blood fluke *Schistosoma mansoni*. PLoS Negl Trop Dis. 6(1):e1455. 22253936 10.1371/journal.pntd.0001455 PMC3254664

[evz133-B75] Quinlan AR, et al 2011 Genome sequencing of mouse induced pluripotent stem cells reveals retroelement stability and infrequent DNA rearrangement during reprogramming. Cell Stem Cell 9(4):366–373. 21982236 10.1016/j.stem.2011.07.018 PMC3975295

[evz133-B76] Rice WR. 1987 The accumulation of sexually antagonistic genes as a selective agent promoting the evolution of reduced recombination between primitive sex chromosomes. Evolution 41(4):911. 28564364 10.1111/j.1558-5646.1987.tb05864.x

[evz133-B77] Richard G, et al 2017 Dosage compensation and sex-specific epigenetic landscape of the X chromosome in the pea aphid. Epigenet Chromatin 10:30. 10.1186/s13072-017-0137-1 PMC5471693 28638443

[evz133-B78] Roquis D, et al 2015 The epigenome of *Schistosoma mansoni* provides insight about how cercariae poise transcription until infection. PLoS Negl Trop Dis. 9:e0003853. 26305466 10.1371/journal.pntd.0003853 PMC4549315

[evz133-B79] Roquis D, et al 2016 Frequency and mitotic heritability of epimutations in *Schistosoma mansoni*. Mol Ecol. 25(8):1741–1758. 26826554 10.1111/mec.13555

[evz133-B80] Roquis D, et al 2018 Histone methylation changes are required for life cycle progression in the human parasite *Schistosoma mansoni*. PLoS Pathog. 14(5):e1007066. 29782530 10.1371/journal.ppat.1007066 PMC5983875

[evz133-B81] Sangrithi MN, Turner J. 2018 Mammalian X chromosome dosage compensation: perspectives from the germ line. Bioessays 40(6):1800024. 10.1002/bies.201800024 29756331

[evz133-B82] Schultheiß R, Viitaniemi HM, Leder EH. 2015 Spatial dynamics of evolving dosage compensation in a young sex chromosome system. Genome Biol Evol. 7(2):581–590. 25618140 10.1093/gbe/evv013 PMC4350182

[evz133-B83] Shao C, et al 2014 Epigenetic modification and inheritance in sexual reversal of fish. Genome Res. 24(4):604–615. 24487721 10.1101/gr.162172.113 PMC3975060

[evz133-B84] Stenberg P, et al 2009 Buffering of segmental and chromosomal aneuploidies in *Drosophila melanogaster*. PLoS Genet. 5(5):e1000465. 19412336 10.1371/journal.pgen.1000465 PMC2668767

[evz133-B85] Trapnell C, Pachter L, Salzberg SL. 2009 TopHat: discovering splice junctions with RNA-Seq. Bioinformatics 25(9):1105–1111. 19289445 10.1093/bioinformatics/btp120 PMC2672628

[evz133-B86] Uebbing S, et al 2015 Quantitative mass spectrometry reveals partial translational regulation for dosage compensation in chicken. Mol Biol Evol. 32(10):2716–2725. 26108680 10.1093/molbev/msv147 PMC4576709

[evz133-B87] Valsecchi CIK, et al 2018 Facultative dosage compensation of developmental genes on autosomes in *Drosophila* and mouse embryonic stem cells. Nat Commun. 9(1):3626. 30194291 10.1038/s41467-018-05642-2 PMC6128902

[evz133-B88] Veitia RA. 2005 Gene dosage balance: deletions, duplications and dominance. Trends Genet. 21(1):33–35. 15680512 10.1016/j.tig.2004.11.002

[evz133-B89] Vicoso B, Bachtrog D. 2009 Progress and prospects toward our understanding of the evolution of dosage compensation. Chromosome Res. 17(5):585–602. 19626444 10.1007/s10577-009-9053-y PMC2758192

[evz133-B90] Vicoso B, Bachtrog D. 2011 Lack of global dosage compensation in *Schistosoma mansoni*, a female-heterogametic parasite. Genome Biol Evol. 3:230–235. 21317157 10.1093/gbe/evr010 PMC3068002

[evz133-B91] Vicoso B, Emerson JJ, Zektser Y, Mahajan S, Bachtrog D. 2013 Comparative sex chromosome genomics in snakes: differentiation, evolutionary strata, and lack of global dosage compensation. PLoS Biol. 11(8):e1001643. 24015111 10.1371/journal.pbio.1001643 PMC3754893

[evz133-B92] Vielle A, et al 2012 H4K20me1 contributes to downregulation of X-linked genes for *C. elegans* dosage compensation. PLoS Genet. 8(9):e1002933. 23028348 10.1371/journal.pgen.1002933 PMC3441679

[evz133-B93] Walters JR, Hardcastle TJ. 2011 Getting a full dose? Reconsidering sex chromosome dosage compensation in the silkworm, *Bombyx mori*. Genome Biol Evol. 3:491–504. 21508430 10.1093/gbe/evr036 PMC3296447

[evz133-B94] Wang B, Collins JJ, Newmark PA. 2013 Functional genomic characterization of neoblast-like stem cells in larval *Schistosoma mansoni*. Elife 2:e00768. 10.7554/eLife.00768 PMC3728622 23908765

[evz133-B95] Whitworth DJ, Pask AJ. 2016 The X factor: X chromosome dosage compensation in the evolutionarily divergent monotremes and marsupials. Semin Cell Dev Biol. 56:117–121. 26806635 10.1016/j.semcdb.2016.01.006

[evz133-B96] Wolf JB, Bryk J. 2011 General lack of global dosage compensation in ZZ/ZW systems? Broadening the perspective with RNA-Seq. BMC Genomics. 12(1):91. 21284834 10.1186/1471-2164-12-91 PMC3040151

[evz133-B97] Wright AE, Zimmer F, Harrison PW, Mank JE. 2015 Conservation of regional variation in sex-specific sex chromosome regulation. Genetics 201(2):587–598. 26245831 10.1534/genetics.115.179234 PMC4596671

[evz133-B98] Xiong Y, et al 2010 RNA sequencing shows no dosage compensation of the active X-chromosome. Nat Genet. 42(12):1043. 21102464 10.1038/ng.711

