# Supplementary material for: Dosage Compensation throughout the Schistosoma mansoni Lifecycle: Specific Chromatin Landscape of the Z Chromosome
Source: Genome Biol Evol. 2019 Jul 3;11(7):1909–22. doi: 10.1093/gbe/evz133 (PMC6628874; doi:10.1093/gbe/evz133)
Supplement: Supplementary_Material_evz133 [file supplementary_material_evz133.zip › GBE-180921_Review_SupportingInformations.pdf]

ANSWER TO REVIEWERS - SUPPORTING INFORMATIONS

Manuscript: **GBE-180921** "Dosage compensation throughout the *Schistosoma mansoni* lifecycle: specific chromatin landscape of the Z chromosome."

First referee: suggested analysis

"Can the authors test between the developmental stages to see if H3K4me3 is most enriched in cercariae females relative to immature female worms or male cercariae? Similarly, the authors report an overall loss of H3K27me3 in Z-specific genes compared to the pseudoautosomal region and chromosome 1, but this does not look that much different than the plot of immature females in Figure 3. Can the authors also test between developmental stages of this histone modification to see if there is a statistically significant under-enrichment of H3K27me3 in cercariae females relative to immature females?"

List of supporting informations answering this request:

SI.1. Comparison of H3K4me3 enrichment profiles between cercariae and immature worms.
SI.2. Comparison of H3K27me3 enrichment profiles between cercariae and immature worms.
SI.3. Comparison of H4K20me1 enrichment profiles between cercariae and immature worms
SI.4. Percentage of maximum difference in H3K4me3 (A) and H3K27me3 (B) enrichment between stages, in males.
SI.5. Percentage of maximum difference in H3K4me3 (A), H3K27me3 (B) and H4K20me1 (C) enrichment between stages, in females.
SI.6. Comparison of H3K4me3 enrichment profiles between males and females.
SI.7. Comparison of H3K27me3 enrichment profiles between males and females.
SI.8. Percentage of maximum difference in H3K4me3 (A) and H3K27me3 (B) enrichment between sexes.

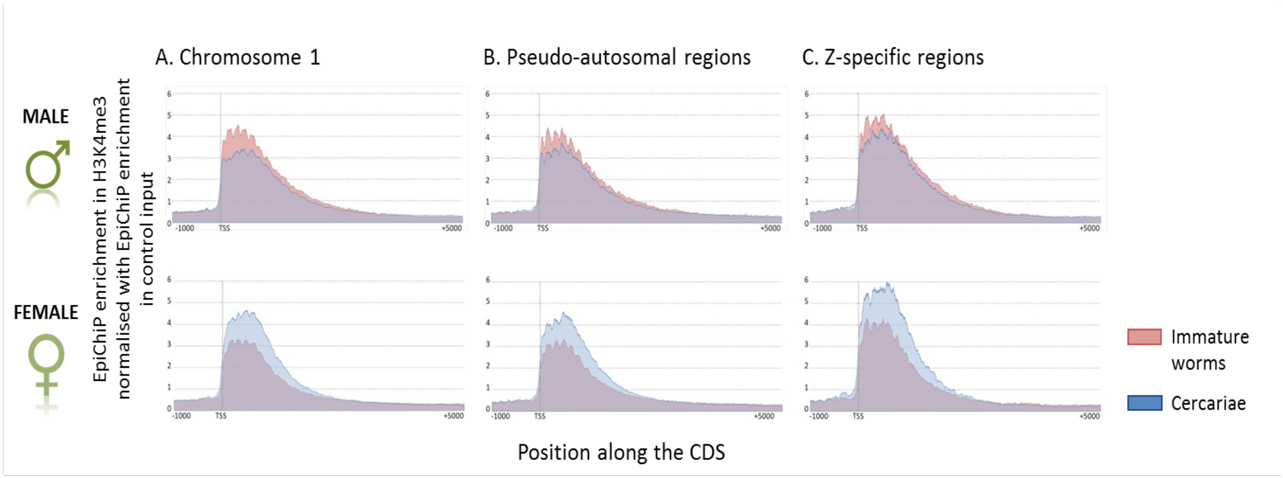

Supporting Information 1. Comparison of H3K4me3 enrichment profiles between cercariae and immature worms. X axis represents the position in bases relative to the transcriptional start site (TSS, position 0), Y axis represents the normalized average enrichment of reads obtained after a ChIP targeting **H3K4me3** on males (upper panel) and females (lower panel), either cercariae (in blue) or immature worms (in red). The EpiChIP enrichment has been calculated around the TSS for chromosome 1 **(A)**, for the pseudo-autosomal regions **(B)** and for the Z-specific regions **(C)**. This profile is the average result of each profile obtained from all CDS located in each considered region. Each profile has been normalized with the same average enrichment of reads obtained after a ChIP without antibody. All differences are statistically significant (P-value < 0.001, Kolmogorov-Smirnov two sample tests); percentages of maximum differences are shown in Supporting Information 4 for males, and Supporting Information 5 for females.

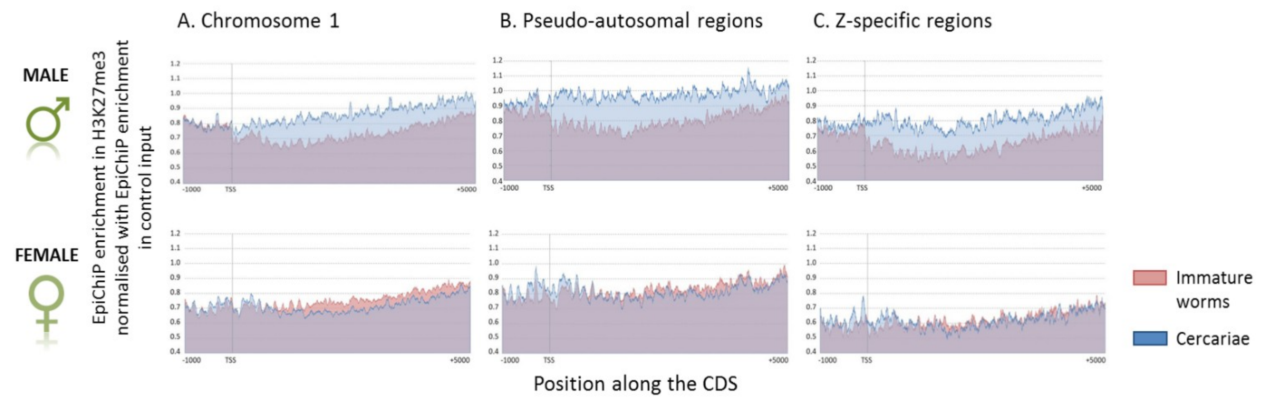

Supporting Information 2. Comparison of H3K27me3 enrichment profiles between cercariae and immature worms. X axis represents the position in bases relative to the transcriptional start site (TSS, position 0), Y axis represents the normalized average enrichment of reads obtained after a ChIP targeting H3K27me3 on males (upper panel) and females (lower panel), either cercariae (in blue) or immature worms (in red). The EpiChIP enrichment has been calculated around the TSS for chromosome 1 **(A)**, for the pseudo-autosomal regions **(B)** and for the Z-specific regions **(C)**. This profile is the average result of each profile obtained from all CDS located in each considered region. Each profile has been normalized with the same average enrichment of reads obtained after a ChIP without antibody. All differences are statistically significant (P-value < 0.001, Kolmogorov-Smirnov two sample tests); percentages of maximum differences are shown in Supporting Information 4 for males, and Supporting Information 5 for females.

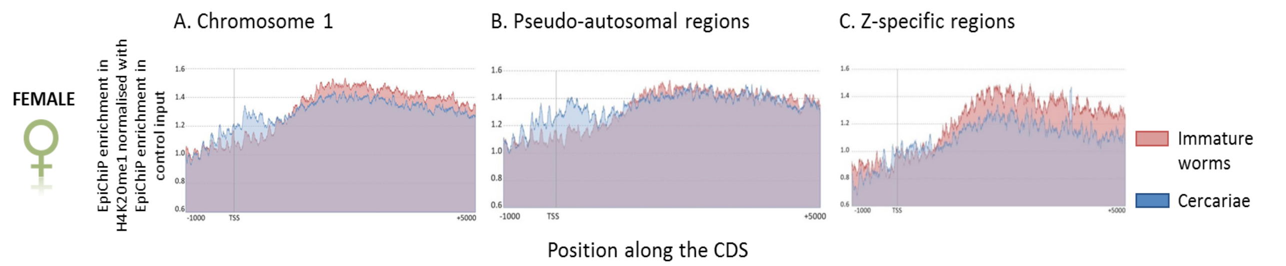

Supporting Information 3. Comparison of H4K20me1 enrichment profiles between cercariae and immature worms. X axis represents the position in bases relative to the transcriptional start site (TSS, position 0), Y axis represents the normalized average enrichment of reads obtained after a ChIP targeting H4K20me1 on female cercariae (in blue) and immature worms (in red). The EpiChIP enrichment has been calculated around the TSS for chromosome 1 (A), for the pseudo-autosomal regions (B) and for the Z-specific regions (C). This profile is the average result of each profile obtained from all CDS located in each considered region. Each profile has been normalized with the same average enrichment of reads obtained after a ChIP without antibody. All differences are statistically significant (P-value < 0.001, Kolmogorov-Smirnov two sample tests); percentages of maximum differences are shown in and Supporting Information 5.

Supporting Information 4. Percentage of maximum difference in H3K4me3 (A) and H3K27me3 (B) enrichment <u>between stages, in males.</u> Comparison of maximum differences [Cercariae / Immature adults] was performed doing a Kolmogorov-Smirnov two sample tests. All differences are statistically significant (P-value < 0.001).

	**A. H3K4me3**	**B. H3K27me3**
Chr.1	17,5%	56,3%
PAR	9,8%	87,0%
Z	11,4%	77,0%

Supporting information 5. Percentage of maximum difference in H3K4me3 (A), H3K27me3 (B) and H4K20me1 (C) enrichment between stages, in females. Comparison of maximum differences [Cercariae / Immature adults] was performed doing a Kolmogorov-Smirnov two sample tests. All differences are statistically significant (P-value < 0.001).

	**A. H3K4me3**	**B. H3K27me3**	**C. H4K20me1**
Chr.1	18,1%	25,6%	30,7%
PAR	13,5%	11,4%	20,7%
Z	21,5%	5,5%	48,5%

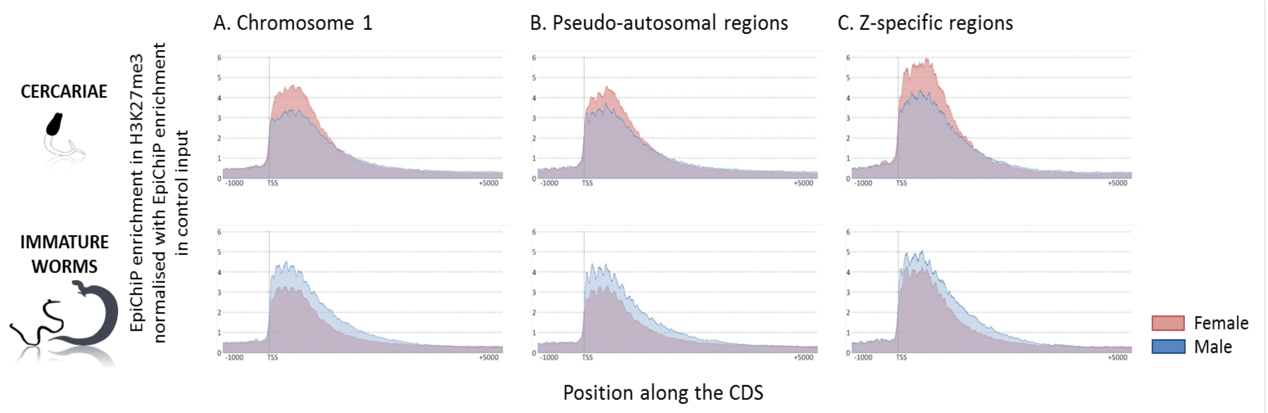

Supporting information 6. Comparison of H3K4me3 enrichment profiles between males and females. X axis represents the position in bases relative to the transcriptional start site (TSS, position 0), Y axis represents the normalized average enrichment of reads obtained after a ChIP targeting H3K4me3 on cercariae (upper panel) and immature worms (lower panel), either males (in blue) or females (in red). The EpiChIP enrichment has been calculated around the TSS for chromosome 1 (A), for the pseudo-autosomal regions (B) and for the Z-specific regions (C). This profile is the average result of each profile obtained from all CDS located in each considered region. Each profile has been normalized with the same average enrichment of reads obtained after a ChIP without antibody. All differences are statistically significant (P-value < 0.001, Kolmogorov-Smirnov two sample tests); percentages of maximum differences are shown in Supporting Information 8.

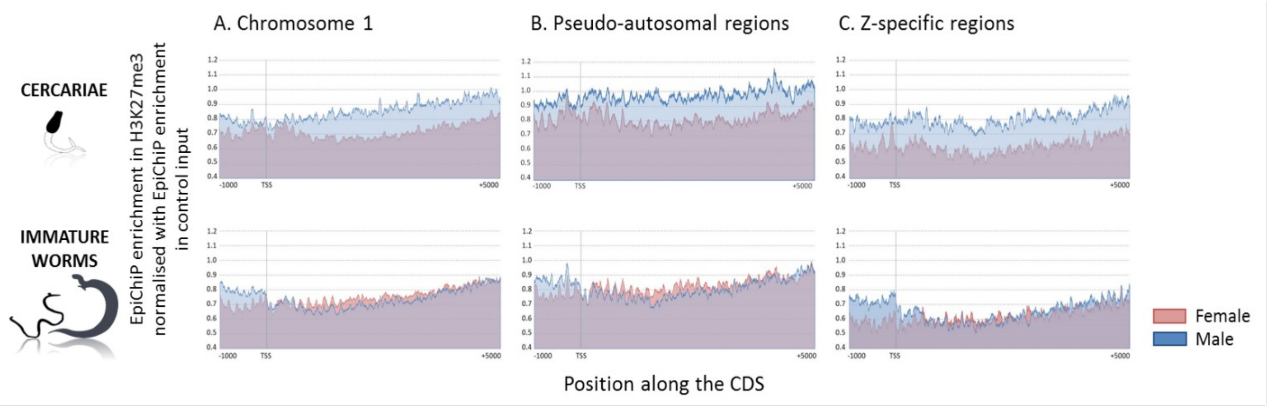

Supporting information 7. Comparison of H3K27me3 enrichment profiles between males and females. X axis represents the position in bases relative to the transcriptional start site (TSS, position 0), Y axis represents the normalized average enrichment of reads obtained after a ChIP targeting H3K27me3 on cercariae (upper panel) and immature worms (lower panel), either males (in blue) or females (in red). The EpiChIP enrichment has been calculated around the TSS for chromosome 1 (A), for the pseudo-autosomal

regions (B) and for the Z-specific regions (C). This profile is the average result of each profile obtained from all CDS located in each considered region. Each profile has been normalized with the same average enrichment of reads obtained after a ChIP without antibody. All differences are statistically significant (P-value < 0.001, Kolmogorov-Smirnov two sample tests); percentages of maximum differences are shown in Supporting Information 8.

Supporting information 8. Percentage of maximum difference in H3K4me3 (A) and H3K27me3 (B) enrichment between sexes. Comparison of maximum differences [Female / Male] was performed doing a Kolmogorov-Smirnov two sample tests. All differences are statistically significant (P-value < 0.001).

**A. H3K4me3**	Cercariae	Immature worms
Chr.1	19.9%	17.1%
PAR	14.7%	12.6%
Z	21.8%	11.2%

**B. H3K27me3**	Cercariae	Immature worms
Chr.1	76.5%	10.7%
PAR	88.7%	14.6%
Z	92.4%	26.7%

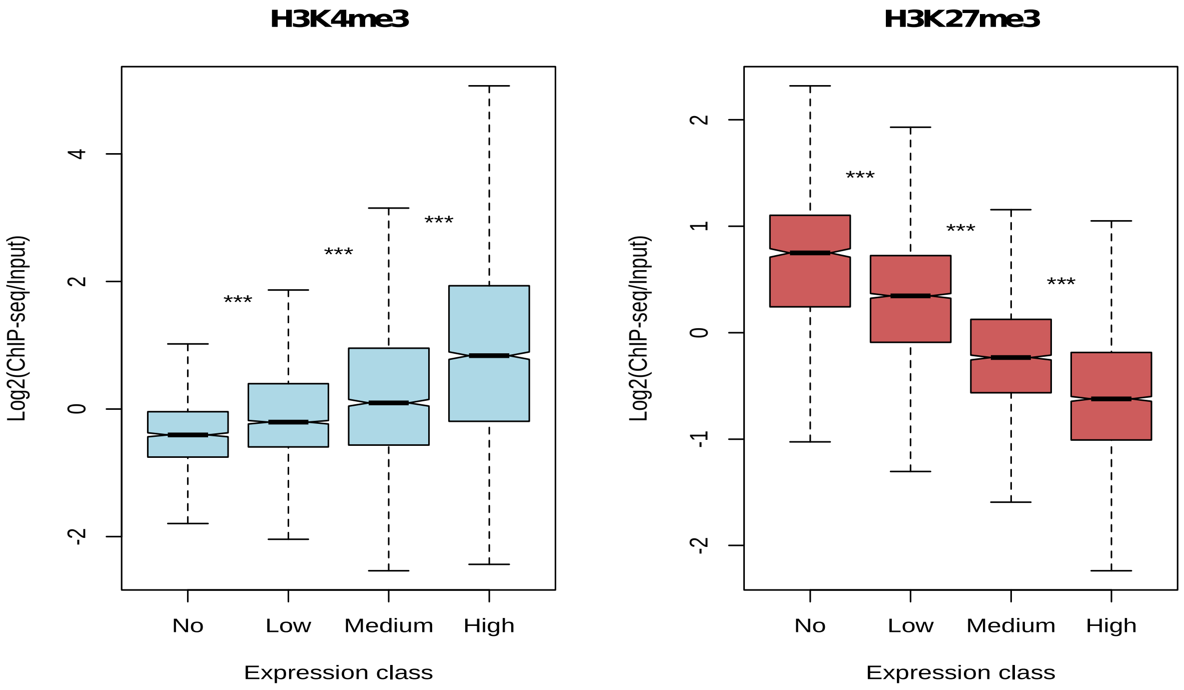

Supporting information 9. Log2 ratio of TPM obtained from ChIP-seq and input datasets mapped to *S. mansoni* transcripts for genes with different levels of expression. The left hand panel shows enrichment patterns for H3K4me3 while the right hand panel shows enrichment patterns for H3K27me3. *** denote p-values<2.2e-16 with Wilcoxon tests.

[Brief summary of how these plots were produced: *S. mansoni* transcripts were obtained from parasite.wormbase.org. The fastq files for samples SRR3225253 (female input), SRR3225252 (female H3K4me3 ChIP-seq), SRR3225239 (female H3K27me3 ChIP-seq) and SRR3223448 (female RNA-seq) were mapped to the transcripts and Transcripts Per Million (TPM) values obtained for each sample using Kallisto. The TPMs of all the transcripts corresponding to each gene were summed to obtain a single TPM per gene. Genes were separated into expression classes based on the RNA-seq TPM: no expression (TPM<0.5), medium expression (20<TPM<50) and high expression (TPM>50).]
