# Supplementary material for: Dosage Compensation throughout the Schistosoma mansoni Lifecycle: Specific Chromatin Landscape of the Z Chromosome
Source: Genome Biol Evol. 2019 Jul 3;11(7):1909–22. doi: 10.1093/gbe/evz133 (PMC6628874; doi:10.1093/gbe/evz133)
Supplement: Supplementary_Material_evz133 [file supplementary_material_evz133.zip › GBE-180921_SupplementaryFigures.pdf]

SUPPLEMENTARY DATA

SUPPLEMENTARY FIGURES

Supplementary Figure 1. Female-to-male expression ratio according to the location along the Z-chromosome, and the developmental stages (including strong sex-biased genes). P3

Supplementary Figure 2. Female-to-male expression ratio according to the location along the Z-chromosome, and the developmental stages (excluding strong sex-biased genes). P4

Supplementary Figure 3. Gene expression pattern according to sex and Z-chromosome region. P5

Supplementary Figure 4. Gene expression clustering of the three studied developmental stages, according to their genomic location. P6

Supplementary Figure 5. Female-to-male ratio of expression for chromosome 1 throughout *S. mansoni* development. P7

Supplementary Figure 6. Epichip profiles of H3K4me3 showing standard error at each position. P7

Supplementary Figure 7. Epichip profiles of H3K27me3 showing standard error at each position. P8

Supplementary Figure 8. Epichip profiles of H3K20me1 showing standard error at each position. P8

Supplementary Figure 9. Correspondance between the three Z-specific regions Z1, Z2, Z3 and evolutionary strata previously described. P10

SUPPLEMENTARY TABLES

Supplementary Table 1. Number of analysed genes after filtering for minimum gene expression level. P2

Supplementary Table 2. Ratio of gene expression between the three Z-specific and the pseudo-autosomal regions, according to developmental stage and sex. P4

Supplementary Table 3. Percentage of maximum difference in H3K4me3 enrichment between genomic location. P9

Supplementary Table 4. Percentage of maximum difference in H3K27me3 enrichment between genomic location. P9

Supplementary Table 5. Percentage of maximum difference in H4K20me1 enrichment between genomic location. P9

Supplementary Table 1. Number of analysed genes after filtering for minimum gene expression level.

	A. CERCARIAE				B. SCHISTOSOMULA				C. IMMATURE WORMS			
	**n Total**	**n Aut.**	**n PAR**	**n Z**	**n Total**	**n Aut.**	**n PAR**	**n Z**	**n Total**	**n Aut.**	**n PAR**	**n Z**
**RPKM > 0**	10,005	5,371	981	734	10,773	5,715	1,042	776	9,881	5,309	971	732
**RPKM > 0 - SEX-BIAS FILTERED**	5,250	2,830	548	369	8,934	4,852	888	553	7,982	4,379	788	519
**RPKM > 1**	5,870	3,145	582	474	6,253	3,354	618	492	6,256	3,361	608	497
**RPKM > 1 - SEX-BIAS FILTERED**	3,741	2,016	393	285	5,636	3,125	575	360	5,657	3,096	561	384
**RPKM > 5**	3,052	1,612	301	235	3,403	1,815	343	255	3,450	1,845	335	260
**RPKM > 5 - SEX-BIAS FILTERED**	2,049	1,081	215	153	3,106	1,719	320	187	3,207	1,735	321	214

Supplementary Table 2. Ratio of gene expression between the three Z-specific and the pseudo-autosomal regions, according to developmental stage and sex. Female-to-male expression ratio of Z-specific genes compared to pseudo-autosomal regions **(A)** are shown for cercariae, schistosomula and immature worms. The Z-to-autosome ratio are shown for females **(B)** and males **(C)** for the three same stages. Only genes with expression RPKM>1 are considered, and strongly sex-biased genes (fold change > 2) were excluded. The level of significance for each comparison is indicated by the asterisks (Wilcoxon test): *P-value<0.05, **P-value<0.001, ***P-value<0.0001, N.S.: non-significant difference.

		A) FEMALE: MALE			B) FEMALES			C) MALES		
		**Z1:PAR**	**Z2:PAR**	**Z3:PAR**	**Z1:PAR**	**Z2:PAR**	**Z3:PAR**	**Z1:PAR**	**Z2:PAR**	**Z3:PAR**
**CERCARIAE**	Ratio	0.76	0.88	0.81	1.00	1.13	0.99	1.00	1.14	0.97
	P-value	***	N.S.	*	N.S.	N.S.	N.S.	N.S.	N.S.	N.S.
**SCHISTOSOMULA**	Ratio	0.72	0.71	0.74	0.68	0.70	1.00	0.93	1.03	1.25
	P-value	***	***	***	*	N.S.	N.S.	N.S.	N.S.	*
**ADULTS**	Ratio	0.64	0.62	0.62	0.81	0.81	0.94	1.19	1.09	1.42
	P-value	***	***	***	N.S.	N.S.	N.S.	N.S.	N.S.	*

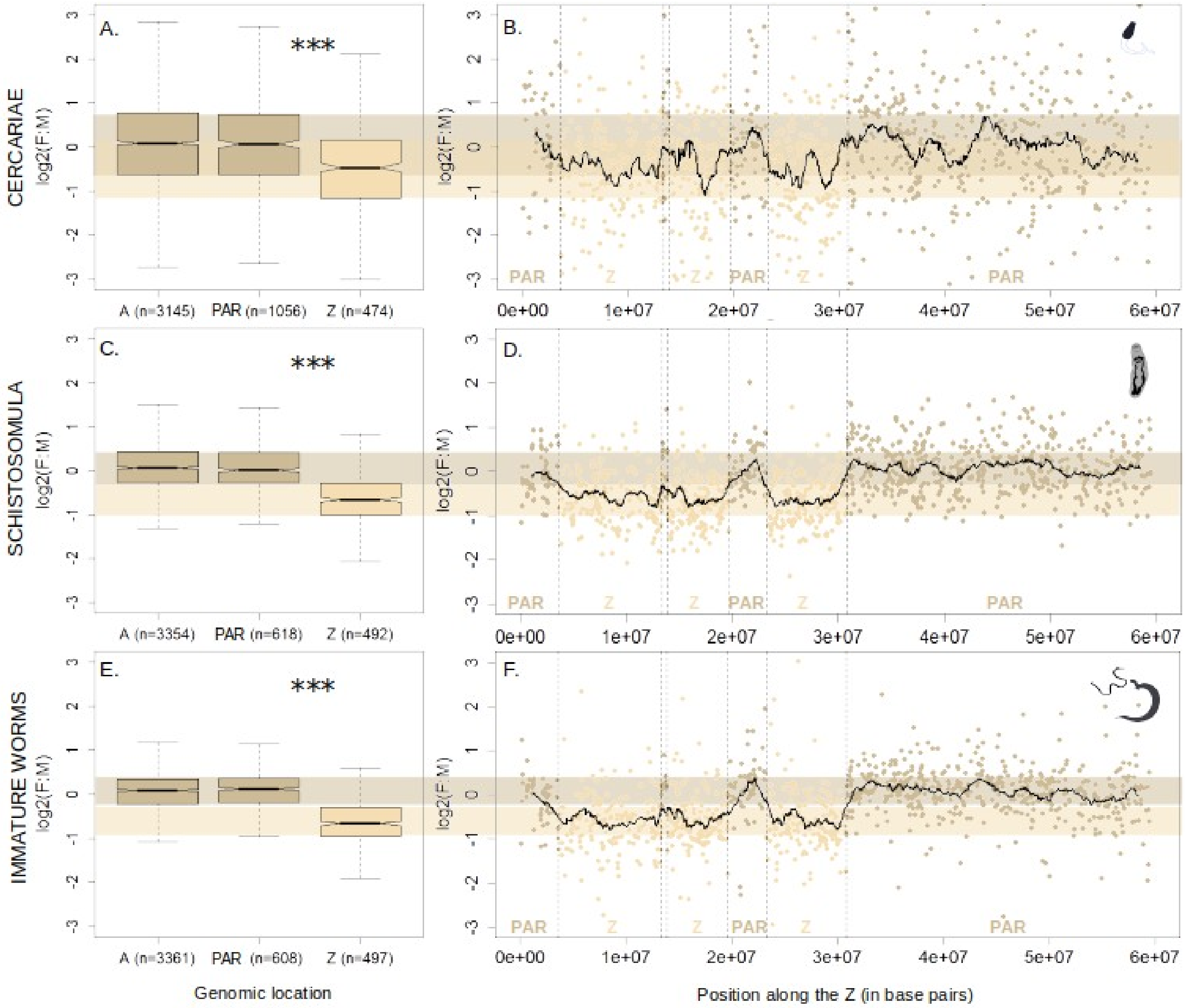

Supplementary Figure 1. Female-to-male expression ratio according to the location along the Z-chromosome, and the developmental stages (including strong sex-biased genes). The female-to-male expression ratio (F:M) is represented for cercariae **(A, B)**, schistosomula **(C, D)**, and immature worms **(E, F)**. Only genes with expression RPKM>1 are shown, independently of the F:M fold change (no filter on sex-bias). For each stage, the F:M ratio is shown depending on the genomic location **(A, C, E)**: autosomes (A, dark beige), pseudo-autosomal regions of the sex chromosomes (PAR, dark beige) or Z-specific regions (Z, light beige). Values of female-to-male ratio comparisons are: in cercariae, PAR:A=0.99$^{N.S.}$, Z:A=0.68***, Z:PAR=0.69*** ; in schistosomula, PAR:A=0.97$^{N.S.}$, Z:A=0.61***, Z:PAR=0.63***; in adults, PAR:A=1.03$^{N.S.}$, Z:A=0.59***, Z:PAR=0.58*** (****P-value*<0.0001, *N.S.* = non-significant). On the figure, asterisks show the level of significance of Z-to-PAR comparison (Wilcoxon rank sum test with continuity correction). The female-to-male expression of genes located on sex-chromosomes are plotted depending on their position along the Z-chromosome **(B, D, F)**, and dot colours indicate if they belong to pseudo-autosomal (PAR, dark beige) or Z-specific regions (Z, light beige). The thick black line represents female-to-male expression ratio by sliding window of 30 genes. Vertical dashed lines limit the Z-specific regions as defined in the version 5.2 of the genome.

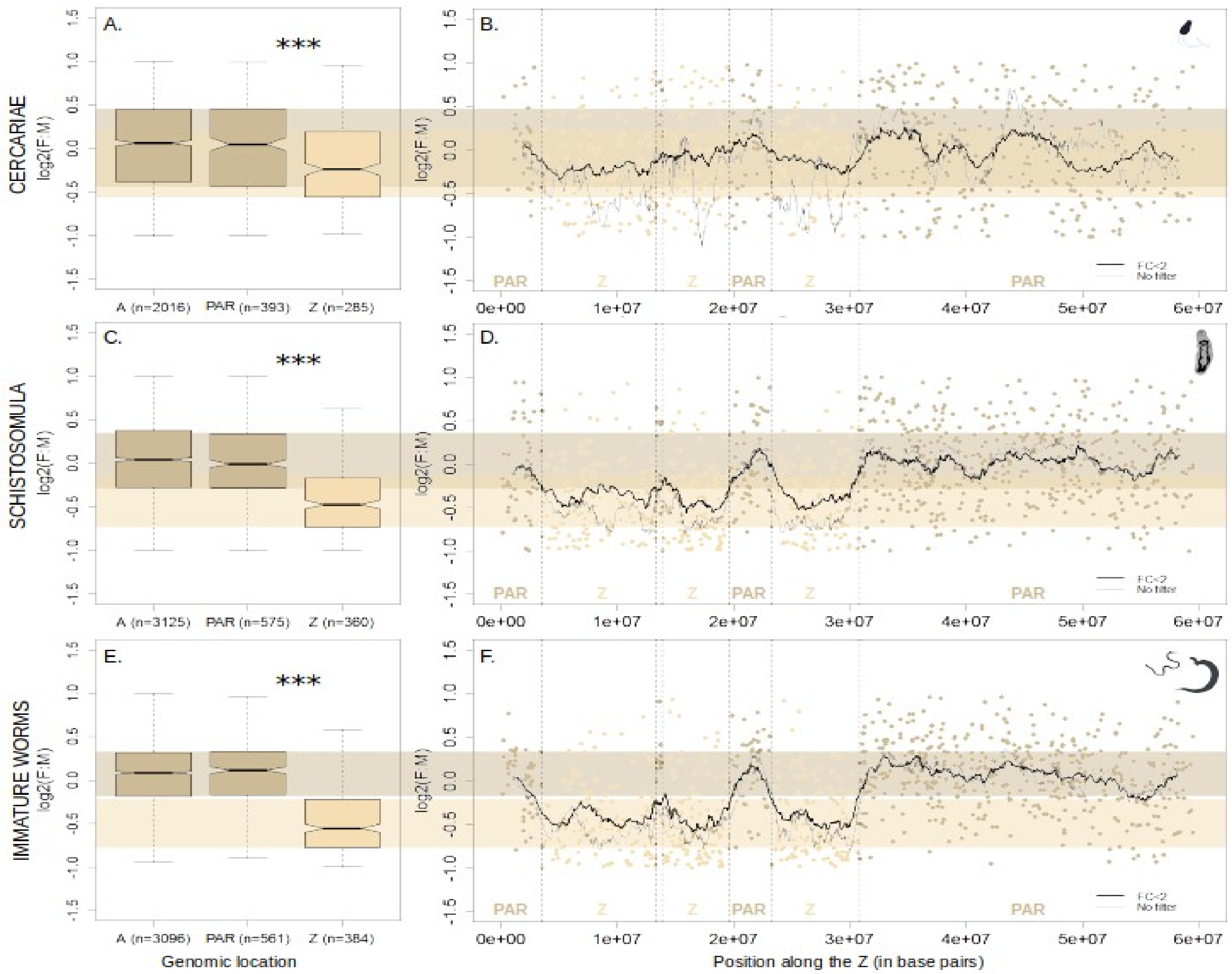

Supplementary Figure 2. Female-to-male expression ratio according to the location along the Z-chromosome, and the developmental stages (excluding strong sex-biased genes). The female-to-male expression ratio (F:M) is represented for cercariae **(A, B)**, schistosomula **(C, D)**, and immature worms **(E, F)**. Only genes with expression RPKM>1 are shown, and strongly sex-biased genes (fold change > 2) were excluded. For each stage, the F:M ratio is shown depending on the genomic location **(A, C, E)**: autosomes (A, dark beige), pseudo-autosomal regions of the sex chromosomes (PAR, dark beige) or Z-specific regions (Z, light beige). Values of female-to-male ratio comparisons are: in cercariae, PAR:A=0.99$^{N.S.}$, Z:A=0.81***, Z:PAR=0.82*** ; in schistosomula, PAR:A=0.97$^{N.S.}$, Z:A=0.70***, Z:PAR=0.72***; in adults, PAR:A=1.02$^{N.S.}$, Z:A=0.64***, Z:PAR=0.63*** (****P-value*<0.0001, *N.S.* = non-significant). On the figure, asterisks show the level of significance of Z-to-PAR comparison (Wilcoxon rank sum test with continuity correction). The female-to-male expression of genes located on sex-chromosomes are plotted depending on their position along the Z-chromosome **(B, D, F)**, and dot colours indicate if they belong to pseudo-autosomal (PAR, dark beige) or Z-specific regions (Z, light beige). The thick black line represents female-to-male expression ratio by sliding window of 30 genes, after excluding the strongly sex-biased genes, while the thin grey line indicates the same ratio including those biased genes. Vertical dashed lines limit the Z-specific regions as defined in the version 5.2 of the genome.

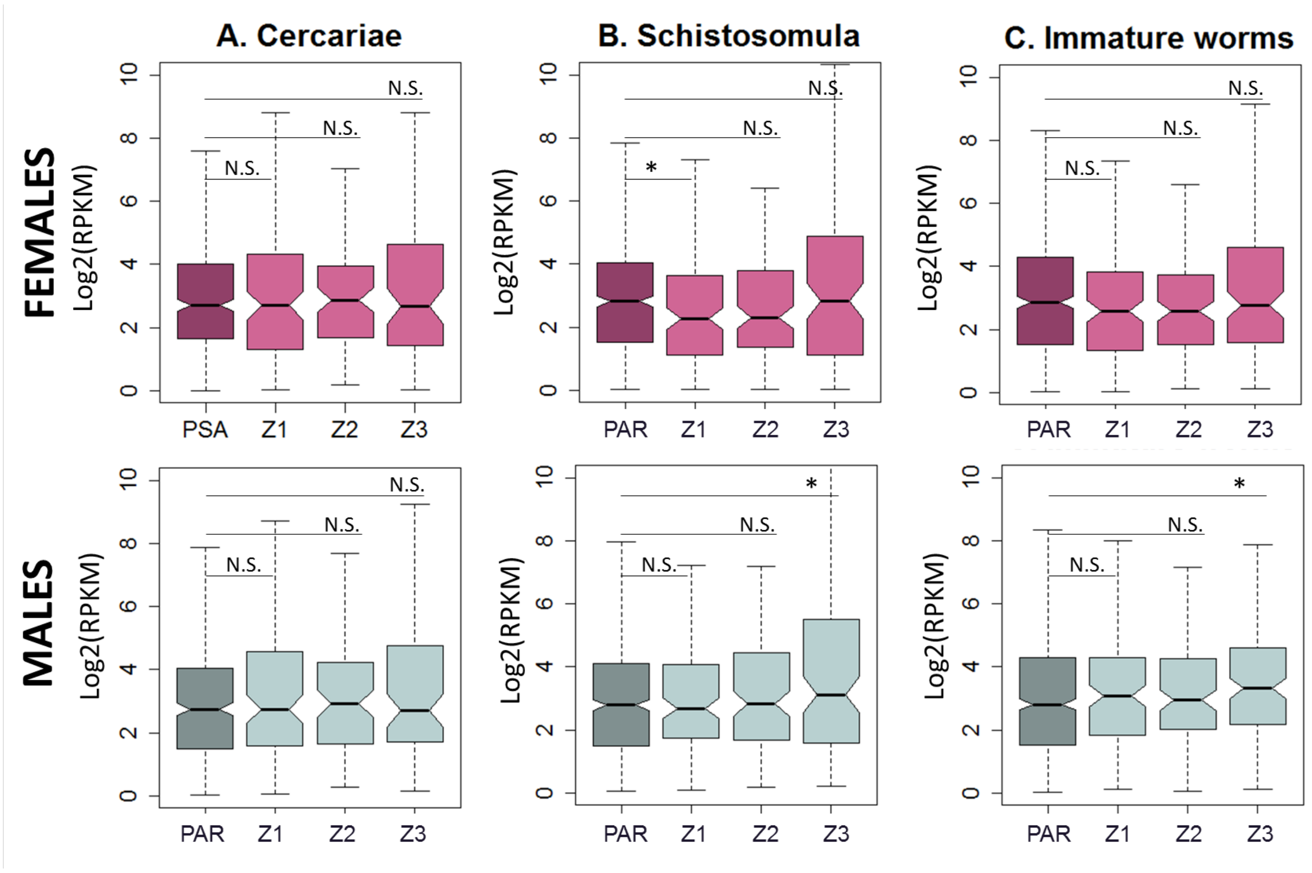

Supplementary Figure 3. Gene expression pattern according to sex and Z-chromosome region. Expression of pseudo-autosomal genes ("PAR", dark shade) and the three Z-specific regions ("Z1", "Z2" and "Z3", light shade) are represented within females (in pink) and females (in blue), for cercariae **(A)**, schistosomula **(B)** and immature worms **(C)**. Only genes with expression RPKM>1 and a sex-bias fold change <2 are shown. Asterisks show the level of significance for each comparison (Wilcoxon test): *P-value<0.05, N.S = non-significant differences. The corresponding ratios are detailed in supplementary table 2.

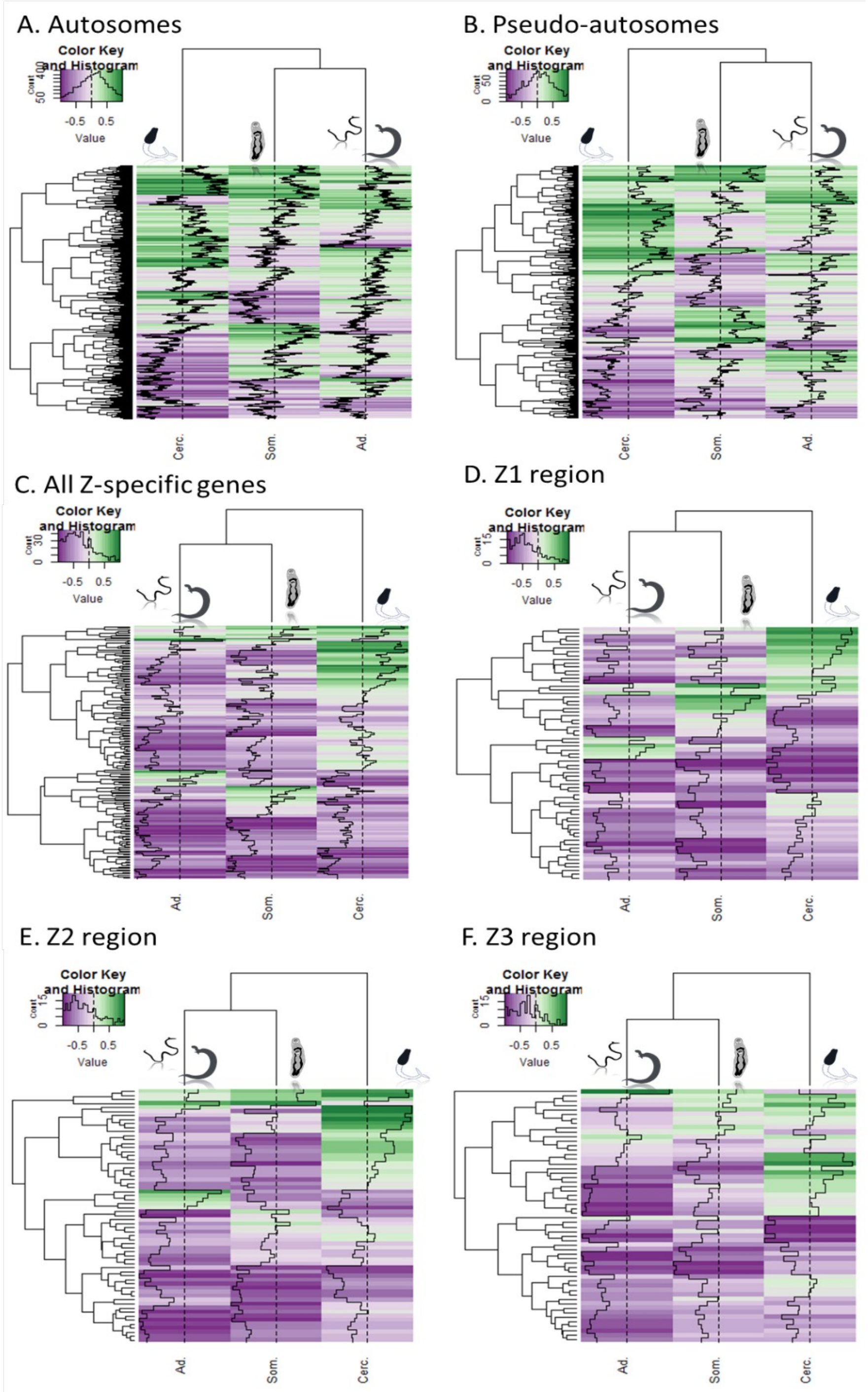

Supplementary Figure 4. Gene expression clustering of the three studied developmental stages, according to their genomic location. Female-to-male ratio is represented in logarithmic scale for autosomes **(A)**, pseudo-autosomes **(B)** and Z-specific genes **(C)**. The Z-specific genes are also represented depending on their position on the Z1 **(D)**, Z2 **(E)** or Z3 **(F)** regions as defined in the version 5.2 of the genome. Only genes with expression RPKM>1 and a sex-bias fold change <2 are shown. Female-biases are represented in green (log2>0) and male-biases in purple (log2<0). Light colours are the closest to zero and correspond to equalized expression between sexes. Dashed line corresponds to log2(F:M)=0, and the continuous line represent the F:M ratio, negative on the left (intensity of male-bias), positive on the right (intensity of female-bias).

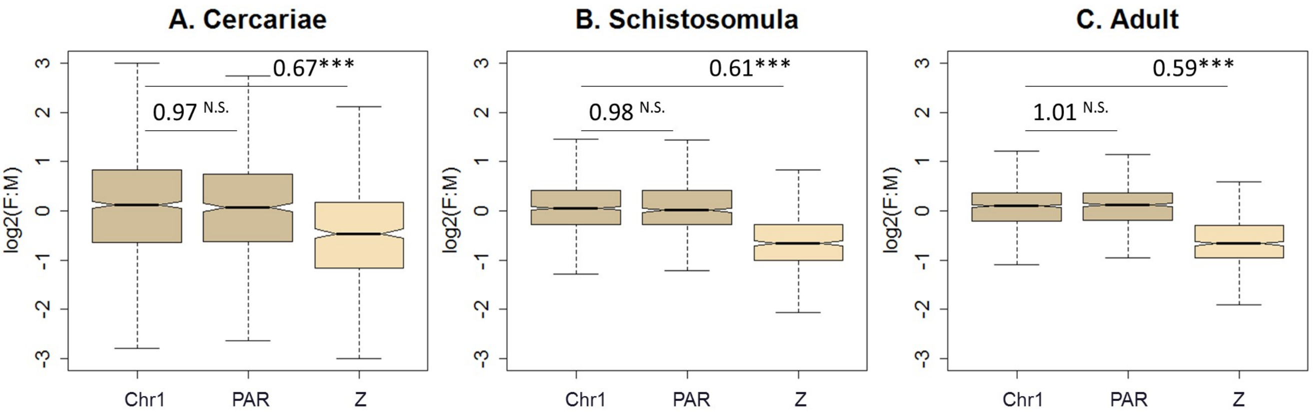

Supplementary Figure 5. Female-to-male ratio of expression for chromosome 1 throughout *S. mansoni* development. Female-to-male ratio of expression is represented for chromosome 1 (“Chr1”), pseudo-autosomal genes (PAR) and Z-specific genes (Z). Only genes with expression RPKM>1. No filter on sex-bias was applied. Ratio are indicated on the plots and level of significance for each comparison is indicated by the asterisks (Wilcoxon test): ***P-value<0.0001, N.S.: non-significant difference.

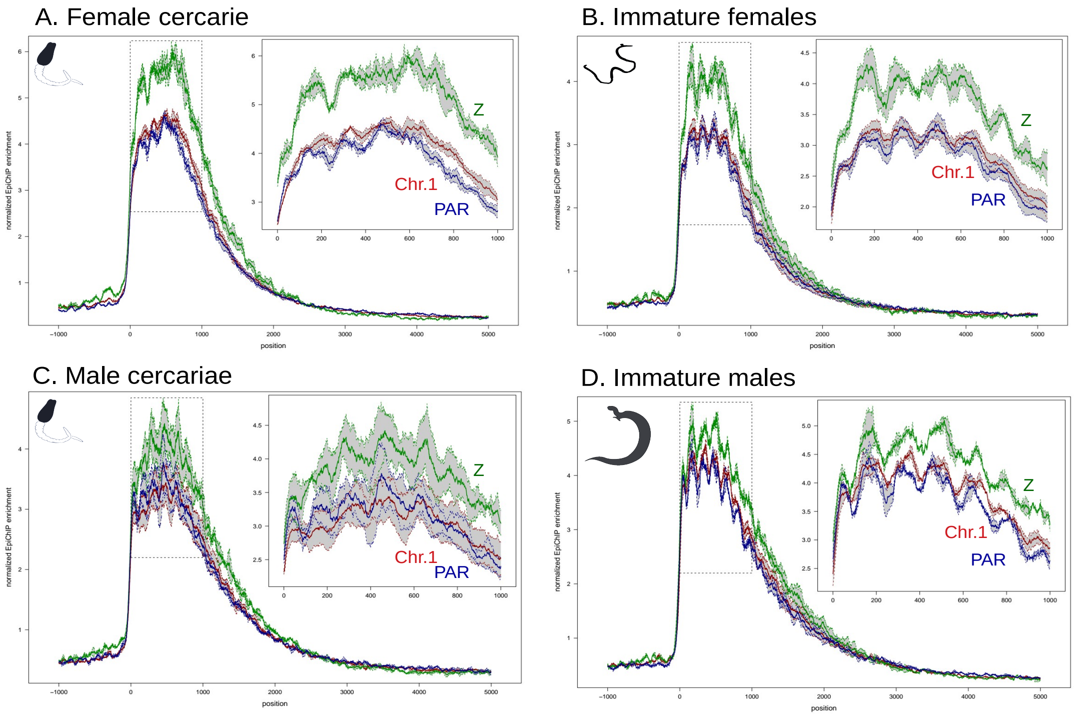

Supplementary Figure 6. Epichip profiles of H3K4me3 showing standard error at each position. To quantify the confidence level of the profiles shown in Figure 3, we calculated the standard error of the profiles at each position. Histone enrichment in H3K4me3 are represented along the gene for female cercariae and immature worms **(A and B)**, and for male cercariae and immatures worms **(C and D)**, as well as for each genomic location (“Chr.1”: chromosome 1, in red; “PAR” : pseudo-autosomal genes, in blue; “Z” : Z-specific genes, in green).

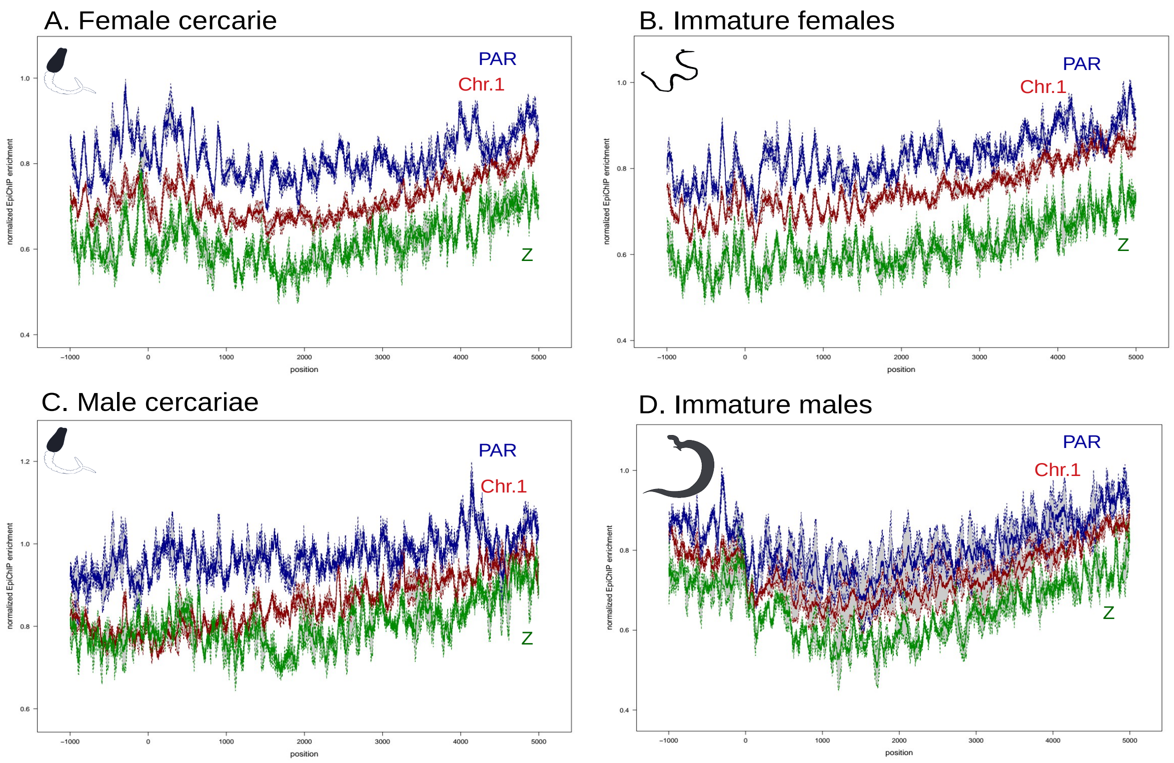

Supplementary Figure 7. Epichip profiles of H3K27me3 showing standard error at each position. To quantify the confidence level of the profiles shown in Figure 3, we calculated the standard error of the profiles at each position. Histone enrichment in H3K27me3 are represented along the gene for female cercariae and immature worms (A and B), and for male cercariae and immatures worms (C and D), as well as for each genomic location ("Chr.1": chromosome 1, in red; "PAR" : pseudo-autosomal genes, in blue; "Z" : Z-specific genes, in green).

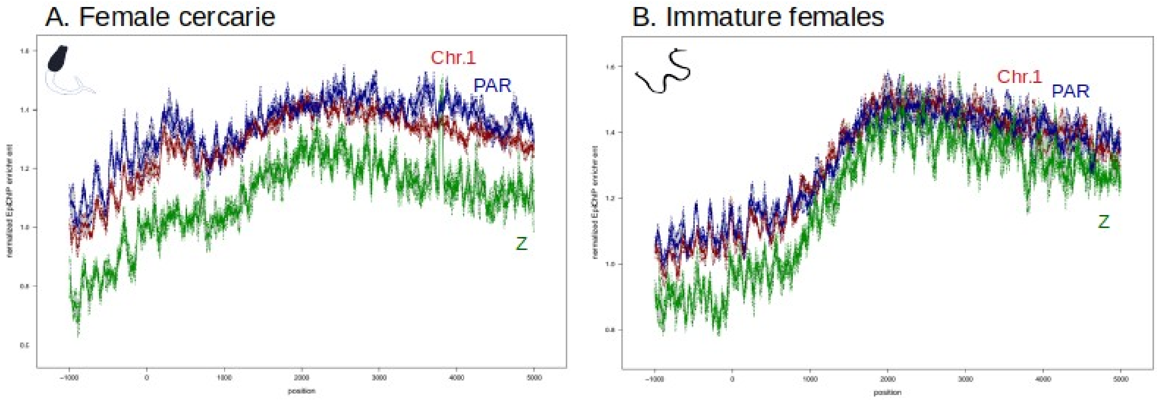

Supplementary Figure 8. Epichip profiles of H4K20me1 showing standard error at each position. Histone enrichment in H4K20me1 is represented along the gene for females, in cercariae **(A)** and immature worms **(B).** Genomic locations are shown separately ("Chr.1": chromosome 1, in red; "PAR" : pseudo-autosomal genes, in blue; "Z" : Z-specific genes, in green). The EpiChIP enrichment has been calculated around the TSS. Each profile has been normalized with the same average enrichment of reads obtained after a Chromatin Immuno-Precipitation without antibody. The experiment was performed in duplicates.

Supplementary Table 3. Percentage of maximum difference in H3K4me3 enrichment between genomic location. Comparison of maximum differences was performed doing a Kolmogorov-Smirnov two sample tests. All differences are statistically significant (P-value < 0.001).

A. Female cercariae

	Chr1	PAR	Z
Chr1			
PAR	8,2%		
Z	17,7%	15,0%	

B. Immature female worms

	Chr1	PAR	Z
Chr1			
PAR	9,7%		
Z	14,1%	13,7%	

C. Male cercariae

	Chr1	PAR	Z
Chr1			
PAR	6,9%		
Z	17,2%	16,7%	

D. Immature male worms

	Chr1	PAR	Z
Chr1			
PAR	10,6%		
Z	8,3%	11,5%	

Supplementary Table 4. Percentage of maximum difference in H3K27me3 enrichment between genomic location. Comparison of maximum differences was performed doing a Kolmogorov-Smirnov two sample tests. All differences are statistically significant (P-value < 0.001).

A. Female cercariae

	Chr1	PAR	Z
Chr1			
PAR	69,0%		
Z	73,3%	96,2%	

B. Immature female worms

	Chr1	PAR	Z
Chr1			
PAR	53,0%		
Z	75,5%	95,7%	

C. Male cercariae

	Chr1	PAR	Z
Chr1			
PAR	70,2%		
Z	32,7%	91,2%	

D. Immature male worms

	Chr1	PAR	Z
Chr1			
PAR	39,7%		
Z	43,5%	75,0%	

Supplementary Table 5. Percentage of maximum difference in H4K20me1 enrichment between genomic location. Comparison of maximum differences was performed doing a Kolmogorov-Smirnov two sample tests. All differences are statistically significant (P-value < 0.001).

A. Female cercariae

	Chr1	PAR	Z
Chr1			
PAR	26,2%		
Z	63,3%	75,2%	

B. Immature female worms

	Chr1	PAR	Z
Chr1			
PAR	9,1%		
Z	30,0%	30,7%	

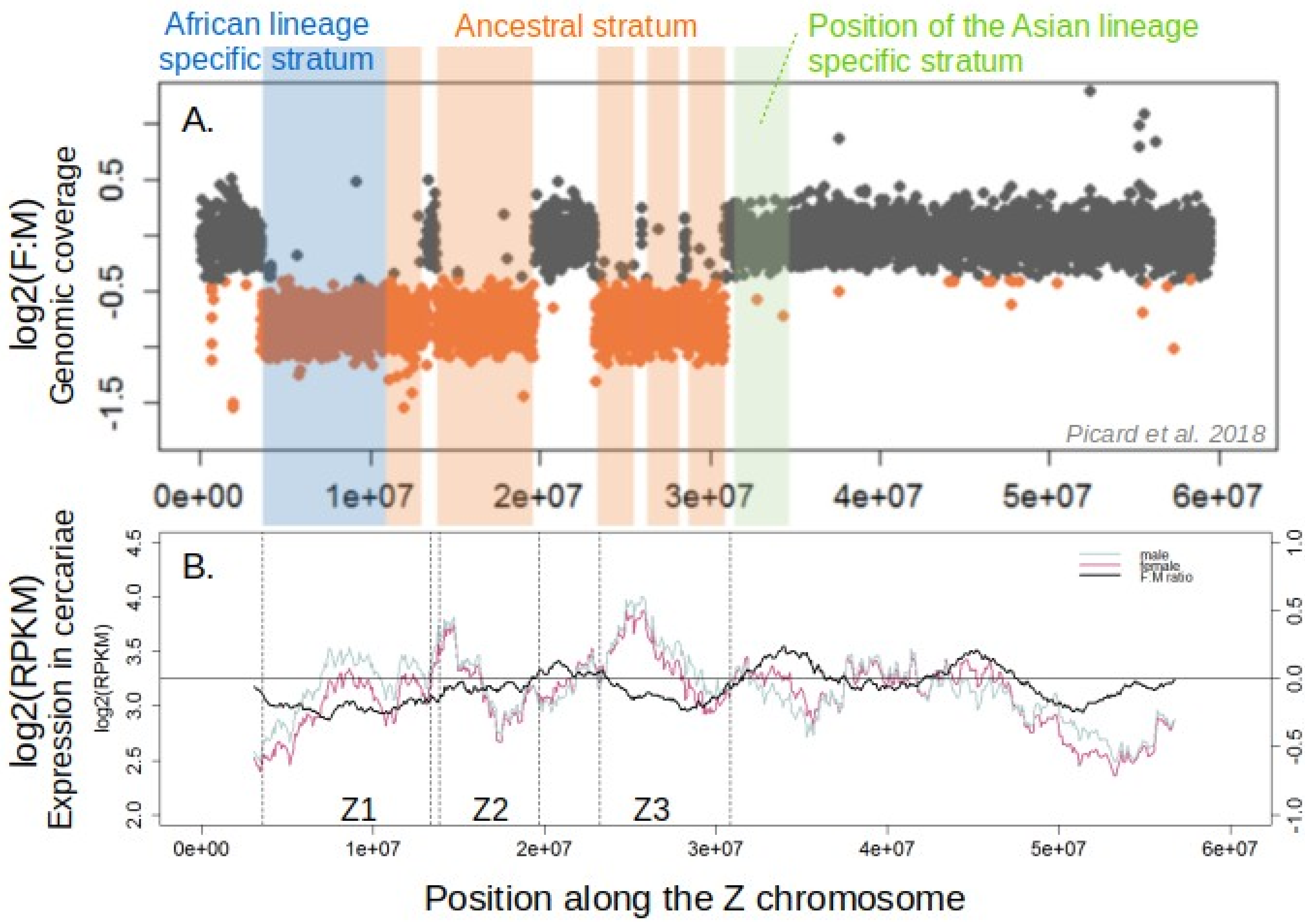

Supplementary Figure 9. Correspondance between the three Z-specific regions Z1, Z2, Z3 and evolutionary strata previously described. Two Z-specific evolutionary strata have been described in *S. mansoni* species **(A)**: one ancestral, shared by Asian and African lineages (in orange) and one shared only by the African phylum (in blue) (Picard *et al.* 2018). They are characterized by a female-to-male ratio of genomic coverage equal to 0.5. A third Z-specific stratum was described as specific of the Asian lineage (in green), but it correspond to a pseudoautosomal region in our model species *S. mansoni* (and displays a female-to-male ratio of genomic coverage equal to 1). The three Z-specific regions described in the version 5.2 of the genome **(B,** extracted from Fig. 2**)** (Protasio *et al.* 2012) , correspond to the African stratum for Z1 (main part), and to the ancestral stratum for Z2 and Z3.
