# Supplementary material for: Dosage Compensation throughout the Schistosoma mansoni Lifecycle: Specific Chromatin Landscape of the Z Chromosome
Source: Genome Biol Evol. 2019 Jul 3;11(7):1909–22. doi: 10.1093/gbe/evz133 (PMC6628874; doi:10.1093/gbe/evz133)
Supplement: Supplementary_Material_evz133 [file supplementary_material_evz133.zip › GBE-180921_SupplementaryMethods1.pdf]

```
# DESCRIPTION OF R-SCRIPT:

#A.   RAW DATA PROCESSING
    #A.1. CORRELATION ANALYSIS ON RAW DATA
    #A.2. OVERALL EXPRESSION PLOTS BEFORE LOESS NORMALIZATION
    #A.3. LOESS NORMALIZATION
    #A.4. OVERALL EXPRESSION PLOTS AFTER LOESS NORMALIZATION
    #A.5. CORRELATION ANALYSIS ON NORMALIZED DATA
#B. EXPRESSION ANALYSIS
  #B.1. AVERAGE BIOLOGICAL REPLICATES & CALCULATE RATIOS
  #B.2. FILTERS ON MINIMUM OF EXPRESSION LEVEL
  #B.3. SUBSET TABLES
  #B.4. PLOTS AND STATS
    #B.4.1. Comparative analysis per sex (FIGURE 1)
    #B.4.2. Comparative analysis per stage (SUPPLEMENTARY FIGURES 1 & 2, left
panel ; SUPPLEMENTARY FIGURE 5)
    #B.4.3. F:M ratio of expression along the Z, per stage (SUPPLEMENTARY
FIGURES 1 & 2, RIGTH PANEL)
    #B.4.4. Expression along the Z, per sex and stage (FIGURE 2)
    #B.4.5. Boxplot of F:M ratio depending of the Z specific region
(SUPPLEMENTARY FIGURE 3)
#B.5. HEATMAPS



#########################
#A. RAW DATA PROCESSING #
#########################

#Open data table
table1 <- read.table("~/SupplementaryData4.txt",fill=TRUE,header=TRUE) # RPKM
table attached as the Supplementary Data 4 /!\ It should be in a .txt format
head(table1) #16 columns
nrow(table1) #10787 rows

## A.1. CORRELATION ANALYSIS ON RAW DATA

library(gplots)

#Cercariae - correlation
Table_cor_Cerc <- (subset(table1, select= c("Male_Cercariae_1_RPKM",
"Male_Cercariae_2_RPKM", "Female_Cercariae_1_RPKM", "Female_Cercariae_2_RPKM")))
colors_cor_Cerc<- c("gray","gray","black","black")
heatmap.2(cor(Table_cor_Cerc,method="pearson"),col=colorRampPalette(c("blue","wh
ite","red","firebrick4"))(15),ColSideColors =
colors_cor_Cerc,scale="none",symm=T,margins=c(15,15),key=T,trace="none")
legend("topright",legend=c("Male","Female"),col=c("gray","black"),lty=1,lwd=10)

#Schistosomula - correlation
Table_cor_Som <- (subset(table1, select= c("Male_Schistosomula_1_RPKM",
"Male_Schistosomula_2_RPKM", "Female_Schistosomula_1_RPKM",
"Female_Schistosomula_2_RPKM")))
colors_cor_Som<- c("gray","gray","black","black")
heatmap.2(cor(Table_cor_Som,method="pearson"),col=colorRampPalette(c("blue","whi
te","red","firebrick4"))(15),ColSideColors =
colors_cor_Som,scale="none",symm=T,margins=c(15,15),key=T,trace="none")
legend("topright",legend=c("Male","Female"),col=c("gray","black"),lty=1,lwd=10)

#Adults - correlation
Table_cor_Ad <- (subset(table1, select= c("Male_Adult_1_RPKM",
"Male_Adult_2_RPKM", "Female_Adult_1_RPKM", "Female_Adult_2_RPKM")))
colors_cor_Ad<- c("gray","gray","black","black")
heatmap.2(cor(Table_cor_Ad,method="pearson"),col=colorRampPalette(c("blue","whit
e","red","firebrick4"))(15),ColSideColors =
```

```
colors_cor_Ad,scale="none",symm=T,margins=c(15,15),key=T,trace="none")
legend("topright",legend=c("Male","Female"),col=c("gray","black"),lty=1,lwd=10)

#All stages - correlation
Table_cor <- (subset(table1, select= c("Male_Cercariae_1_RPKM",
"Male_Cercariae_2_RPKM", "Female_Cercariae_1_RPKM",
"Female_Cercariae_2_RPKM","Male_Schistosomula_1_RPKM",
"Male_Schistosomula_2_RPKM", "Female_Schistosomula_1_RPKM",
"Female_Schistosomula_2_RPKM","Male_Adult_1_RPKM", "Male_Adult_2_RPKM",
"Female_Adult_1_RPKM", "Female_Adult_2_RPKM")))
colors_cor<-
c("gray","gray","black","black","gray","gray","black","black","gray","gray","bla
ck","black")
heatmap.2(cor(Table_cor,method="pearson"),col=colorRampPalette(c("blue","white",
"red","firebrick4"))(15),ColSideColors =
colors_cor,scale="none",symm=T,margins=c(15,15),key=T,trace="none")
legend("topright",legend=c("Male","Female"),col=c("gray","black"),lty=1,lwd=10)

## A.2. OVERALL EXPRESSION PLOTS BEFORE LOESS NORMALIZATION

#Cercariae - plot overall expression per samples
boxplot(table1$Male_Cercariae_1_RPKM, table1$Male_Cercariae_2_RPKM,
table1$Female_Cercariae_1_RPKM, table1$Female_Cercariae_2_RPKM, names =
c("M1","M2","F1","F2"),
col=c("darkseagreen","darkseagreen","lightsalmon","lightsalmon"),notch=T,
outline = F)
totaldata_Cerc <- as.numeric(paste(c(table1$Male_Cercariae_1_RPKM,
table1$Male_Cercariae_2_RPKM, table1$Female_Cercariae_1_RPKM,
table1$Female_Cercariae_2_RPKM)))
median_Cerc <- median(totaldata_Cerc,na.rm=T)
median_Cerc
abline(h=median_Cerc, lty=2, lwd=1, col="black")

#Schistosomula - plot overall expression per samples
boxplot(table1$Male_Schistosomula_1_RPKM, table1$Male_Schistosomula_2_RPKM,
table1$Female_Schistosomula_1_RPKM, table1$Female_Schistosomula_2_RPKM, names =
c("M1","M2","F1","F2"),
col=c("darkseagreen","darkseagreen","lightsalmon","lightsalmon" ),notch=T,
outline = F)
totaldata_Som <- as.numeric(paste(c(table1$Male_Schistosomula_1_RPKM,
table1$Male_Schistosomula_2_RPKM, table1$Female_Schistosomula_1_RPKM,
table1$Female_Schistosomula_2_RPKM)))
median_Som <- median(totaldata_Som,na.rm=T)
median_Som
abline(h=median_Som, lty=2, lwd=1, col="black")

#Adults - plot overall expression per samples
boxplot(table1$Male_Adult_1_RPKM, table1$Male_Adult_2_RPKM,
table1$Female_Adult_1_RPKM, table1$Female_Adult_2_RPKM, names =
c("M1","M2","F1","F2"),
col=c("darkseagreen","darkseagreen","lightsalmon","lightsalmon"),notch=T,
outline = F)
totaldata_Ad <- as.numeric(paste(c(table1$Male_Adult_1_RPKM,
table1$Male_Adult_2_RPKM, table1$Female_Adult_1_RPKM,
table1$Female_Adult_2_RPKM)))
median_Ad <- median(totaldata_Som,na.rm=T)
median_Ad
abline(h=median_Ad, lty=2, lwd=1, col="black")

#All stages - plot overall expression per samples
boxplot(table1$Male_Cercariae_1_RPKM, table1$Male_Cercariae_2_RPKM,
table1$Female_Cercariae_1_RPKM, table1$Female_Cercariae_2_RPKM,
table1$Male_Schistosomula_1_RPKM, table1$Male_Schistosomula_2_RPKM,
table1$Female_Schistosomula_1_RPKM, table1$Female_Schistosomula_2_RPKM,
```

```
table1$Male_Adult_1_RPKM, table1$Male_Adult_2_RPKM, table1$Female_Adult_1_RPKM,
table1$Female_Adult_2_RPKM, names =
c("Cerc_M1","Cerc_M2","Cerc_F1","Cerc_F2","Som_M1","Som_M2","Som_F1","Som_F2","A
d_M1","Ad_M2","Ad_F1","Ad_F2"),
col=c("darkseagreen","darkseagreen","lightsalmon","lightsalmon","darkseagreen","
darkseagreen","lightsalmon","lightsalmon","darkseagreen","darkseagreen","lightsa
lmon","lightsalmon"),notch=T, outline = F)
totaldata <- as.numeric(paste(c(table1$Male_Cercariae_1_RPKM,
table1$Male_Cercariae_2_RPKM, table1$Female_Cercariae_1_RPKM,
table1$Female_Cercariae_2_RPKM, table1$Male_Schistosomula_1_RPKM,
table1$Male_Schistosomula_2_RPKM, table1$Female_Schistosomula_1_RPKM,
table1$Female_Schistosomula_2_RPKM, table1$Male_Adult_1_RPKM,
table1$Male_Adult_2_RPKM, table1$Female_Adult_1_RPKM,
table1$Female_Adult_2_RPKM)))
median <- median(totaldata,na.rm=T)
median
abline(h=median, lty=2, lwd=1, col="black")

## A.3. LOESS NORMALIZATION

library(affy)

#Extract gene features
head(table1)
Gene_features <- subset(table1, select = c("Gene_ID","Location","First_base",
"Gene_Length"))

#Expression table without null data
table1_wo0 <- 0.000001 + (subset(table1, select = c("Male_Cercariae_1_RPKM",
"Male_Cercariae_2_RPKM", "Female_Cercariae_1_RPKM",
"Female_Cercariae_2_RPKM","Male_Schistosomula_1_RPKM",
"Male_Schistosomula_2_RPKM", "Female_Schistosomula_1_RPKM",
"Female_Schistosomula_2_RPKM","Male_Adult_1_RPKM", "Male_Adult_2_RPKM",
"Female_Adult_1_RPKM", "Female_Adult_2_RPKM")))
nrow(table1_wo0) #10787

#Obtain normalized table
table_normalized <- normalize.loess(table1_wo0)
table_normalized <- as.data.frame(table_normalized)
nrow(table_normalized)
Cerc_M1 <- (table_normalized[,1])
Cerc_M2 <- (table_normalized[,2])
Cerc_F1 <- (table_normalized[,3])
Cerc_F2 <- (table_normalized[,4])
Som_M1 <- (table_normalized[,5])
Som_M2 <- (table_normalized[,6])
Som_F1 <- (table_normalized[,7])
Som_F2 <- (table_normalized[,8])
Ad_M1 <- (table_normalized[,9])
Ad_M2 <- (table_normalized[,10])
Ad_F1 <- (table_normalized[,11])
Ad_F2 <- (table_normalized[,12])

#Print SuperTable_normalized
SuperTable_normalized_all <- cbind(Gene_features,
Cerc_F1,Cerc_F2,Cerc_M1,Cerc_M2,Som_F1,Som_F2,Som_M1,Som_M2,
Ad_F1,Ad_F2,Ad_M1,Ad_M2)
head(SuperTable_normalized_all)
nrow(SuperTable_normalized_all)
write.table(SuperTable_normalized_all,file="~/SupplementaryData5.txt")

SuperTable_normalized_all <-
read.table("~/SupplementaryData5.txt",fill=TRUE,header=TRUE) # Normalized table
attached as the Supplementary Data 5 /!\ It should be in a .txt format
```

```
head(SuperTable_normalized_all)
nrow(SuperTable_normalized_all) #10787

##A.4. OVERALL EXPRESSION PLOTS AFTER LOESS NORMALIZATION

boxplot(SuperTable_normalized_all$Cerc_F1, SuperTable_normalized_all$Cerc_F2,
SuperTable_normalized_all$Cerc_M1, SuperTable_normalized_all$Cerc_M2,
SuperTable_normalized_all$Som_F1, SuperTable_normalized_all$Som_F2,
SuperTable_normalized_all$Som_M1, SuperTable_normalized_all$Som_M2,
SuperTable_normalized_all$Ad_F1, SuperTable_normalized_all$Ad_F2,
SuperTable_normalized_all$Ad_M1, SuperTable_normalized_all$Ad_M2, names =
c("Cerc_M1","Cerc_M2","Cerc_F1","Cerc_F2","Som_M1","Som_M2","Som_F1","Som_F2","A
d_M1","Ad_M2","Ad_F1","Ad_F2"),
col=c("darkseagreen","darkseagreen","lightsalmon","lightsalmon","darkseagreen","
darkseagreen","lightsalmon","lightsalmon","darkseagreen","darkseagreen","lightsa
lmon","lightsalmon"),notch=T, outline = F)
totaldata <- as.numeric(paste(c(SuperTable_normalized_all$Cerc_F1,
SuperTable_normalized_all$Cerc_F2, SuperTable_normalized_all$Cerc_M1,
SuperTable_normalized_all$Cerc_M2, SuperTable_normalized_all$Som_F1,
SuperTable_normalized_all$Som_F2, SuperTable_normalized_all$Som_M1,
SuperTable_normalized_all$Som_M2, SuperTable_normalized_all$Ad_F1,
SuperTable_normalized_all$Ad_F2, SuperTable_normalized_all$Ad_M1,
SuperTable_normalized_all$Ad_M2)))
median <- median(totaldata,na.rm=T)
median
abline(h=median, lty=2, lwd=1, col="black")

##A.5. CORRELATION ANALYSIS ON NORMALIZED DATA

library(gplots)

#Cercariae - correlation
Table_cor_Cerc <- (subset(SuperTable_normalized_all, select= c("Cerc_F1",
"Cerc_F2", "Cerc_M1", "Cerc_M2")))

colors_cor_Cerc<- c("gray","gray","black","black")
heatmap.2(cor(Table_cor_Cerc,method="pearson"),col=colorRampPalette(c("blue","wh
ite","red","firebrick4"))(15),ColSideColors =
colors_cor_Cerc,scale="none",symm=T,margins=c(15,15),key=T,trace="none")
legend("topright",legend=c("Male","Female"),col=c("gray","black"),lty=1,lwd=10)

#Schistosomula - correlation
Table_cor_Som <- (subset(SuperTable_normalized_all, select= c("Som_F1",
"Som_F2", "Som_M1", "Som_M2")))

colors_cor_Som<- c("gray","gray","black","black")
heatmap.2(cor(Table_cor_Som,method="pearson"),col=colorRampPalette(c("blue","whi
te","red","firebrick4"))(15),ColSideColors =
colors_cor_Som,scale="none",symm=T,margins=c(15,15),key=T,trace="none")
legend("topright",legend=c("Male","Female"),col=c("gray","black"),lty=1,lwd=10)

#Adults - correlation
Table_cor_Ad <- (subset(SuperTable_normalized_all, select= c("Ad_F1", "Ad_F2",
"Ad_M1", "Ad_M2")))

colors_cor_Ad<- c("gray","gray","black","black")
heatmap.2(cor(Table_cor_Ad,method="pearson"),col=colorRampPalette(c("blue","whit
e","red","firebrick4"))(15),ColSideColors =
colors_cor_Ad,scale="none",symm=T,margins=c(15,15),key=T,trace="none")
legend("topright",legend=c("Male","Female"),col=c("gray","black"),lty=1,lwd=10)

##########################
# B. EXPRESSION ANALYSIS #
##########################
```

```
#Open data table
SuperTable_normalized_all <-
read.table("~/SupplementaryData5.txt",fill=TRUE,header=TRUE) # Normalized table
attached as the Supplementary Data 5 /!\ It should be in a .txt format
head(SuperTable_normalized_all)
nrow(SuperTable_normalized_all) #10787

## B.1. AVERAGE BIOLOGICAL REPLICATES & CALCULATE RATIOS

# female average per stage
SuperTable_normalized_all$Fmean_Cerc <-
as.numeric(paste(SuperTable_normalized_all$Cerc_F1+SuperTable_normalized_all$Cer
c_F2))/2
SuperTable_normalized_all$Fmean_Som <-
as.numeric(paste(SuperTable_normalized_all$Som_F1+SuperTable_normalized_all$Som_
F2))/2
SuperTable_normalized_all$Fmean_Ad <-
as.numeric(paste(SuperTable_normalized_all$Ad_F1+SuperTable_normalized_all$Ad_F2
))/2
# male average per stage
SuperTable_normalized_all$Mmean_Cerc <-
as.numeric(paste(SuperTable_normalized_all$Cerc_M1+SuperTable_normalized_all$Cer
c_M2))/2
SuperTable_normalized_all$Mmean_Som <-
as.numeric(paste(SuperTable_normalized_all$Som_M1+SuperTable_normalized_all$Som_
M2))/2
SuperTable_normalized_all$Mmean_Ad <-
as.numeric(paste(SuperTable_normalized_all$Ad_M1+SuperTable_normalized_all$Ad_M2
))/2
# female:male ratio (F:M ratio) per stage
SuperTable_normalized_all$FMratio_Cerc <-
as.numeric(paste(SuperTable_normalized_all$Fmean_Cerc))/as.numeric(paste(SuperTa
ble_normalized_all$Mmean_Cerc))
SuperTable_normalized_all$FMratio_Som <-
as.numeric(paste(SuperTable_normalized_all$Fmean_Som))/as.numeric(paste(SuperTab
le_normalized_all$Mmean_Som))
SuperTable_normalized_all$FMratio_Ad <-
as.numeric(paste(SuperTable_normalized_all$Fmean_Ad))/as.numeric(paste(SuperTabl
e_normalized_all$Mmean_Ad))
# log2(F:M ratio) per stage
SuperTable_normalized_all$FMlog_Cerc <-
log2(as.numeric(paste(SuperTable_normalized_all$FMratio_Cerc)))
SuperTable_normalized_all$FMlog_Som <-
log2(as.numeric(paste(SuperTable_normalized_all$FMratio_Som)))
SuperTable_normalized_all$FMlog_Ad <-
log2(as.numeric(paste(SuperTable_normalized_all$FMratio_Ad)))

## B.2. FILTERS ON MINIMUM OF EXPRESSION LEVEL

#Define filter
explim <- 1 #Arbitrary treshold for minimum level of expression (e.g. null
expression=0.000001)

#Filter per stage
ST_norm_Cerc <- subset(SuperTable_normalized_all, Fmean_Cerc>explim &
Mmean_Cerc>explim)
nrow(ST_norm_Cerc) # 5870 for RPKM1
ST_norm_Som <- subset(SuperTable_normalized_all, Fmean_Som>explim &
Mmean_Som>explim)
nrow(ST_norm_Som) # 6253 for RPKM1
ST_norm_Ad <- subset(SuperTable_normalized_all, Fmean_Ad>explim &
Mmean_Ad>explim)
nrow(ST_norm_Ad) # 6256 for RPKM1
```

```
#Optional: exclude sex bias (Fold change higher than 2)
ST_norm_Cerc <- subset(ST_norm_Cerc, FMratio_Cerc<2 & FMratio_Cerc>0.5)
nrow(ST_norm_Cerc) #3741
ST_norm_Som <- subset(ST_norm_Som, FMratio_Som<2 & FMratio_Som>0.5)
nrow(ST_norm_Som) #5636
ST_norm_Ad <- subset(ST_norm_Ad, FMratio_Ad<2 & FMratio_Ad>0.5)
nrow(ST_norm_Ad) #5657


## B.3. SUBSET TABLES

#Cercariae - Tables by location
ST_norm_Cerc_Chr1 <- subset(ST_norm_Cerc, Location=="Chr_1")
nrow(ST_norm_Cerc_Chr1) # 1105 rpkm1
ST_norm_Cerc_Chr2 <- subset(ST_norm_Cerc, Location=="Chr_2")
nrow(ST_norm_Cerc_Chr2) # 547 rpkm1
ST_norm_Cerc_Chr3 <- subset(ST_norm_Cerc, Location=="Chr_3")
nrow(ST_norm_Cerc_Chr3) # 495 rpkm1
ST_norm_Cerc_Chr4  <- subset(ST_norm_Cerc, Location=="Chr_4")
nrow(ST_norm_Cerc_Chr4) # 476 rpkm1
ST_norm_Cerc_Chr5 <- subset(ST_norm_Cerc, Location=="Chr_5")
nrow(ST_norm_Cerc_Chr5) # 131 rpkm1
ST_norm_Cerc_Chr6 <- subset(ST_norm_Cerc, Location=="Chr_6")
nrow(ST_norm_Cerc_Chr6) # 301 rpkm1
ST_norm_Cerc_Chr7 <- subset(ST_norm_Cerc, Location=="Chr_7")
nrow(ST_norm_Cerc_Chr7) # 90 rpkm1
ST_norm_Cerc_Aut <- subset(ST_norm_Cerc, Location=="Chr_1" |Location=="Chr_2" |
Location=="Chr_3" |Location=="Chr_4" |Location=="Chr_5" |Location=="Chr_6" |
Location=="Chr_7" )
nrow(ST_norm_Cerc_Aut) # 3145 rpkm1
ST_norm_Cerc_Z <- subset(ST_norm_Cerc, Location=="Chr_Z")
nrow(ST_norm_Cerc_Z) # 474 rpkm1
ST_norm_Cerc_PAR <- subset(ST_norm_Cerc, Location=="Chr_PAR")
nrow(ST_norm_Cerc_PAR) # 582 rpkm1
ST_norm_Cerc_Sex <- subset(ST_norm_Cerc, Location=="Chr_Z" |
Location=="Chr_PAR" )
nrow(ST_norm_Cerc_Sex) # 1056 rpkm1

#Schistosomula - Tables by location
ST_norm_Som_Chr1 <- subset(ST_norm_Som, Location=="Chr_1")
nrow(ST_norm_Som_Chr1) # 1168 rpkm1
ST_norm_Som_Chr2 <- subset(ST_norm_Som, Location=="Chr_2")
nrow(ST_norm_Som_Chr2) # 591 rpkm1
ST_norm_Som_Chr3 <- subset(ST_norm_Som, Location=="Chr_3")
nrow(ST_norm_Som_Chr3) # 523 rpkm1
ST_norm_Som_Chr4  <- subset(ST_norm_Som, Location=="Chr_4")
nrow(ST_norm_Som_Chr4) # 505 rpkm1
ST_norm_Som_Chr5 <- subset(ST_norm_Som, Location=="Chr_5")
nrow(ST_norm_Som_Chr5) # 144 rpkm1
ST_norm_Som_Chr6 <- subset(ST_norm_Som, Location=="Chr_6")
nrow(ST_norm_Som_Chr6) # 322 rpkm1
ST_norm_Som_Chr7 <- subset(ST_norm_Som, Location=="Chr_7")
nrow(ST_norm_Som_Chr7) # 101 rpkm1
ST_norm_Som_Aut <- subset(ST_norm_Som, Location=="Chr_1" |Location=="Chr_2" |
Location=="Chr_3" |Location=="Chr_4" |Location=="Chr_5" |Location=="Chr_6" |
Location=="Chr_7" )
nrow(ST_norm_Som_Aut) # 3354 rpkm1
ST_norm_Som_Z <- subset(ST_norm_Som, Location=="Chr_Z")
nrow(ST_norm_Som_Z) # 492 rpkm1
ST_norm_Som_PAR <- subset(ST_norm_Som, Location=="Chr_PAR")
nrow(ST_norm_Som_PAR) # 618 rpkm1
ST_norm_Som_Sex <- subset(ST_norm_Som, Location=="Chr_Z" |Location=="Chr_PAR" )
nrow(ST_norm_Som_Sex) # 1110 rpkm1
```

```
#Adults - Tables by location
ST_norm_Ad_Chr1 <- subset(ST_norm_Ad, Location=="Chr_1")
nrow(ST_norm_Ad_Chr1) # 1160 rpkm1
ST_norm_Ad_Chr2 <- subset(ST_norm_Ad, Location=="Chr_2")
nrow(ST_norm_Ad_Chr2) # 590 rpkm1
ST_norm_Ad_Chr3 <- subset(ST_norm_Ad, Location=="Chr_3")
nrow(ST_norm_Ad_Chr3) # 523 rpkm1
ST_norm_Ad_Chr4  <- subset(ST_norm_Ad, Location=="Chr_4")
nrow(ST_norm_Ad_Chr4) # 513 rpkm1
ST_norm_Ad_Chr5 <- subset(ST_norm_Ad, Location=="Chr_5")
nrow(ST_norm_Ad_Chr5) # 143 rpkm1
ST_norm_Ad_Chr6 <- subset(ST_norm_Ad, Location=="Chr_6")
nrow(ST_norm_Ad_Chr6) # 323 rpkm1
ST_norm_Ad_Chr7 <- subset(ST_norm_Ad, Location=="Chr_7")
nrow(ST_norm_Ad_Chr7) # 109 rpkm1
ST_norm_Ad_Aut <- subset(ST_norm_Ad, Location=="Chr_1" |Location=="Chr_2" |
Location=="Chr_3" |Location=="Chr_4" |Location=="Chr_5" |Location=="Chr_6" |
Location=="Chr_7" )
nrow(ST_norm_Ad_Aut) # 3361 rpkm1
ST_norm_Ad_Z <- subset(ST_norm_Ad, Location=="Chr_Z")
nrow(ST_norm_Ad_Z) # 497 rpkm1
ST_norm_Ad_PAR <- subset(ST_norm_Ad, Location=="Chr_PAR")
nrow(ST_norm_Ad_PAR) # 608 rpkm1
ST_norm_Ad_Sex <- subset(ST_norm_Ad, Location=="Chr_Z" |Location=="Chr_PAR" )
nrow(ST_norm_Ad_Sex) # 1105 rpkm1


## B.4. PLOTS AND STATS

# B.4.1. Comparative analysis per sex (FIGURE 1)

# Males
boxplot(log2(ST_norm_Cerc_Aut$Mmean_Cerc), log2(ST_norm_Cerc_Z$Mmean_Cerc),
log2(ST_norm_Som_Aut$Mmean_Som),log2(ST_norm_Som_Z$Mmean_Som),
log2(ST_norm_Ad_Aut$Mmean_Ad),log2(ST_norm_Ad_Z$Mmean_Ad),ylim=c(0,10), names =
c("AA","ZZ","AA","ZZ","AA","ZZ"), ylab="log2(RPKM)",cex.axis=1.5, cex.lab=1.5,
notch=T, outline = F,
col=c("lightcyan4","lightcyan3","lightcyan4","lightcyan3","lightcyan4","lightcya
n3"), main="A. Males",cex.main=2)
boxplot(log2(ST_norm_Cerc_Aut$Mmean_Cerc),log2(ST_norm_Cerc_PAR$Mmean_Cerc),
log2(ST_norm_Cerc_Z$Mmean_Cerc),
log2(ST_norm_Som_Aut$Mmean_Som),log2(ST_norm_Som_PAR$Mmean_Som),
log2(ST_norm_Som_Z$Mmean_Som),
log2(ST_norm_Ad_Aut$Mmean_Ad),log2(ST_norm_Ad_PAR$Mmean_Ad),
log2(ST_norm_Ad_Z$Mmean_Ad), names =
c("AA","PAR","ZZ","AA","PAR","ZZ","AA","PAR","ZZ"),
ylab="log2(RPKM)",ylim=c(0,10),cex.axis=1.5, cex.lab=1.5, notch=T, outline = F,
col=c("lightcyan4","lightcyan4","lightcyan3","lightcyan4","lightcyan4","lightcya
n3","lightcyan4","lightcyan4","lightcyan3"), main="A. Males",cex.main=2)
abline(v=3.5)
abline(v=6.5)

median(ST_norm_Cerc_Z$Mmean_Cerc)
median(ST_norm_Cerc_Aut$Mmean_Cerc)
median(ST_norm_Cerc_PAR$Mmean_Cerc)
median(ST_norm_Som_Z$Mmean_Som)
median(ST_norm_Som_Aut$Mmean_Som)
median(ST_norm_Som_PAR$Mmean_Som)
median(ST_norm_Ad_Z$Mmean_Ad)
median(ST_norm_Ad_Aut$Mmean_Ad)
median(ST_norm_Ad_PAR$Mmean_Ad)

wilcox.test(ST_norm_Cerc_Z$Mmean_Cerc,ST_norm_Cerc_Aut$Mmean_Cerc)$p.value
```

```
median(ST_norm_Cerc_Z$Mmean_Cerc) / median(ST_norm_Cerc_Aut$Mmean_Cerc)
wilcox.test(ST_norm_Som_Z$Mmean_Som,ST_norm_Som_Aut$Mmean_Som)$p.value
median(ST_norm_Som_Z$Mmean_Som) / median(ST_norm_Som_Aut$Mmean_Som)
wilcox.test(ST_norm_Ad_Z$Mmean_Ad,ST_norm_Ad_Aut$Mmean_Ad)$p.value
median(ST_norm_Ad_Z$Mmean_Ad) / median(ST_norm_Ad_Aut$Mmean_Ad)

wilcox.test(ST_norm_Som_Z$Mmean_Som,ST_norm_Cerc_Z$Mmean_Cerc)$p.value
median(ST_norm_Som_Z$Mmean_Som) / median(ST_norm_Cerc_Z$Mmean_Cerc)
wilcox.test(ST_norm_Ad_Z$Mmean_Ad,ST_norm_Som_Z$Mmean_Som)$p.value
median(ST_norm_Ad_Z$Mmean_Ad) / median(ST_norm_Som_Z$Mmean_Som)
wilcox.test(ST_norm_Ad_Z$Mmean_Ad,ST_norm_Cerc_Z$Mmean_Cerc)$p.value
median(ST_norm_Ad_Z$Mmean_Ad) / median(ST_norm_Cerc_Z$Mmean_Cerc)

wilcox.test(ST_norm_Som_Aut$Mmean_Som,ST_norm_Cerc_Aut$Mmean_Cerc)$p.value
median(ST_norm_Som_Aut$Mmean_Som) / median(ST_norm_Cerc_Aut$Mmean_Cerc)
wilcox.test(ST_norm_Ad_Aut$Mmean_Ad,ST_norm_Som_Aut$Mmean_Som)$p.value
median(ST_norm_Ad_Aut$Mmean_Ad) / median(ST_norm_Som_Aut$Mmean_Som)
wilcox.test(ST_norm_Ad_Aut$Mmean_Ad,ST_norm_Cerc_Aut$Mmean_Cerc)$p.value
median(ST_norm_Ad_Aut$Mmean_Ad) / median(ST_norm_Cerc_Aut$Mmean_Cerc)

# Females
boxplot(log2(ST_norm_Cerc_Aut$Fmean_Cerc), log2(ST_norm_Cerc_Z$Fmean_Cerc),
log2(ST_norm_Som_Aut$Fmean_Som), log2(ST_norm_Som_Z$Fmean_Som),
log2(ST_norm_Ad_Aut$Fmean_Ad), log2(ST_norm_Ad_Z$Fmean_Ad),ylim=c(0,10), names =
c("AA","Z","AA","Z","AA","Z"), ylab="log2(RPKM)",cex.axis=1.5, cex.lab=1.5,
notch=T, outline = F,
col=c("hotpink4","hotpink3","hotpink4","hotpink3","hotpink4","hotpink3"),
main="B. Females",cex.main=2)
boxplot(log2(ST_norm_Cerc_Aut$Fmean_Cerc),log2(ST_norm_Cerc_PAR$Fmean_Cerc),
log2(ST_norm_Cerc_Z$Fmean_Cerc),
log2(ST_norm_Som_Aut$Fmean_Som),log2(ST_norm_Som_PAR$Fmean_Som),
log2(ST_norm_Som_Z$Fmean_Som),
log2(ST_norm_Ad_Aut$Fmean_Ad),log2(ST_norm_Ad_PAR$Fmean_Ad),
log2(ST_norm_Ad_Z$Fmean_Ad),ylim=c(0,10), names =
c("AA","PAR","Z","AA","PAR","Z","AA","PAR","Z"), ylab="log2(RPKM)",cex.axis=1.5,
cex.lab=1.5, notch=T, outline = F,
col=c("hotpink4","hotpink4","hotpink3","hotpink4","hotpink4","hotpink3","hotpink
4","hotpink4","hotpink3"), main="B. Females",cex.main=2)
abline(v=3.5)
abline(v=6.5)

median(ST_norm_Cerc_Z$Fmean_Cerc)
median(ST_norm_Cerc_Aut$Fmean_Cerc)
median(ST_norm_Cerc_PAR$Fmean_Cerc)
median(ST_norm_Som_Z$Fmean_Som)
median(ST_norm_Som_Aut$Fmean_Som)
median(ST_norm_Som_PAR$Fmean_Som)
median(ST_norm_Ad_Z$Fmean_Ad)
median(ST_norm_Ad_Aut$Fmean_Ad)
median(ST_norm_Ad_PAR$Fmean_Ad)

wilcox.test(ST_norm_Cerc_Z$Fmean_Cerc,ST_norm_Cerc_Aut$Fmean_Cerc)$p.value
median(ST_norm_Cerc_Z$Fmean_Cerc) / median(ST_norm_Cerc_Aut$Fmean_Cerc)
wilcox.test(ST_norm_Som_Z$Fmean_Som,ST_norm_Som_Aut$Fmean_Som)$p.value
median(ST_norm_Som_Z$Fmean_Som) / median(ST_norm_Som_Aut$Fmean_Som)
wilcox.test(ST_norm_Ad_Z$Fmean_Ad,ST_norm_Ad_Aut$Fmean_Ad)$p.value
median(ST_norm_Ad_Z$Fmean_Ad) / median(ST_norm_Ad_Aut$Fmean_Ad)

wilcox.test(ST_norm_Som_Z$Fmean_Som,ST_norm_Cerc_Z$Fmean_Cerc)$p.value
median(ST_norm_Som_Z$Fmean_Som) / median(ST_norm_Cerc_Z$Fmean_Cerc)
wilcox.test(ST_norm_Ad_Z$Fmean_Ad,ST_norm_Som_Z$Fmean_Som)$p.value
median(ST_norm_Ad_Z$Fmean_Ad) / median(ST_norm_Som_Z$Fmean_Som)
wilcox.test(ST_norm_Ad_Z$Fmean_Ad,ST_norm_Cerc_Z$Fmean_Cerc)$p.value
median(ST_norm_Ad_Z$Fmean_Ad) / median(ST_norm_Cerc_Z$Fmean_Cerc)
```

```
wilcox.test(ST_norm_Som_Aut$Fmean_Som,ST_norm_Cerc_Aut$Fmean_Cerc)$p.value
median(ST_norm_Som_Aut$Fmean_Som) / median(ST_norm_Cerc_Aut$Fmean_Cerc)
wilcox.test(ST_norm_Ad_Aut$Fmean_Ad,ST_norm_Som_Aut$Fmean_Som)$p.value
median(ST_norm_Ad_Aut$Fmean_Ad) / median(ST_norm_Som_Aut$Fmean_Som)
wilcox.test(ST_norm_Ad_Aut$Fmean_Ad,ST_norm_Cerc_Aut$Fmean_Cerc)$p.value
median(ST_norm_Ad_Aut$Fmean_Ad) / median(ST_norm_Cerc_Aut$Fmean_Cerc)

# Females:Males
boxplot((ST_norm_Cerc_Aut$FMlog_Cerc),(ST_norm_Cerc_PAR$FMlog_Cerc),
(ST_norm_Cerc_Z$FMlog_Cerc),(ST_norm_Som_Aut$FMlog_Som),
(ST_norm_Som_PAR$FMlog_Som),(ST_norm_Som_Z$FMlog_Som),(ST_norm_Ad_Aut$FMlog_Ad),
(ST_norm_Ad_PAR$FMlog_Ad),(ST_norm_Ad_Z$FMlog_Ad), names =
c("A","PAR","Z","A","PAR","Z","A","PAR","Z"), ylab="log2(F:M)", cex.axis=1.5,
cex.lab=1.5, notch=T, outline = F,
col=c("wheat3","wheat3","wheat","wheat3","wheat3","wheat","wheat3","wheat3","whe
at"), main="Female:Male ratio of expression", cex.main=2)
boxplot((ST_norm_Cerc_Aut$FMlog_Cerc),(ST_norm_Cerc_Z$FMlog_Cerc),
(ST_norm_Som_Aut$FMlog_Som),(ST_norm_Som_Z$FMlog_Som),(ST_norm_Ad_Aut$FMlog_Ad),
(ST_norm_Ad_Z$FMlog_Ad), names =
c("AA:AA","Z:ZZ","AA:AA","Z:ZZ","AA:AA","Z:ZZ"), ylab="log2(F:M)", cex.axis=1.5,
cex.lab=1.5, notch=T, outline = F,
col=c("wheat3","wheat","wheat3","wheat","wheat3","wheat"), main="Female:Male
ratio of expression", cex.main=2)
abline(v=3.5)
abline(v=6.5)

median(ST_norm_Cerc_Z$FMratio_Cerc)
median(ST_norm_Cerc_Aut$FMratio_Cerc)
median(ST_norm_Cerc_PAR$FMratio_Cerc)
median(ST_norm_Som_Z$FMratio_Som)
median(ST_norm_Som_Aut$FMratio_Som)
median(ST_norm_Som_PAR$FMratio_Som)
median(ST_norm_Ad_Z$FMratio_Ad)
median(ST_norm_Ad_Aut$FMratio_Ad)
median(ST_norm_Ad_PAR$FMratio_Ad)

wilcox.test(ST_norm_Cerc_Z$FMratio_Cerc,ST_norm_Cerc_Aut$FMratio_Cerc)$p.value
median(ST_norm_Cerc_Z$FMratio_Cerc) / median(ST_norm_Cerc_Aut$FMratio_Cerc)
wilcox.test(ST_norm_Som_Z$FMratio_Som,ST_norm_Som_Aut$FMratio_Som)$p.value
median(ST_norm_Som_Z$FMratio_Som) / median(ST_norm_Som_Aut$FMratio_Som)
wilcox.test(ST_norm_Ad_Z$FMratio_Ad,ST_norm_Ad_Aut$FMratio_Ad)$p.value
median(ST_norm_Ad_Z$FMratio_Ad) / median(ST_norm_Ad_Aut$FMratio_Ad)

wilcox.test(ST_norm_Som_Z$FMratio_Som,ST_norm_Cerc_Z$FMratio_Cerc)$p.value
median(ST_norm_Som_Z$FMratio_Som) / median(ST_norm_Cerc_Z$FMratio_Cerc)
wilcox.test(ST_norm_Ad_Z$FMratio_Ad,ST_norm_Som_Z$FMratio_Som)$p.value
median(ST_norm_Ad_Z$FMratio_Ad) / median(ST_norm_Som_Z$FMratio_Som)
wilcox.test(ST_norm_Ad_Z$FMratio_Ad,ST_norm_Cerc_Z$FMratio_Cerc)$p.value
median(ST_norm_Ad_Z$FMratio_Ad) / median(ST_norm_Cerc_Z$FMratio_Cerc)

wilcox.test(ST_norm_Som_Aut$FMratio_Som,ST_norm_Cerc_Aut$FMratio_Cerc)$p.value
median(ST_norm_Som_Aut$FMratio_Som) / median(ST_norm_Cerc_Aut$FMratio_Cerc)
wilcox.test(ST_norm_Ad_Aut$FMratio_Ad,ST_norm_Som_Aut$FMratio_Som)$p.value
median(ST_norm_Ad_Aut$FMratio_Ad) / median(ST_norm_Som_Aut$FMratio_Som)
wilcox.test(ST_norm_Ad_Aut$FMratio_Ad,ST_norm_Cerc_Aut$FMratio_Cerc)$p.value
median(ST_norm_Ad_Aut$FMratio_Ad) / median(ST_norm_Cerc_Aut$FMratio_Cerc)


# B.4.2. Comparative analysis per stage (SUPPLEMENTARY FIGURES 1 & 2, left panel
; SUPPLEMENTARY FIGURE 5)

#Cercariae
boxplot((ST_norm_Cerc_Aut$FMlog_Cerc),(ST_norm_Cerc_PAR$FMlog_Cerc),
```

```
(ST_norm_Cerc_Z$FMlog_Cerc), names = c("A (n=3145)","PAR (n=1056)","Z (n=474)"),
ylab="log2(F:M)",ylim=c(-3,3), cex.axis=1.25, cex.lab=1.5, notch=T, outline = F,
col=c("wheat3","wheat3","wheat"), main="A. Cercariae", cex.main=2) # No filter
boxplot((ST_norm_Cerc_Aut$FMlog_Cerc),(ST_norm_Cerc_PAR$FMlog_Cerc),
(ST_norm_Cerc_Z$FMlog_Cerc), names = c("A (n=2016)","PAR (n=393)","Z (n=285)"),
ylab="log2(F:M)",ylim=c(-1.5,1.5), cex.axis=1.25, cex.lab=1.5, notch=T, outline
= F, col=c("wheat3","wheat3","wheat"), main="A. Cercariae", cex.main=2) #
Without sex-bias

wilcox.test(ST_norm_Cerc_Z$FMratio_Cerc,ST_norm_Cerc_Aut$FMratio_Cerc)$p.value
median(ST_norm_Cerc_Z$FMratio_Cerc) / median(ST_norm_Cerc_Aut$FMratio_Cerc)
wilcox.test(ST_norm_Cerc_Z$FMratio_Cerc,ST_norm_Cerc_PAR$FMratio_Cerc)$p.value
median(ST_norm_Cerc_Z$FMratio_Cerc) / median(ST_norm_Cerc_PAR$FMratio_Cerc)
wilcox.test(ST_norm_Cerc_PAR$FMratio_Cerc,ST_norm_Cerc_Aut$FMratio_Cerc)$p.value
median(ST_norm_Cerc_PAR$FMratio_Cerc) / median(ST_norm_Cerc_Aut$FMratio_Cerc)

boxplot((ST_norm_Cerc_Chr1$FMlog_Cerc),(ST_norm_Cerc_PAR$FMlog_Cerc),
(ST_norm_Cerc_Z$FMlog_Cerc), names = c("Chr1","PAR","Z"),
ylab="log2(F:M)",ylim=c(-3,3), cex.axis=1.5, cex.lab=1.5, notch=T, outline = F,
col=c("wheat3","wheat3","wheat"), main="A. Cercariae", cex.main=2) # Sup. figure

wilcox.test(ST_norm_Cerc_Z$FMratio_Cerc,ST_norm_Cerc_Chr1$FMratio_Cerc)$p.value
median(ST_norm_Cerc_Z$FMratio_Cerc) / median(ST_norm_Cerc_Chr1$FMratio_Cerc)
wilcox.test(ST_norm_Cerc_PAR$FMratio_Cerc,ST_norm_Cerc_Chr1$FMratio_Cerc)
$p.value
median(ST_norm_Cerc_PAR$FMratio_Cerc) / median(ST_norm_Cerc_Chr1$FMratio_Cerc)

#Schistosomula
boxplot((ST_norm_Som_Aut$FMlog_Som), (ST_norm_Som_PAR$FMlog_Som),
(ST_norm_Som_Z$FMlog_Som), names = c("A (n=3354)","PAR (n=618)","Z (n=492)"),
ylab="log2(F:M)",ylim=c(-3,3), cex.axis=1.25, cex.lab=1.5, notch=T, outline = F,
col=c("wheat3","wheat3","wheat"), main="B. Schistosomula", cex.main=2) # No
filter
boxplot((ST_norm_Som_Aut$FMlog_Som), (ST_norm_Som_PAR$FMlog_Som),
(ST_norm_Som_Z$FMlog_Som), names = c("A (n=3125)","PAR (n=575)","Z (n=360)"),
ylab="log2(F:M)",ylim=c(-1.5,1.5), cex.axis=1.25, cex.lab=1.5, notch=T, outline
= F, col=c("wheat3","wheat3","wheat"), main="B. Schistosomula", cex.main=2) #
Without sex-bias

wilcox.test(ST_norm_Som_Z$FMratio_Som,ST_norm_Som_Aut$FMratio_Som)$p.value
median(ST_norm_Som_Z$FMratio_Som) / median(ST_norm_Som_Aut$FMratio_Som)
wilcox.test(ST_norm_Som_Z$FMratio_Som,ST_norm_Som_PAR$FMratio_Som)$p.value
median(ST_norm_Som_Z$FMratio_Som) / median(ST_norm_Som_PAR$FMratio_Som)
wilcox.test(ST_norm_Som_PAR$FMratio_Som,ST_norm_Som_Aut$FMratio_Som)$p.value
median(ST_norm_Som_PAR$FMratio_Som) / median(ST_norm_Som_Aut$FMratio_Som)

boxplot((ST_norm_Som_Chr1$FMlog_Som), (ST_norm_Som_PAR$FMlog_Som),
(ST_norm_Som_Z$FMlog_Som), names = c("Chr1","PAR","Z"),
ylab="log2(F:M)",ylim=c(-3,3), cex.axis=1.5, cex.lab=1.5, notch=T, outline = F,
col=c("wheat3","wheat3","wheat"), main="B. Schistosomula", cex.main=2) # Sup.
figure

wilcox.test(ST_norm_Som_Z$FMratio_Som,ST_norm_Som_Chr1$FMratio_Som)$p.value
median(ST_norm_Som_Z$FMratio_Som) / median(ST_norm_Som_Chr1$FMratio_Som)
wilcox.test(ST_norm_Som_PAR$FMratio_Som,ST_norm_Som_Chr1$FMratio_Som)$p.value
median(ST_norm_Som_PAR$FMratio_Som) / median(ST_norm_Som_Chr1$FMratio_Som)

#Adult
boxplot((ST_norm_Ad_Aut$FMlog_Ad),(ST_norm_Ad_PAR$FMlog_Ad),
(ST_norm_Ad_Z$FMlog_Ad), names = c("A (n=3361)","PAR (n=608)","Z (n=497)"),
ylab="log2(F:M)",ylim=c(-3,3), cex.axis=1.25, cex.lab=1.5, notch=T, outline = F,
col=c("wheat3","wheat3","wheat"), main="C. Adult", cex.main=2) # No filter
boxplot((ST_norm_Ad_Aut$FMlog_Ad),(ST_norm_Ad_PAR$FMlog_Ad),
(ST_norm_Ad_Z$FMlog_Ad), names = c("A (n=3096)","PAR (n=561)","Z (n=384)"),
```

```
ylab="log2(F:M)",ylim=c(-1.5,1.5), cex.axis=1.25, cex.lab=1.5, notch=T, outline
= F, col=c("wheat3","wheat3","wheat"), main="C. Adult", cex.main=2)# Without
sex-bias

wilcox.test(ST_norm_Ad_Z$FMratio_Ad,ST_norm_Ad_Aut$FMratio_Ad)$p.value
median(ST_norm_Ad_Z$FMratio_Ad) / median(ST_norm_Ad_Aut$FMratio_Ad)
wilcox.test(ST_norm_Ad_Z$FMratio_Ad,ST_norm_Ad_PAR$FMratio_Ad)$p.value
median(ST_norm_Ad_Z$FMratio_Ad) / median(ST_norm_Ad_PAR$FMratio_Ad)
wilcox.test(ST_norm_Ad_PAR$FMratio_Ad,ST_norm_Ad_Aut$FMratio_Ad)$p.value
median(ST_norm_Ad_PAR$FMratio_Ad) / median(ST_norm_Ad_Aut$FMratio_Ad)

boxplot((ST_norm_Ad_Chr1$FMlog_Ad),(ST_norm_Ad_PAR$FMlog_Ad),
(ST_norm_Ad_Z$FMlog_Ad), names = c("Chr1","PAR","Z"), ylab="log2(F:M)",ylim=c(-
3,3), cex.axis=1.5, cex.lab=1.5, notch=T, outline = F,
col=c("wheat3","wheat3","wheat"), main="C. Adult", cex.main=2) # Sup. figure

wilcox.test(ST_norm_Ad_Z$FMratio_Ad,ST_norm_Ad_Chr1$FMratio_Ad)$p.value
median(ST_norm_Ad_Z$FMratio_Ad) / median(ST_norm_Ad_Chr1$FMratio_Ad)
wilcox.test(ST_norm_Ad_PAR$FMratio_Ad,ST_norm_Ad_Chr1$FMratio_Ad)$p.value
median(ST_norm_Ad_PAR$FMratio_Ad) / median(ST_norm_Ad_Chr1$FMratio_Ad)


# B.4.3. F:M ratio of expression along the Z, per stage (SUPPLEMENTARY FIGURES 1
& 2, RIGTH PANEL)

# /!\/!\/!\ For the following analysis, we will use both tables with and without
sex bias: you should not exclude sex bias in part B.2
ST_norm_Cerc_noSB <- subset(ST_norm_Cerc, FMratio_Cerc<2 & FMratio_Cerc>0.5)
nrow(ST_norm_Cerc_noSB) #3741
ST_norm_Som_noSB  <- subset(ST_norm_Som, FMratio_Som<2 & FMratio_Som>0.5)
nrow(ST_norm_Som_noSB) #5636
ST_norm_Ad_noSB  <- subset(ST_norm_Ad, FMratio_Ad<2 & FMratio_Ad>0.5)
nrow(ST_norm_Ad_noSB) #5657

ST_norm_Cerc_Aut_noSB <- subset(ST_norm_Cerc_noSB, Location=="Chr_1" |
Location=="Chr_2" |Location=="Chr_3" |Location=="Chr_4" |Location=="Chr_5" |
Location=="Chr_6" |Location=="Chr_7" )
nrow(ST_norm_Cerc_Aut_noSB)
ST_norm_Cerc_Z_noSB <- subset(ST_norm_Cerc_noSB, Location=="Chr_Z")
nrow(ST_norm_Cerc_Z_noSB)
ST_norm_Cerc_PAR_noSB <- subset(ST_norm_Cerc_noSB, Location=="Chr_PAR")
nrow(ST_norm_Cerc_PAR_noSB)
ST_norm_Cerc_Sex_noSB <- subset(ST_norm_Cerc_noSB, Location=="Chr_Z" |
Location=="Chr_PAR" )
nrow(ST_norm_Cerc_Sex_noSB)

ST_norm_Som_Aut_noSB <- subset(ST_norm_Som_noSB, Location=="Chr_1" |
Location=="Chr_2" |Location=="Chr_3" |Location=="Chr_4" |Location=="Chr_5" |
Location=="Chr_6" |Location=="Chr_7" )
nrow(ST_norm_Som_Aut_noSB)
ST_norm_Som_Z_noSB <- subset(ST_norm_Som_noSB, Location=="Chr_Z")
nrow(ST_norm_Som_Z_noSB)
ST_norm_Som_PAR_noSB <- subset(ST_norm_Som_noSB, Location=="Chr_PAR")
nrow(ST_norm_Som_PAR_noSB)
ST_norm_Som_Sex_noSB <- subset(ST_norm_Som_noSB, Location=="Chr_Z" |
Location=="Chr_PAR" )
nrow(ST_norm_Som_Sex_noSB)

ST_norm_Ad_Aut_noSB <- subset(ST_norm_Ad_noSB, Location=="Chr_1" |
Location=="Chr_2" |Location=="Chr_3" |Location=="Chr_4" |Location=="Chr_5" |
Location=="Chr_6" |Location=="Chr_7" )
nrow(ST_norm_Ad_Aut_noSB)
ST_norm_Ad_Z_noSB <- subset(ST_norm_Ad_noSB, Location=="Chr_Z")
nrow(ST_norm_Ad_Z_noSB)
```

```
ST_norm_Ad_PAR_noSB <- subset(ST_norm_Ad_noSB, Location=="Chr_PAR")
nrow(ST_norm_Ad_PAR_noSB)
ST_norm_Ad_Sex_noSB <- subset(ST_norm_Ad_noSB, Location=="Chr_Z" |
Location=="Chr_PAR" )
nrow(ST_norm_Ad_Sex_noSB)

# Cercariae

# Sort tables depending on First Base /!\/!\/!\
ST_norm_Cerc_Sex_noSB <-
ST_norm_Cerc_Sex_noSB[order(ST_norm_Cerc_Sex_noSB$First_base,decreasing=F),]
head(ST_norm_Cerc_Sex_noSB)
ST_norm_Cerc_Z_noSB <-
ST_norm_Cerc_Z_noSB[order(ST_norm_Cerc_Z_noSB$First_base,decreasing=F),]
head(ST_norm_Cerc_Z_noSB)
ST_norm_Cerc_PAR_noSB <-
ST_norm_Cerc_PAR_noSB[order(ST_norm_Cerc_PAR_noSB$First_base,decreasing=F),]
head(ST_norm_Cerc_PAR_noSB)

ST_norm_Cerc_Sex <-
ST_norm_Cerc_Sex[order(ST_norm_Cerc_Sex$First_base,decreasing=F),]
head(ST_norm_Cerc_Sex)
ST_norm_Cerc_Z <- ST_norm_Cerc_Z[order(ST_norm_Cerc_Z$First_base,decreasing=F),]
head(ST_norm_Cerc_Z)
ST_norm_Cerc_PAR <-
ST_norm_Cerc_PAR[order(ST_norm_Cerc_PAR$First_base,decreasing=F),]
head(ST_norm_Cerc_PAR)

# Define colors
colFactor <- as.factor(ST_norm_Cerc_Sex_noSB$Location)
colFactor
colFactor1 <- "Chr_Z"
colFactor1
colFactor2 <- "Chr_PAR"
colFactor2
colF <- rep("red",length(colFactor)) ; colF[which(colFactor==colFactor1)] <-
"wheat" ; colF[which(colFactor==colFactor2)] <- "wheat3"
colF

# Plot expression per gene
plot(as.numeric(paste(ST_norm_Cerc_Sex_noSB$First_base)),
as.numeric(paste(ST_norm_Cerc_Sex_noSB$FMlog_Cerc)),ylim=c(-
1.5,1.5),xlim=c(0,60000000), pch=19, col=colF, ylab="log2(F:M)",adj="0.5",
cex.axis=1.5, cex.lab=1.5, xlab="Position on the Z", main="B. Gene expression
along the Z chromosome - Cercariae", cex.main=2)

# Plot average per 30 gene windows
library(zoo)

par(new=T)
y <- rollapply(as.numeric(paste(ST_norm_Cerc_Sex_noSB$FMlog_Cerc)), width = 30,
by = 1, FUN = mean, align="left")
x <- rollapply(as.numeric(paste(ST_norm_Cerc_Sex_noSB$First_base)), width = 30,
by = 1, FUN = mean, align="left")
plot(x, y, type = "l",lwd=2, col="black", ylim=c(-1.5,1.5), xlim=c(0,60000000),
axes=F, xlab="",ylab="")

par(new=T)
y <- rollapply(as.numeric(paste(ST_norm_Cerc_Sex$FMlog_Cerc)), width = 30, by =
1, FUN = mean, align="left")
x <- rollapply(as.numeric(paste(ST_norm_Cerc_Sex$First_base)), width = 30, by =
1, FUN = mean, align="left")
plot(x, y, type = "l",lwd=1, col="gray45", ylim=c(-1.5,1.5), xlim=c(0,60000000),
axes=F, xlab="",ylab="")
```

```
legend(x=50000000,y=-1.2, box.lty=0, lwd=c(2,1),bg=F, col=c("black","gray45"),
legend=c("FC<2","No filter"))

# Add Z-specific region limits

#Z1
abline(v=3550000, lty=2)
abline(v=13340000, lty=2)
#Z2
abline(v=13860000, lty=2)
abline(v=19650000, lty=2)
#Z3
abline(v=23230000, lty=2)
abline(v=30820000, lty=2)

# Schistosomula

# Sort tables depending on First Base /!\/!\/!\
ST_norm_Som_Sex_noSB <-
ST_norm_Som_Sex_noSB[order(ST_norm_Som_Sex_noSB$First_base,decreasing=F),]
head(ST_norm_Som_Sex_noSB)
ST_norm_Som_Z_noSB <-
ST_norm_Som_Z_noSB[order(ST_norm_Som_Z_noSB$First_base,decreasing=F),]
head(ST_norm_Som_Z_noSB)
ST_norm_Som_PAR_noSB <-
ST_norm_Som_PAR_noSB[order(ST_norm_Som_PAR_noSB$First_base,decreasing=F),]
head(ST_norm_Som_PAR_noSB)

ST_norm_Som_Sex <-
ST_norm_Som_Sex[order(ST_norm_Som_Sex$First_base,decreasing=F),]
head(ST_norm_Som_Sex)
ST_norm_Som_Z <- ST_norm_Som_Z[order(ST_norm_Som_Z$First_base,decreasing=F),]
head(ST_norm_Som_Z)
ST_norm_Som_PAR <-
ST_norm_Som_PAR[order(ST_norm_Som_PAR$First_base,decreasing=F),]
head(ST_norm_Som_PAR)

# Define colors
colFactor <- as.factor(ST_norm_Som_Sex_noSB$Location)
colFactor
colFactor1 <- "Chr_Z"
colFactor1
colFactor2 <- "Chr_PAR"
colFactor2
colF <- rep("red",length(colFactor)) ; colF[which(colFactor==colFactor1)] <-
"wheat" ; colF[which(colFactor==colFactor2)] <- "wheat3"
colF

# Plot expression per gene
plot(as.numeric(paste(ST_norm_Som_Sex_noSB$First_base)),
as.numeric(paste(ST_norm_Som_Sex_noSB$FMlog_Som)),ylim=c(-
1.5,1.5),xlim=c(0,60000000), pch=19, col=colF, ylab="log2(F:M)",adj="0.5",
cex.axis=1.5, cex.lab=1.5, xlab="Position on the Z", main="D. Gene expression
along the Z chromosome - Schistosomula", cex.main=2)

# Plot average per 30 gene windows
library(zoo)

par(new=T)
y <- rollapply(as.numeric(paste(ST_norm_Som_Sex_noSB$FMlog_Som)), width = 30, by
= 1, FUN = mean, align="left")
x <- rollapply(as.numeric(paste(ST_norm_Som_Sex_noSB$First_base)), width = 30,
by = 1, FUN = mean, align="left")
```

```
plot(x, y, type = "l",lwd=2, col="black", ylim=c(-1.5,1.5), xlim=c(0,60000000),
axes=F, xlab="",ylab="")

par(new=T)
y <- rollapply(as.numeric(paste(ST_norm_Som_Sex$FMlog_Som)), width = 30, by = 1,
FUN = mean, align="left")
x <- rollapply(as.numeric(paste(ST_norm_Som_Sex$First_base)), width = 30, by =
1, FUN = mean, align="left")
plot(x, y, type = "l",lwd=1, col="gray45", ylim=c(-1.5,1.5), xlim=c(0,60000000),
axes=F, xlab="",ylab="")

legend(x=50000000,y=-1.2, box.lty=0, lwd=c(2,1),bg=F, col=c("black","gray45"),
legend=c("FC<2","No filter"))

# Add Z-specific region limits

#Z1
abline(v=3550000, lty=2)
abline(v=13340000, lty=2)
#Z2
abline(v=13860000, lty=2)
abline(v=19650000, lty=2)
#Z3
abline(v=23230000, lty=2)
abline(v=30820000, lty=2)

# Adults

# Sort tables depending on First Base /!\/!\/!\
ST_norm_Ad_Sex_noSB <-
ST_norm_Ad_Sex_noSB[order(ST_norm_Ad_Sex_noSB$First_base,decreasing=F),]
head(ST_norm_Ad_Sex_noSB)
ST_norm_Ad_Z_noSB <-
ST_norm_Ad_Z_noSB[order(ST_norm_Ad_Z_noSB$First_base,decreasing=F),]
head(ST_norm_Ad_Z_noSB)
ST_norm_Ad_PAR_noSB <-
ST_norm_Ad_PAR_noSB[order(ST_norm_Ad_PAR_noSB$First_base,decreasing=F),]
head(ST_norm_Ad_PAR_noSB)

ST_norm_Ad_Sex <- ST_norm_Ad_Sex[order(ST_norm_Ad_Sex$First_base,decreasing=F),]
head(ST_norm_Ad_Sex)
ST_norm_Ad_Z <- ST_norm_Ad_Z[order(ST_norm_Ad_Z$First_base,decreasing=F),]
head(ST_norm_Ad_Z)
ST_norm_Ad_PAR <- ST_norm_Ad_PAR[order(ST_norm_Ad_PAR$First_base,decreasing=F),]
head(ST_norm_Ad_PAR)

# Define colors
colFactor <- as.factor(ST_norm_Ad_Sex_noSB$Location)
colFactor
colFactor1 <- "Chr_Z"
colFactor1
colFactor2 <- "Chr_PAR"
colFactor2
colF <- rep("red",length(colFactor)) ; colF[which(colFactor==colFactor1)] <-
"wheat" ; colF[which(colFactor==colFactor2)] <- "wheat3"
colF

# Plot expression per gene
plot(as.numeric(paste(ST_norm_Ad_Sex_noSB$First_base)),
as.numeric(paste(ST_norm_Ad_Sex_noSB$FMlog_Ad)),ylim=c(-
1.5,1.5),xlim=c(0,60000000), pch=19, col=colF, ylab="log2(F:M)",adj="0.5",
cex.axis=1.5, cex.lab=1.5, xlab="Position on the Z", main="F. Gene expression
along the Z chromosome - Adults", cex.main=2)
```

```
# Plot average per 30 gene windows
library(zoo)

par(new=T)
y <- rollapply(as.numeric(paste(ST_norm_Ad_Sex_noSB$FMlog_Ad)), width = 30, by =
1, FUN = mean, align="left")
x <- rollapply(as.numeric(paste(ST_norm_Ad_Sex_noSB$First_base)), width = 30, by
= 1, FUN = mean, align="left")
plot(x, y, type = "l",lwd=2, col="black", ylim=c(-1.5,1.5), xlim=c(0,60000000),
axes=F, xlab="",ylab="")

par(new=T)
y <- rollapply(as.numeric(paste(ST_norm_Ad_Sex$FMlog_Ad)), width = 30, by = 1,
FUN = mean, align="left")
x <- rollapply(as.numeric(paste(ST_norm_Ad_Sex$First_base)), width = 30, by = 1,
FUN = mean, align="left")
plot(x, y, type = "l",lwd=1, col="gray45", ylim=c(-1.5,1.5), xlim=c(0,60000000),
axes=F, xlab="",ylab="")

legend(x=50000000,y=-1.2, box.lty=0, lwd=c(2,1),bg=F, col=c("black","gray45"),
legend=c("FC<2","No filter"))

# Add Z-specific region limits

#Z1
abline(v=3550000, lty=2)
abline(v=13340000, lty=2)
#Z2
abline(v=13860000, lty=2)
abline(v=19650000, lty=2)
#Z3
abline(v=23230000, lty=2)
abline(v=30820000, lty=2)

# B.4.4. Expression along the Z, per sex and stage (FIGURE 2)

# Cercariae

par(mar=c(5,5,3,5))
x <- rollapply(as.numeric(paste(ST_norm_Cerc_Sex$First_base)), width = 50, by =
1, FUN = mean, align="left")
y1 <- rollapply(log2(as.numeric(paste(ST_norm_Cerc_Sex$Fmean_Cerc))), width =
50, by = 1, FUN = mean, align="left")
plot(x, y1, type = "l",lwd=2, ylim=c(2,4.5), col="hotpink3",
ylab="log2(RPKM)",xlim=c(0,60000000),adj="0.5", cex.axis=1.5, cex.lab=1.5,
xlab="Position on the Z", main="Gene expression along the Z chromosome -
Cercariae")
par(new=T)
y2 <- rollapply(log2(as.numeric(paste(ST_norm_Cerc_Sex$Mmean_Cerc))), width =
50, by = 1, FUN = mean, align="left")
plot(x, y2, type = "l",lwd=2,  ylim=c(2,4.5), col="lightcyan3", xlab="",
ylab="", xlim=c(0,60000000),axes=F)
par(new=T)
y <- rollapply(as.numeric(paste(ST_norm_Cerc_Sex$FMlog_Cerc)), width = 50, by =
1, FUN = mean, align="left")
x <- rollapply(as.numeric(paste(ST_norm_Cerc_Sex$First_base)), width = 50, by =
1, FUN = mean, align="left")
plot(x, y, type = "l",lwd=2, col="black", ylim=c(-1,1), xlim=c(0,60000000),
axes=F, xlab="",ylab="")
axis(4, ylim=c(-1,1), cex.axis=1.5, cex.lab=2.5)
mtext("log2(F:M)",side=4,line=2.5, cex=1.5)
abline(v=3550000, lty=2)
abline(v=13340000, lty=2)
abline(v=13860000, lty=2)
```

```
abline(v=19650000, lty=2)
abline(v=23230000, lty=2)
abline(v=30820000, lty=2)
legend(x=50000000,y=1, box.lty=0, bty="o", lwd=c(2,2,2),
col=c("lightcyan3","hotpink3","black"), legend=c("male","female","F:M ratio"))

abline(h=0, lty=1)

# Schistosomula

par(mar=c(5,5,3,5))
x <- rollapply(as.numeric(paste(ST_norm_Som_Sex$First_base)), width = 50, by =
1, FUN = mean, align="left")
y1 <- rollapply(log2(as.numeric(paste(ST_norm_Som_Sex$Fmean_Som))), width = 50,
by = 1, FUN = mean, align="left")
plot(x, y1, type = "l",lwd=2, ylim=c(2,4.5), col="hotpink3",
ylab="log2(RPKM)",xlim=c(0,60000000),Somj="0.5", cex.axis=1.5, cex.lab=1.5,
xlab="Position on the Z", main="Gene expression along the Z chromosome -
SChistosomula")
par(new=T)
y2 <- rollapply(log2(as.numeric(paste(ST_norm_Som_Sex$Mmean_Som))), width = 50,
by = 1, FUN = mean, align="left")
plot(x, y2, type = "l",lwd=2,  ylim=c(2,4.5), col="lightcyan3", xlab="",
ylab="", xlim=c(0,60000000),axes=F)
par(new=T)
y <- rollapply(as.numeric(paste(ST_norm_Som_Sex$FMlog_Som)), width = 50, by = 1,
FUN = mean, align="left")
x <- rollapply(as.numeric(paste(ST_norm_Som_Sex$First_base)), width = 50, by =
1, FUN = mean, align="left")
plot(x, y, type = "l",lwd=2, col="black", ylim=c(-1,1), xlim=c(0,60000000),
axes=F, xlab="",ylab="")
axis(4, ylim=c(-3,3), cex.axis=1.5, cex.lab=2.5)
mtext("log2(F:M)",side=4,line=2.5, cex=1.5)
abline(v=3550000, lty=2)
abline(v=13340000, lty=2)
abline(v=13860000, lty=2)
abline(v=19650000, lty=2)
abline(v=23230000, lty=2)
abline(v=30820000, lty=2)
legend(x=50000000,y=1, box.lty=0, bty="o", lwd=c(2,2,2),
col=c("lightcyan3","hotpink3","black"), legend=c("male","female","F:M ratio"))

abline(h=0, lty=1)

# Adults

par(mar=c(5,5,3,5))
x <- rollapply(as.numeric(paste(ST_norm_Ad_Sex$First_base)), width = 50, by = 1,
FUN = mean, align="left")
y1 <- rollapply(log2(as.numeric(paste(ST_norm_Ad_Sex$Fmean_Ad))), width = 50, by
= 1, FUN = mean, align="left")
plot(x, y1, type = "l",lwd=2, ylim=c(2,4.5), col="hotpink3",
ylab="log2(RPKM)",xlim=c(0,60000000),adj="0.5", cex.axis=1.5, cex.lab=1.5,
xlab="Position on the Z", main="Gene expression along the Z chromosome -
Adults")
par(new=T)
y2 <- rollapply(log2(as.numeric(paste(ST_norm_Ad_Sex$Mmean_Ad))), width = 50, by
= 1, FUN = mean, align="left")
plot(x, y2, type = "l",lwd=2,  ylim=c(2,4.5), col="lightcyan3", xlab="",
ylab="", xlim=c(0,60000000),axes=F)
par(new=T)
y <- rollapply(as.numeric(paste(ST_norm_Ad_Sex$FMlog_Ad)), width = 50, by = 1,
FUN = mean, align="left")
x <- rollapply(as.numeric(paste(ST_norm_Ad_Sex$First_base)), width = 50, by = 1,
```

```
FUN = mean, align="left")
plot(x, y, type = "l",lwd=2, col="black", ylim=c(-1,1), xlim=c(0,60000000),
axes=F, xlab="",ylab="")
axis(4, ylim=c(-3,3), cex.axis=1.5, cex.lab=2.5)
mtext("log2(F:M)",side=4,line=2.5, cex=1.5)
abline(v=3550000, lty=2)
abline(v=13340000, lty=2)
abline(v=13860000, lty=2)
abline(v=19650000, lty=2)
abline(v=23230000, lty=2)
abline(v=30820000, lty=2)
legend(x=50000000,y=1, box.lty=0, bty="o", lwd=c(2,2,2),
col=c("lightcyan3","hotpink3","black"), legend=c("male","female","F:M ratio"))

abline(h=0, lty=1)


# B.4.5. Boxplot of F:M ratio depending of the Z specific region (SUPPLEMENTARY
FIGURE 3)

# Cercariae

ST_norm_Cerc_Z1 <- subset(ST_norm_Cerc_Sex, First_base>3550000 &
First_base<13340000 )
ST_norm_Cerc_Z1 <-
ST_norm_Cerc_Z1[order(ST_norm_Cerc_Z1$First_base,decreasing=F),]
head(ST_norm_Cerc_Z1)
nrow(ST_norm_Cerc_Z1)
ST_norm_Cerc_Z2 <- subset(ST_norm_Cerc_Sex, First_base>13860000 &
First_base<19650000 )
ST_norm_Cerc_Z2 <-
ST_norm_Cerc_Z2[order(ST_norm_Cerc_Z2$First_base,decreasing=F),]
head(ST_norm_Cerc_Z2)
nrow(ST_norm_Cerc_Z2)
ST_norm_Cerc_Z3 <- subset(ST_norm_Cerc_Sex, First_base>23230000 &
First_base<30820000 )
ST_norm_Cerc_Z3 <-
ST_norm_Cerc_Z3[order(ST_norm_Cerc_Z3$First_base,decreasing=F),]
head(ST_norm_Cerc_Z3)
nrow(ST_norm_Cerc_Z3)

boxplot((ST_norm_Cerc_PAR$FMlog_Cerc),(ST_norm_Cerc_Z1$FMlog_Cerc),
(ST_norm_Cerc_Z2$FMlog_Cerc),(ST_norm_Cerc_Z3$FMlog_Cerc), ylim=c(-1.5,1.5),
names = c("PAR","Z1","Z2","Z3"), ylab="log2(F:M)",cex.axis=1.5, cex.lab=1.5,
notch=T, outline = F, col=c("wheat3","wheat","sienna2","wheat"), main="A.
Cercariae",cex.main=2)

boxplot(log2(ST_norm_Cerc_PAR$Fmean_Cerc),log2(ST_norm_Cerc_Z1$Fmean_Cerc),log2(
ST_norm_Cerc_Z2$Fmean_Cerc),log2(ST_norm_Cerc_Z3$Fmean_Cerc), ylim=c(0,10),
names = c("PAR","Z1","Z2","Z3"), ylab="log2(F:M)",cex.axis=1.5, cex.lab=1.5,
notch=T, outline = F, col=c("hotpink4","hotpink3","hotpink3","hotpink3"),
main="A. Cercariae",cex.main=2)
boxplot(log2(ST_norm_Cerc_PAR$Mmean_Cerc),log2(ST_norm_Cerc_Z1$Mmean_Cerc),log2(
ST_norm_Cerc_Z2$Mmean_Cerc),log2(ST_norm_Cerc_Z3$Mmean_Cerc), ylim=c(0,10),
names = c("PAR","Z1","Z2","Z3"), ylab="log2(F:M)",cex.axis=1.5, cex.lab=1.5,
notch=T, outline = F,
col=c("lightcyan4","lightcyan3","lightcyan3","lightcyan3"), main="A.
Cercariae",cex.main=2)

wilcox.test(ST_norm_Cerc_Z1$FMratio_Cerc,ST_norm_Cerc_PAR$FMratio_Cerc)$p.value
median(ST_norm_Cerc_Z1$FMratio_Cerc) / median(ST_norm_Cerc_PAR$FMratio_Cerc)
wilcox.test(ST_norm_Cerc_Z2$FMratio_Cerc,ST_norm_Cerc_PAR$FMratio_Cerc)$p.value
median(ST_norm_Cerc_Z2$FMratio_Cerc) / median(ST_norm_Cerc_PAR$FMratio_Cerc)
wilcox.test(ST_norm_Cerc_Z3$FMratio_Cerc,ST_norm_Cerc_PAR$FMratio_Cerc)$p.value
```

```
median(ST_norm_Cerc_Z3$FMratio_Cerc) / median(ST_norm_Cerc_PAR$FMratio_Cerc)

wilcox.test(ST_norm_Cerc_Z2$FMratio_Cerc,ST_norm_Cerc_Z1$FMratio_Cerc)$p.value
median(ST_norm_Cerc_Z2$FMratio_Cerc) / median(ST_norm_Cerc_Z1$FMratio_Cerc)
wilcox.test(ST_norm_Cerc_Z2$FMratio_Cerc,ST_norm_Cerc_Z3$FMratio_Cerc)$p.value
median(ST_norm_Cerc_Z2$FMratio_Cerc) / median(ST_norm_Cerc_Z3$FMratio_Cerc)
wilcox.test(ST_norm_Cerc_Z3$FMratio_Cerc,ST_norm_Cerc_Z1$FMratio_Cerc)$p.value
median(ST_norm_Cerc_Z3$FMratio_Cerc) / median(ST_norm_Cerc_Z1$FMratio_Cerc)

wilcox.test(ST_norm_Cerc_Z1$Fmean_Cerc,ST_norm_Cerc_PAR$Fmean_Cerc)$p.value
median(ST_norm_Cerc_Z1$Fmean_Cerc) / median(ST_norm_Cerc_PAR$Fmean_Cerc)
wilcox.test(ST_norm_Cerc_Z2$Fmean_Cerc,ST_norm_Cerc_PAR$Fmean_Cerc)$p.value
median(ST_norm_Cerc_Z2$Fmean_Cerc) / median(ST_norm_Cerc_PAR$Fmean_Cerc)
wilcox.test(ST_norm_Cerc_Z3$Fmean_Cerc,ST_norm_Cerc_PAR$Fmean_Cerc)$p.value
median(ST_norm_Cerc_Z3$Fmean_Cerc) / median(ST_norm_Cerc_PAR$Fmean_Cerc)

wilcox.test(ST_norm_Cerc_Z1$Mmean_Cerc,ST_norm_Cerc_PAR$Mmean_Cerc)$p.value
median(ST_norm_Cerc_Z1$Mmean_Cerc) / median(ST_norm_Cerc_PAR$Mmean_Cerc)
wilcox.test(ST_norm_Cerc_Z2$Mmean_Cerc,ST_norm_Cerc_PAR$Mmean_Cerc)$p.value
median(ST_norm_Cerc_Z2$Mmean_Cerc) / median(ST_norm_Cerc_PAR$Mmean_Cerc)
wilcox.test(ST_norm_Cerc_Z3$Mmean_Cerc,ST_norm_Cerc_PAR$Mmean_Cerc)$p.value
median(ST_norm_Cerc_Z3$Mmean_Cerc) / median(ST_norm_Cerc_PAR$Mmean_Cerc)

# Schistosomula
ST_norm_Som_Z1 <- subset(ST_norm_Som_Sex, First_base>3550000 &
First_base<13340000 )
ST_norm_Som_Z1 <- ST_norm_Som_Z1[order(ST_norm_Som_Z1$First_base,decreasing=F),]
head(ST_norm_Som_Z1)
ST_norm_Som_Z2 <- subset(ST_norm_Som_Sex, First_base>13860000 &
First_base<19650000 )
ST_norm_Som_Z2 <- ST_norm_Som_Z2[order(ST_norm_Som_Z2$First_base,decreasing=F),]
head(ST_norm_Som_Z2)
ST_norm_Som_Z3 <- subset(ST_norm_Som_Sex, First_base>23230000 &
First_base<30820000 )
ST_norm_Som_Z3 <- ST_norm_Som_Z3[order(ST_norm_Som_Z3$First_base,decreasing=F),]
head(ST_norm_Som_Z3)

boxplot((ST_norm_Som_PAR$FMlog_Som),(ST_norm_Som_Z1$FMlog_Som),
(ST_norm_Som_Z2$FMlog_Som),(ST_norm_Som_Z3$FMlog_Som), ylim=c(-1.5,1.5), names =
c("PAR","Z1","Z2","Z3"), ylab="log2(F:M)",cex.axis=1.5, cex.lab=1.5, notch=T,
outline = F, col=c("wheat3","wheat","sienna2","wheat"), main="C.
Schistosomula",cex.main=2)
boxplot(log2(as.numeric(paste(ST_norm_Som_PAR$Fmean_Som))),log2(as.numeric(paste
(ST_norm_Som_Z1$Fmean_Som))),log2(as.numeric(paste(ST_norm_Som_Z2$Fmean_Som))),l
og2(as.numeric(paste(ST_norm_Som_Z3$Fmean_Som))), ylim=c(0,10), names =
c("PAR","Z1","Z2","Z3"), ylab="log2(F:M)",cex.axis=1.5, cex.lab=1.5, notch=T,
outline = F, col=c("hotpink4","hotpink3","hotpink3","hotpink3"), main="B.
Schistosomula",cex.main=2)
boxplot(log2(as.numeric(paste(ST_norm_Som_PAR$Mmean_Som))),log2(as.numeric(paste
(ST_norm_Som_Z1$Mmean_Som))),log2(as.numeric(paste(ST_norm_Som_Z2$Mmean_Som))),l
og2(as.numeric(paste(ST_norm_Som_Z3$Mmean_Som))), ylim=c(0,10), names =
c("PAR","Z1","Z2","Z3"), ylab="log2(F:M)",cex.axis=1.5, cex.lab=1.5, notch=T,
outline = F, col=c("lightcyan4","lightcyan3","lightcyan3","lightcyan3"),
main="B. Schistosomula",cex.main=2)

wilcox.test(ST_norm_Som_Z1$FMratio_Som,ST_norm_Som_PAR$FMratio_Som)$p.value
median(ST_norm_Som_Z1$FMratio_Som) / median(ST_norm_Som_PAR$FMratio_Som)
wilcox.test(ST_norm_Som_Z2$FMratio_Som,ST_norm_Som_PAR$FMratio_Som)$p.value
median(ST_norm_Som_Z2$FMratio_Som) / median(ST_norm_Som_PAR$FMratio_Som)
wilcox.test(ST_norm_Som_Z3$FMratio_Som,ST_norm_Som_PAR$FMratio_Som)$p.value
median(ST_norm_Som_Z3$FMratio_Som) / median(ST_norm_Som_PAR$FMratio_Som)

wilcox.test(ST_norm_Som_Z1$Fmean_Som,ST_norm_Som_PAR$Fmean_Som)$p.value
median(ST_norm_Som_Z1$Fmean_Som) / median(ST_norm_Som_PAR$Fmean_Som)
```

```
wilcox.test(ST_norm_Som_Z2$Fmean_Som,ST_norm_Som_PAR$Fmean_Som)$p.value
median(ST_norm_Som_Z2$Fmean_Som) / median(ST_norm_Som_PAR$Fmean_Som)
wilcox.test(ST_norm_Som_Z3$Fmean_Som,ST_norm_Som_PAR$Fmean_Som)$p.value
median(ST_norm_Som_Z3$Fmean_Som) / median(ST_norm_Som_PAR$Fmean_Som)

wilcox.test(ST_norm_Som_Z1$Mmean_Som,ST_norm_Som_PAR$Mmean_Som)$p.value
median(ST_norm_Som_Z1$Mmean_Som) / median(ST_norm_Som_PAR$Mmean_Som)
wilcox.test(ST_norm_Som_Z2$Mmean_Som,ST_norm_Som_PAR$Mmean_Som)$p.value
median(ST_norm_Som_Z2$Mmean_Som) / median(ST_norm_Som_PAR$Mmean_Som)
wilcox.test(ST_norm_Som_Z3$Mmean_Som,ST_norm_Som_PAR$Mmean_Som)$p.value
median(ST_norm_Som_Z3$Mmean_Som) / median(ST_norm_Som_PAR$Mmean_Som)

# Adults
ST_norm_Ad_Z1 <- subset(ST_norm_Ad_Sex, First_base>3550000 & First_base<13340000
)
ST_norm_Ad_Z1 <- ST_norm_Ad_Z1[order(ST_norm_Ad_Z1$First_base,decreasing=F),]
head(ST_norm_Ad_Z1)
ST_norm_Ad_Z2 <- subset(ST_norm_Ad_Sex, First_base>13860000 &
First_base<19650000 )
ST_norm_Ad_Z2 <- ST_norm_Ad_Z2[order(ST_norm_Ad_Z2$First_base,decreasing=F),]
head(ST_norm_Ad_Z2)
ST_norm_Ad_Z3 <- subset(ST_norm_Ad_Sex, First_base>23230000 &
First_base<30820000 )
ST_norm_Ad_Z3 <- ST_norm_Ad_Z3[order(ST_norm_Ad_Z3$First_base,decreasing=F),]
head(ST_norm_Ad_Z3)

boxplot((ST_norm_Ad_PAR$FMlog_Ad),(ST_norm_Ad_Z1$FMlog_Ad),
(ST_norm_Ad_Z2$FMlog_Ad),(ST_norm_Ad_Z3$FMlog_Ad), ylim=c(-1.5,1.5), names =
c("PAR","Z1","Z2","Z3"), ylab="log2(F:M)",cex.axis=1.5, cex.lab=1.5, notch=T,
outline = F, col=c("wheat3","wheat","sienna2","wheat"), main="E. Immature
worms",cex.main=2)
boxplot(log2(as.numeric(paste(ST_norm_Ad_PAR$Fmean_Ad))),log2(as.numeric(paste(S
T_norm_Ad_Z1$Fmean_Ad))),log2(as.numeric(paste(ST_norm_Ad_Z2$Fmean_Ad))),log2(as
.numeric(paste(ST_norm_Ad_Z3$Fmean_Ad))), ylim=c(0,10), names =
c("PAR","Z1","Z2","Z3"), ylab="log2(F:M)",cex.axis=1.5, cex.lab=1.5, notch=T,
outline = F, col=c("hotpink4","hotpink3","hotpink3","hotpink3"), main="C.
Immature worms",cex.main=2)
boxplot(log2(as.numeric(paste(ST_norm_Ad_PAR$Mmean_Ad))),log2(as.numeric(paste(S
T_norm_Ad_Z1$Mmean_Ad))),log2(as.numeric(paste(ST_norm_Ad_Z2$Mmean_Ad))),log2(as
.numeric(paste(ST_norm_Ad_Z3$Mmean_Ad))), ylim=c(0,10), names =
c("PAR","Z1","Z2","Z3"), ylab="log2(F:M)",cex.axis=1.5, cex.lab=1.5, notch=T,
outline = F, col=c("lightcyan4","lightcyan3","lightcyan3","lightcyan3"),
main="C. Immature worms",cex.main=2)

wilcox.test(ST_norm_Ad_Z1$FMratio_Ad,ST_norm_Ad_PAR$FMratio_Ad)$p.value
median(ST_norm_Ad_Z1$FMratio_Ad) / median(ST_norm_Ad_PAR$FMratio_Ad)
wilcox.test(ST_norm_Ad_Z2$FMratio_Ad,ST_norm_Ad_PAR$FMratio_Ad)$p.value
median(ST_norm_Ad_Z2$FMratio_Ad) / median(ST_norm_Ad_PAR$FMratio_Ad)
wilcox.test(ST_norm_Ad_Z3$FMratio_Ad,ST_norm_Ad_PAR$FMratio_Ad)$p.value
median(ST_norm_Ad_Z3$FMratio_Ad) / median(ST_norm_Ad_PAR$FMratio_Ad)

wilcox.test(ST_norm_Ad_Z1$Fmean_Ad,ST_norm_Ad_PAR$Fmean_Ad)$p.value
median(ST_norm_Ad_Z1$Fmean_Ad) / median(ST_norm_Ad_PAR$Fmean_Ad)
wilcox.test(ST_norm_Ad_Z2$Fmean_Ad,ST_norm_Ad_PAR$Fmean_Ad)$p.value
median(ST_norm_Ad_Z2$Fmean_Ad) / median(ST_norm_Ad_PAR$Fmean_Ad)
wilcox.test(ST_norm_Ad_Z3$Fmean_Ad,ST_norm_Ad_PAR$Fmean_Ad)$p.value
median(ST_norm_Ad_Z3$Fmean_Ad) / median(ST_norm_Ad_PAR$Fmean_Ad)

wilcox.test(ST_norm_Ad_Z1$Mmean_Ad,ST_norm_Ad_PAR$Mmean_Ad)$p.value
median(ST_norm_Ad_Z1$Mmean_Ad) / median(ST_norm_Ad_PAR$Mmean_Ad)
wilcox.test(ST_norm_Ad_Z2$Mmean_Ad,ST_norm_Ad_PAR$Mmean_Ad)$p.value
median(ST_norm_Ad_Z2$Mmean_Ad) / median(ST_norm_Ad_PAR$Mmean_Ad)
wilcox.test(ST_norm_Ad_Z3$Mmean_Ad,ST_norm_Ad_PAR$Mmean_Ad)$p.value
median(ST_norm_Ad_Z3$Mmean_Ad) / median(ST_norm_Ad_PAR$Mmean_Ad)
```

```
## B.5. HEATMAPS

# Open normalized table
SuperTable_normalized_all <-
read.table("~/SupplementaryData5.txt",fill=TRUE,header=TRUE) # Normalized table
attached as the Supplementary Data 5 /!\ It should be in a .txt format
head(SuperTable_normalized_all)
nrow(SuperTable_normalized_all) #10787

# AVERAGE BIOLOGICAL REPLICATES & CALCULATE RATIOS

# female average per stage
SuperTable_normalized_all$Fmean_Cerc <-
as.numeric(paste(SuperTable_normalized_all$Cerc_F1+SuperTable_normalized_all$Cer
c_F2))/2
SuperTable_normalized_all$Fmean_Som <-
as.numeric(paste(SuperTable_normalized_all$Som_F1+SuperTable_normalized_all$Som_
F2))/2
SuperTable_normalized_all$Fmean_Ad <-
as.numeric(paste(SuperTable_normalized_all$Ad_F1+SuperTable_normalized_all$Ad_F2
))/2
# male average per stage
SuperTable_normalized_all$Mmean_Cerc <-
as.numeric(paste(SuperTable_normalized_all$Cerc_M1+SuperTable_normalized_all$Cer
c_M2))/2
SuperTable_normalized_all$Mmean_Som <-
as.numeric(paste(SuperTable_normalized_all$Som_M1+SuperTable_normalized_all$Som_
M2))/2
SuperTable_normalized_all$Mmean_Ad <-
as.numeric(paste(SuperTable_normalized_all$Ad_M1+SuperTable_normalized_all$Ad_M2
))/2
# female:male ratio (F:M ratio) per stage
SuperTable_normalized_all$FMratio_Cerc <-
as.numeric(paste(SuperTable_normalized_all$Fmean_Cerc))/as.numeric(paste(SuperTa
ble_normalized_all$Mmean_Cerc))
SuperTable_normalized_all$FMratio_Som <-
as.numeric(paste(SuperTable_normalized_all$Fmean_Som))/as.numeric(paste(SuperTab
le_normalized_all$Mmean_Som))
SuperTable_normalized_all$FMratio_Ad <-
as.numeric(paste(SuperTable_normalized_all$Fmean_Ad))/as.numeric(paste(SuperTabl
e_normalized_all$Mmean_Ad))
# log2(F:M ratio) per stage
SuperTable_normalized_all$FMlog_Cerc <-
log2(as.numeric(paste(SuperTable_normalized_all$FMratio_Cerc)))
SuperTable_normalized_all$FMlog_Som <-
log2(as.numeric(paste(SuperTable_normalized_all$FMratio_Som)))
SuperTable_normalized_all$FMlog_Ad <-
log2(as.numeric(paste(SuperTable_normalized_all$FMratio_Ad)))

## FILTERS

#Filter on expression level
explim <- 1 #Arbitrary minimum level of expression (e.g. null
expression=0.000001)

ST_norm <- subset(SuperTable_normalized_all, Fmean_Cerc>explim &
Mmean_Cerc>explim & Fmean_Som>explim & Mmean_Som>explim & Fmean_Ad>explim &
Mmean_Ad>explim)
nrow(ST_norm)
head(ST_norm)

#Exclude sex bias
```

```
ST_norm <- subset(ST_norm, FMratio_Cerc<2 & FMratio_Cerc>0.5 & FMratio_Som<2 &
FMratio_Som>0.5 & FMratio_Ad<2 & FMratio_Ad>0.5)
nrow(ST_norm)

#Select Z specific genes
ST_norm_Z <- subset(ST_norm, Location=="Chr_Z")
ST_norm_Z <- ST_norm_Z[order(ST_norm_Z$FMratio_Cerc,decreasing=F),]
head(ST_norm_Z)
nrow(ST_norm_Z)

ST_norm_Z1 <- subset(ST_norm_Z, First_base>3550000 & First_base<13340000 )
ST_norm_Z1 <- ST_norm_Z1[order(ST_norm_Z1$FMratio_Cerc,decreasing=F),]
head(ST_norm_Z1)
ST_norm_Z2 <- subset(ST_norm_Z, First_base>13860000 & First_base<19650000 )
ST_norm_Z2 <- ST_norm_Z2[order(ST_norm_Z2$FMratio_Cerc,decreasing=F),]
head(ST_norm_Z2)
ST_norm_Z3 <- subset(ST_norm_Z, First_base>23230000 & First_base<30820000 )
ST_norm_Z3 <- ST_norm_Z3[order(ST_norm_Z3$FMratio_Cerc,decreasing=F),]
head(ST_norm_Z3)

ST_norm_Aut <- subset(ST_norm, Location=="Chr_1" |Location=="Chr_2" |
Location=="Chr_3" |Location=="Chr_4" |Location=="Chr_5" |Location=="Chr_6" |
Location=="Chr_7" )
nrow(ST_norm_Aut)
ST_norm_PAR <- subset(ST_norm, Location=="Chr_PAR")
nrow(ST_norm_PAR)

library(RColorBrewer)
coul = colorRampPalette(brewer.pal(8, "PRGn"))(25)

library(gplots)

# heatmap for Z
table_heatmap <- subset(ST_norm_Z,select=c("FMlog_Cerc","FMlog_Som","FMlog_Ad"))
row.names(table_heatmap) <- ST_norm_Z$Gene_ID
head(table_heatmap)
nrow(table_heatmap)
heatmap.2(as.matrix(table_heatmap),col=coul,tracecol="black",key=TRUE,keysize=1.
5, labCol=c("Cerc.","Som.","Ad."),cexCol=1,cexRow=0.3)

table_heatmap <-
subset(ST_norm_Z1,select=c("FMlog_Cerc","FMlog_Som","FMlog_Ad"))
row.names(table_heatmap) <- ST_norm_Z1$Gene_ID
head(table_heatmap)
nrow(table_heatmap)
heatmap.2(as.matrix(table_heatmap),col=coul,tracecol="black",key=TRUE,keysize=1.
5, labCol=c("Cerc.","Som.","Ad."),cexCol=1,cexRow=0.5)

table_heatmap <-
subset(ST_norm_Z2,select=c("FMlog_Cerc","FMlog_Som","FMlog_Ad"))
row.names(table_heatmap) <- ST_norm_Z2$Gene_ID
head(table_heatmap)
nrow(table_heatmap)
heatmap.2(as.matrix(table_heatmap),col=coul,tracecol="black",key=TRUE,keysize=1.
5, labCol=c("Cerc.","Som.","Ad."),cexCol=1,cexRow=0.5)

table_heatmap <-
subset(ST_norm_Z3,select=c("FMlog_Cerc","FMlog_Som","FMlog_Ad"))
row.names(table_heatmap) <- ST_norm_Z3$Gene_ID
head(table_heatmap)
nrow(table_heatmap)
heatmap.2(as.matrix(table_heatmap),col=coul,tracecol="black",key=TRUE,keysize=1.
5, labCol=c("Cerc.","Som.","Ad."),cexCol=1,cexRow=0.5)
```

```
# heatmap for Aut
table_heatmap <-
subset(ST_norm_Aut,select=c("FMlog_Cerc","FMlog_Som","FMlog_Ad"))
row.names(table_heatmap) <- ST_norm_Aut$Gene_ID
head(table_heatmap)
nrow(table_heatmap)
heatmap.2(as.matrix(table_heatmap),col=coul,tracecol="black",key=TRUE,keysize=1.
5, labCol=c("Cerc.","Som.","Ad."),cexCol=1,cexRow=0.3)

# heatmap for PAR
table_heatmap <-
subset(ST_norm_PAR,select=c("FMlog_Cerc","FMlog_Som","FMlog_Ad"))
row.names(table_heatmap) <- ST_norm_PAR$Gene_ID
head(table_heatmap)
nrow(table_heatmap)
heatmap.2(as.matrix(table_heatmap),col=coul,tracecol="black",key=TRUE,keysize=1.
5, labCol=c("Cerc.","Som.","Ad."),cexCol=1,cexRow=0.3)

# heatmap for all genome
table_heatmap <- subset(ST_norm,select=c("FMlog_Cerc","FMlog_Som","FMlog_Ad"))
row.names(table_heatmap) <- ST_norm$Gene_ID
head(table_heatmap)
nrow(table_heatmap)
heatmap.2(as.matrix(table_heatmap),col=coul,tracecol="black",key=TRUE,keysize=1.
5, labCol=c("Cerc.","Som.","Ad."),cexCol=1,cexRow=0.3)
```
